# Solar wind stream interaction regions throughout the heliosphere

**DOI:** 10.1007/s41116-017-0011-z

**Published:** 2018-01-26

**Authors:** Ian G. Richardson

**Affiliations:** 1 GPHI and Department of Astronomy, University of Maryland, College Park, MD 20742 USA; 2 Code 672, NASA Goddard Space Flight Center, Greenbelt, MD 20771 USA

**Keywords:** Solar wind, High-speed streams, Corotating interaction regions, Heliosphere

## Abstract

This paper focuses on the interactions between the fast solar wind from coronal holes and the intervening slower solar wind, leading to the creation of stream interaction regions that corotate with the Sun and may persist for many solar rotations. Stream interaction regions have been observed near 1 AU, in the inner heliosphere (at $\sim 0.3$–1 AU) by the Helios spacecraft, in the outer and distant heliosphere by the Pioneer 10 and 11 and Voyager 1 and 2 spacecraft, and out of the ecliptic by Ulysses, and these observations are reviewed. Stream interaction regions accelerate energetic particles, modulate the intensity of Galactic cosmic rays and generate enhanced geomagnetic activity. The remote detection of interaction regions using interplanetary scintillation and white-light imaging, and MHD modeling of interaction regions will also be discussed.

## Introduction

**Fig. 1:**
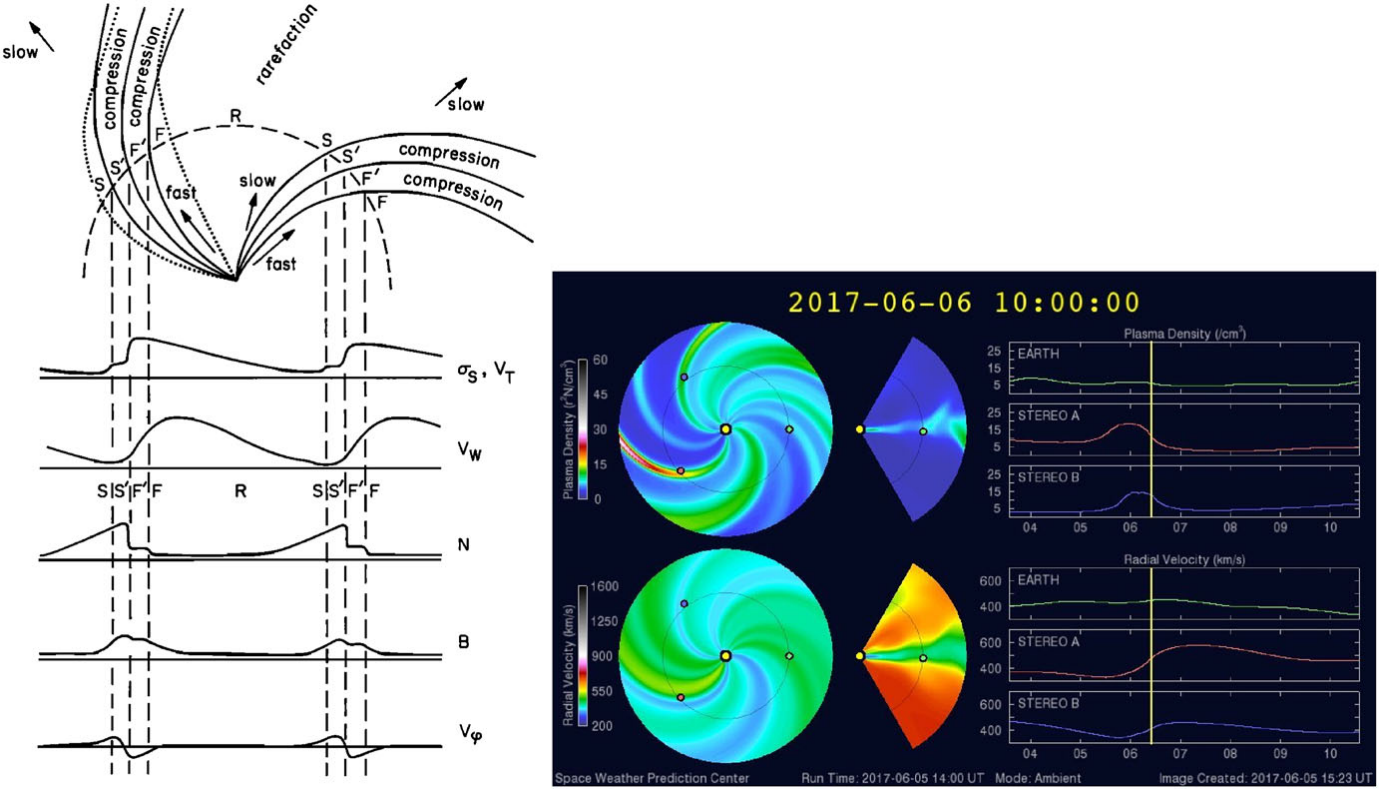
Left: Schematic of two high-speed streams corotating with the Sun and the associated variations in several plasma parameters at 1 AU: Thermal temperature ($V_T$), magnetic field fluctuation level ($\sigma _s$); solar wind speed ($V_W$); density (N); magnetic field intensity (B); and transverse component of the solar wind velocity ($V_\phi$). The regions indicated are: the unperturbed slow solar wind (S), compressed, accelerated slow solar wind (S$^{\prime }$), compressed, decelerated fast solar wind (F$^{\prime }$), unperturbed fast solar wind (F), and a rarefaction (R). S$^{\prime }$ and F$^{\prime }$ form the interaction region, and the stream interface is at the S$^{\prime }$–F$^{\prime }$ boundary. Dotted lines indicate magnetic field lines in the slow and fast solar wind which thread into the interaction region beyond 1 AU. Image reproduced with permission from Belcher and Davis (1971), copyright by AGU. Right: Screenshot from the NOAA Space Weather Prediction Center website (http://www.swpc.noaa.gov/products/wsa-enlil-solar-wind-prediction) showing the density (top row) and solar wind speed (bottom row) predicted by the WSA-ENLIL model (Odstrčil 2003)

A solar wind stream interaction region (SIR) is formed by the interaction of a stream of high-speed solar wind originating in a “coronal hole” at the Sun with the preceding slower solar wind. The interaction forms a region of compressed plasma—the stream interaction region—along the leading edge of the stream, which, due to the rotation of the Sun, is twisted approximately into an Archimedean spiral. Since the source coronal holes tend to be long-lived, often persisting for many months, the interaction regions and high-speed streams tend to sweep past an observer at regular intervals of approximately the solar rotation period ($\sim 27$ days as viewed from Earth). Hence, the interaction regions are frequently referred to as “corotating” interaction regions (CIRs). The left-hand panel of Fig. 1 from Belcher and Davis (1971) shows a schematic of two high-speed streams corotating with the Sun, viewed from above the north pole of the Sun, and the associated variations in the solar wind parameters at 1 AU. The increases in plasma density *N* and magnetic field strength *B* are indicative of compressed plasma in the vicinity of the positive gradient in the solar wind speed ($V_w$) at the stream leading edge and form the interaction region, which follows an approximately Archimedean-spiral configuration. Some other features of this figure will be discussed below. The right-hand panel of Fig. 1 shows similar features in an magneto-hydrodynamic (MHD) model of the solar wind from the NOAA Space Weather Prediction Center website (http://www.swpc.noaa.gov/products/wsa-enlil-solar-wind-prediction). The data in the top row show the solar wind density in the ecliptic plane, in a meridional plane including the Earth, and as time series at the Earth and at the widely-separated STEREO A and B spacecraft (yellow, red and blue circles in the equatorial plane figure). The solar wind speed is shown in the bottom row in similar formats. At the time of the vertical yellow line, both STEREO spacecraft were predicted to be encountering interaction regions associated with the leading edges of two different streams, indicated by the spiral density enhancements sweeping past each spacecraft in the equatorial plane figure.

In this review, stream interaction regions will be discussed mainly from an observational viewpoint. A brief history of the discovery of corotating high-speed streams, including how they accounted for earlier observations of recurrent geomagnetic activity, will be given in Sect. 2. The characteristics of stream interaction regions near 1 AU will then be summarized in Sect. 3, followed by discussion of interaction regions in the inner heliosphere (defined in this paper as inside the orbit of Earth; Sect. 4), outer heliosphere (Sect. 5), and out of the ecliptic (Sect. 6). Subsequent sections discuss the acceleration of charged particles and modulation of galactic cosmic rays in the vicinity of interaction regions (Sect. 7), geomagnetic activity associated with interaction regions (Sect. 8), STEREO spacecraft observations (Sect. 9), remote sensing observations of interaction regions (Sect. 10), MHD modeling (Sect. 11), and outstanding issues (Sect. 12). Note that in this review, we use the general term “stream interaction region”, while being aware that some authors (e.g., Jian et al. 2006) use this term to distinguish a stream that is observed on only one solar rotation from a “corotating” interaction region that is seen on more than one rotation.

In the spirit of a “Living Review”, the intention is to revise this paper periodically, for example to add new results or references to important work that has been overlooked. Therefore, the reader is invited to provide feedback and other material which will help to increase the usefulness of this review.

**Fig. 2:**
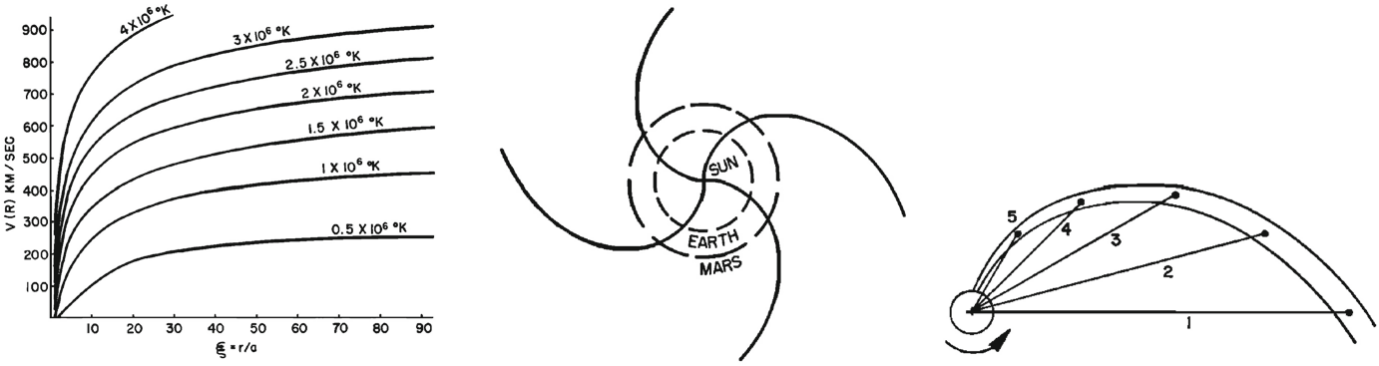
Left: Spherically-symmetric hydrodynamic expansion velocity of an isothermal solar corona as a function of *r* / *a*, where *a* is the radius of the Sun. Center: Projection onto the solar equatorial plane of magnetic field lines carried outward by a solar wind flow of $10^3$ km s$^{-1}$. Right: Development of an Archimedean spiral by a solar wind stream originating at a point on the surface of the Sun. Dots indicate the location of plasma emitted radially on days 1–5. Images reproduced with permission from [left, center] Parker (1959); and [right] Dessler (1967) (adapted from Chapman and Bartels 1940), copyright by AGU

## The discovery of stream interaction regions

### Parker’s theory of the solar wind

Observations of accelerations in comet tails (Biermann 1951, 1952, 1957) suggesting the existence of a gas flowing away radially from the Sun at speeds of $\sim 500$–1500 km s$^{-1}$ helped to inspire the solar wind theory of Parker (1958), which proposed a supersonic, radial, expansion of the solar corona. The left-hand panel in Fig. 2 shows the expansion speeds of several hundreds of km s$^{-1}$ implied by this theory for various coronal temperatures as a function of distance from the Sun (in solar radii, $R_s$; the Earth is at $\sim 215\,R_s$). As a consequence of this expansion, solar magnetic fields are dragged out by the expanding flow. Rotation of the Sun with a sidereal period of 25.38 days then twists the magnetic field lines into Archimedean spirals (center panel of Fig. 2), a configuration also previously proposed by Chapman (1929)—the right-hand panel in Fig. 2 (Dessler 1967, adapted from Chapman and Bartels 1940) shows the locations of particles in a flow emitted from a point on the rotating Sun on days 1–5. Note that although the flow is emitted radially, the locus of the stream traced by the tips of the arrows (also followed by magnetic fields dragged out by the flow) is a spiral. A familiar analogy is the flow pattern from a rotating garden sprinkler.

The spiral interplanetary magnetic field lines in the Parker model are of the form $r - r_o = - V(\phi - \phi _o)/(\Omega \cos \theta )$, where *r* is the heliocentric distance, *V* is the solar wind speed, $\Omega$ is the solar angular velocity, $\theta$ and $\phi$ are the heliolatitude and heliolongitude of the observer, and $r_o$ and $\phi _o$ are the heliocentric distance and heliolongitude of the initial plasma position at the Sun. At low latitudes, streamlines are inclined at an angle $\psi = \arctan (r\Omega /V)$ to the outward radial direction. At 1 AU (149,597,871 km), for a 400 km s$^{-1}$ solar wind, and a 25.38-day sidereal solar rotation period, $r\Omega = 429$ km s$^{-1}$ and $\psi = 47^\circ$. For 800 km s$^{-1}$ solar wind, $\psi$ decreases to $28^\circ$. Thus, magnetic field lines in faster solar wind follow spirals that are less tightly wound. The field lines in the Parker model also lie on cones of constant latitude. See Owens and Forsyth (2013) for a review of the heliospheric magnetic field.

**Fig. 3:**
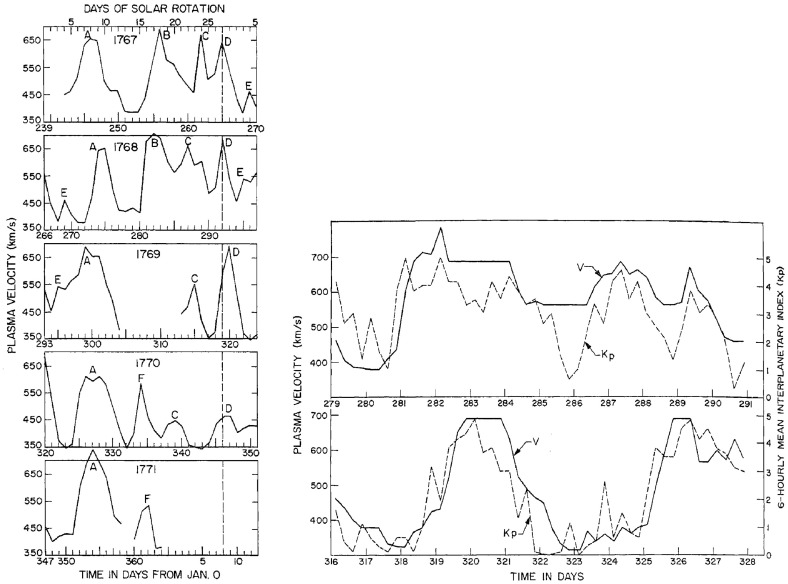
Left: Mariner 2 observations of the solar wind speed from August 29, 1962 to January 3, 1963 organized in 27-day intervals plus a 4 day overlap with the next interval, showing the recurring pattern of high and low speed solar wind present at this time. Recurring high-speed streams are indicated by letters. Right: Mariner 2 observations of two intervals of variable solar wind speed (solid line) showing the strong correlation with the *Kp* geomagnetic index (dashed line, corrected for the Earth-spacecraft delay time). Images reproduced with permission from Snyder et al. (1963), copyright by AGU

### Discovery of corotating high-speed streams

In 1962, the Mariner 2 spacecraft (http://www.jpl.nasa.gov/mariner2/) en route to Venus established that a solar wind with properties similar to those predicted by Parker (1958) was continuously present (Neugebauer and Snyder 1962). However, the solar wind speed was not constant but was observed to vary in a range from $\sim 400$ to 700 km s$^{-1}$. The left-hand panel of Fig. 3 shows Mariner 2 solar wind speed observations (Snyder et al. 1963) from August 29, 1962 to January 3, 1963 arranged in intervals of 27 days (the solar rotation period from the viewpoint of the moving spacecraft) plus a 4 day overlap with the next interval. The large variability of the solar wind speed is evident, with some transitions between slow and fast solar wind occurring over intervals of only of the order of a day. Furthermore, the pattern of higher-speed and slower streams tends to recur on successive solar rotations; recurring higher-speed streams are indicated by letters. In some cases, these were observed on at least four or five solar rotations, indicating that they were long-lived ($\gg$ solar rotation period) spatial features corotating with the Sun. However, the speed profiles do show some development and evolution from one occurrence to the next, such as the declines in the peak speeds of streams C and D between the third and fourth rotations.

**Fig. 4:**
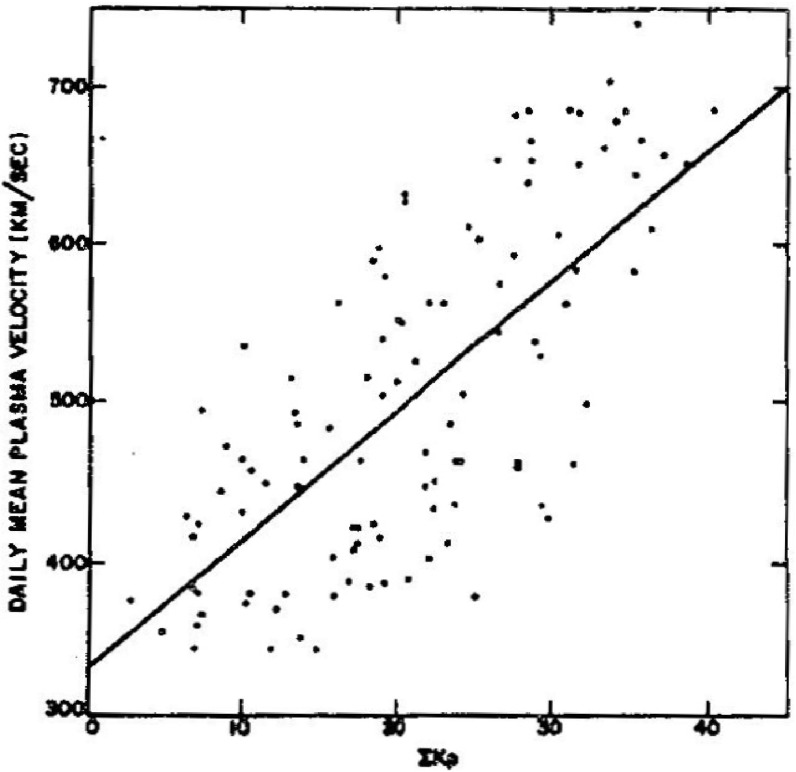
Correlation between daily averages of the solar wind speed and *Kp* geomagnetic index based on Mariner 2 observations. Image reproduced with permission from Snyder et al. (1963), copyright by AGU, who obtained a fit *V* (km s$^{-1})=(8.44\pm 0.74)\Sigma K_p + (330\pm 17)$, where the $K_p$ sum is over a day

Snyder et al. (1963) also noticed that the pattern of fast and slow speed solar wind was closely associated with variations in the level of geomagnetic activity, as illustrated in the right-hand panel of Fig. 3 which shows the clear correlation between the solar wind speed (solid line) and the *Kp* geomagnetic index (dashed line; see Bartels et al. (1939); Menvielle and Berthelier (1991) for information on *Kp*) for two periods of variable solar wind speed in 1962 (a small timing correction is applied to allow for the separation between Mariner 2 and Earth). The correlation between daily values of *Kp* and solar wind speed obtained by Snyder et al. (1963) is shown in Fig. 4. They used this relationship to produce a “corrected” speed which showed no clear variation with the heliocentric distance of the spacecraft, leading them to conclude that there was no detectable gradient in the solar wind speed between 0.7 AU, the heliocentric distance of Mariner 2, and 1 AU. This is consistent with the trend towards $\sim$constant speed with increasing heliocentric distance predicted by Parker’s theory (left-hand panel of Fig. 2). For a personal account of the discovery of the solar wind, see Neugebauer (1997).

**Fig. 5:**
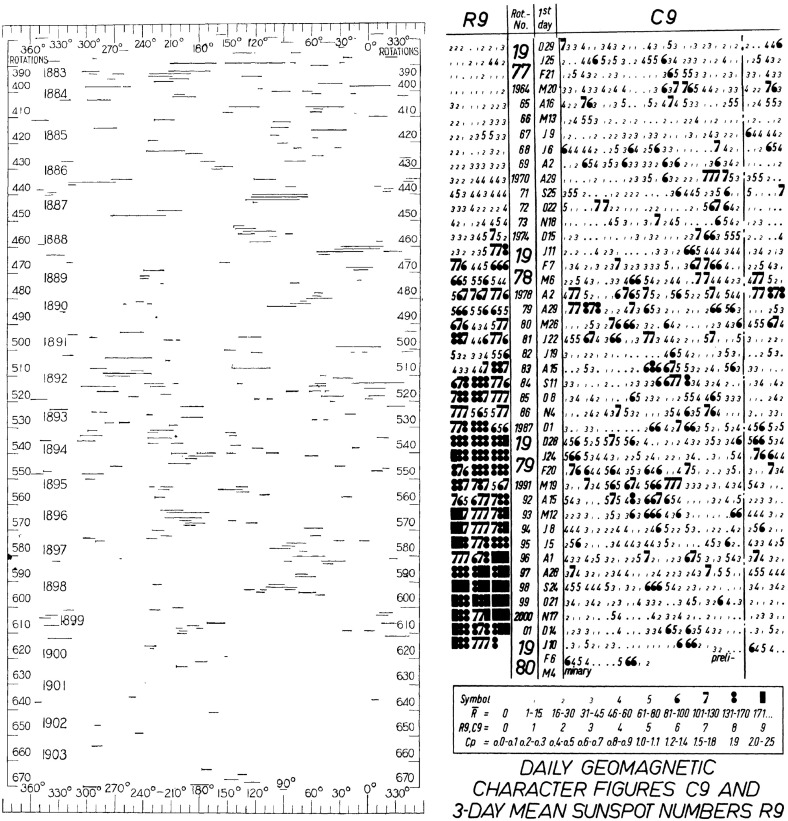
Left: Distribution of geomagnetic disturbances in 1882–1903 according to the heliographic longitude of the center of the Sun’s disk at time of their commencement. Image reproduced with permission from Maunder (1904), copyright by RAS. Right: 27-day (Bartels rotation) stackplot of the daily *C*9 geomagnetic index and 3-day mean sunspot number (*R*9) for 1977–early 1980. Note that intervals of recurrent activity tend to be most prominent at times of lower solar activity levels, while isolated sporadic storms are more prominent at higher activity levels. A current figure in a similar format is available at http://www-app3.gfz-potsdam.de/kp_index/r9c9.pdf

Snyder et al. (1963) also pointed out a connection between the enhanced geomagnetic activity associated with the passage of high-speed solar wind past the Earth, the recurrence of these fast solar wind streams at the solar rotation period, and the similarly recurring intervals of enhanced geomagnetic activity previously identified by Maunder (1904). The left-hand panel of Fig. 5 shows intervals of enhanced geomagnetic activity in 1882–1903 arranged by the phase of the solar rotation period, from Maunder’s paper. Many intervals of recurrent activity, often extending over multiple solar rotations, may be identified. Maunder (1904) makes several prescient conclusions about the driver of this type of geomagnetic activity, including:

- “The origin of our magnetic disturbances lies in the Sun; ...This is clear from the manner in which those disturbances mark out the solar rotation period; not the actual sidereal period but the synodic period; the period as it appears to us.”
- “The areas giving rise to our magnetic disturbances are definite and restricted areas...”
- “The influence proceding from the Sun ...does not act equally in all directions ...but its action is confined to a definite and very restricted direction.”
- The occurrence of geomagnetic storms at intervals of one or more synodic rotation periods of the Sun “can only be explained by supposing that the earth has encountered, time after time, a definite stream ...which continually supplied from one and the same area of the Sun’s surface appears to us to be rotating with the same speed as the area from which it arises.”
- “The average diameter of such streams may be roughly estimated from noting the time which a average storm lasts [30 h]. This would imply an average diameter for those stream lines of $20^\circ$” occupying “about $1/130^{th}$ part of the sphere instead of the whole of it...The streamlines giving rise to the magnetic disturbances are not necessarily truly radial in direction.”

Maunder (1904) also pointed out that because the disturbances only involved restricted regions on the Sun and were highly directional, this removed the objection of Lord Kelvin, in his Presidential address to the Royal Society of London in 1892 (Kelvin 1892), that the amount of energy required to generate an 8-h geomagnetic storm, *if radiated equally in all directions*, would exceed the total amount of energy emitted by the Sun as light and heat in 4 months.

**Fig. 6:**
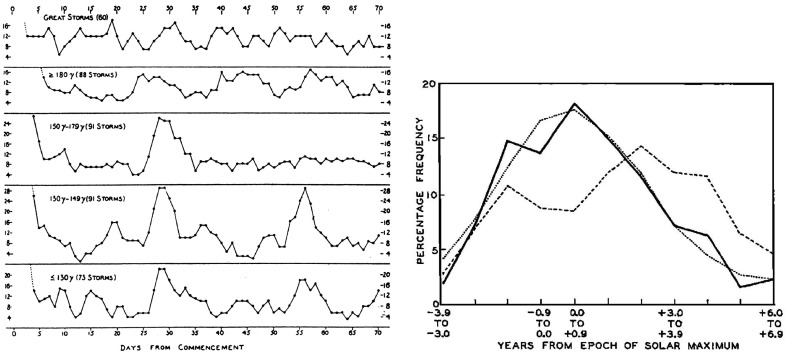
Left: Occurrence of recurrent geomagnetic activity one and two solar rotations following weaker storms (lower graphs) but not the strongest storms (upper graphs). Image reproduced with permission from Greaves and Newton (1929), copyright by RAS. Right: Occurrence rates of 235 storms with (solid curve) or 420 weaker storms without (dashed curve) storm sudden commencements compared with the mean sunspot number (dotted) in 1878–1952, indicating that weaker storms are most frequent during the declining phase of the solar cycle whereas stronger storms tend to follow the solar cycle. Image reproduced with permission from Newton and Milsom (1954), copyright by AGU

### Coronal holes: the source regions of high-speed solar wind streams

The high-speed solar wind streams corotating with the Sun discovered by Mariner 2 clearly fitted the specifications for the driver of recurrent geomagnetic activity inferred by Maunder (1904). However, the source regions on the Sun remained unclear. Snyder et al. (1963) concluded that “no strong correlation existed between sunspot number or the 10.7 cm flux” (a close proxy for the sunspot number, e.g., Tapping and Charrois 1994; Sect. 3.4 of Hathaway 2015) “and plasma velocity” in the interval they studied.

The sources of recurrent geomagnetic storms, now evidently closely linked to high-speed streams, had already been a topic of much previous speculation. Greaves and Newton (1929) noted that weaker geomagnetic storms were most likely to be recurrent whereas larger storms were not. The left-hand panel of Fig. 6 illustrates their results. Starting from days on which storms in a particular size range were occurring, for a period of 70 days afterwards, the percentage of cases in which storm conditions were observed on each day is shown. It is evident that geomagnetic activity tends to increase temporarily around one and two solar rotations after the weaker storms, but not following the strongest storms. Similar conclusions were reached by Newton and Milsom (1954) who divided storms into those stronger storms associated with storm sudden commencements (SSCs) [related to the arrival of interplanetary shocks by Gold (1955)], which were not recurrent, and weaker storms not associated with SSCs, that tended to be recurrent.

Bartels (1932, 1940) used 27-day stacked plots of the geomagnetic *C*9 index (see http://ccmc.gsfc.nasa.gov/modelweb/solar/ap.html for details of *C*9) to investigate the occurrence of recurrent geomagnetic activity in 1906–1931. A plot in a similar format to that used by Bartels is shown in the right-hand panel of Fig. 5 for 1977–early 1980, where the density of the printed numbers visually indicates the daily level of geomagnetic activity on the right-hand side of the figure, and the daily sunspot number (*R*9) on the left. Bartels noted, as is evident in this figure, that recurrent geomagnetic activity is dominant during intervals of lower solar activity and may be observed even in the absence of sunspots; the unknown solar regions giving rise to this activity were termed ‘M’ (“mystery”) regions. On the other hand, solar maximum is dominated by “sporadic” storms associated with the presence of sunspots. Consistent with this picture, Newton and Milsom (1954) also demonstrated that the occurrence rate of storms without SSCs, unlike those stronger storms with SSCs, does not track the sunspot number but peaks during the decay of the cycle, as shown in the right-hand panel of Fig. 6. (Note also in this figure that the storm rate decreases temporarily near solar maximum, a feature that will be discussed further in Sect. 8.) In addition, Allen (1944) demonstrated that recurrent storms show a seasonal effect, being larger around the equinoxes in March and September when the Earth is at its largest latitudinal separation from the solar equator, suggesting that the M-regions were north and south of the equator. Furthermore, some recurrent storms persisted for more than a year even when no sunspots were present, and two or three M-regions were typically present during a solar rotation.

Following the development of a new instrument to measure weak photospheric magnetic fields using the Zeeman effect, Babcock and Babcock (1955) and Simpson et al. (1955) found that recurrent geomagnetic activity during seven solar rotations tended to peak when a persistent region of weak, unipolar, magnetic field at low latitudes was on the western hemisphere of the Sun as viewed from Earth. (Note that by standard convention, the solar western and eastern hemispheres are reversed relative to those of the Earth, i.e., the western solar hemisphere is on the right when viewed from Earth.) This westward bias would clearly be expected from the spiral stream configuration predicted by Parker’s (yet to be developed) solar wind theory if the driver of the activity, and hence the source of the fast solar wind, were related to the weak unipolar field region.

**Fig. 7:**
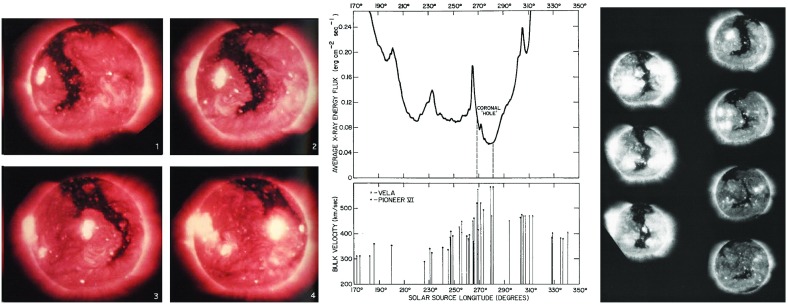
Left: Skylab soft X-ray observations of a coronal hole (dark region) extending from the north-polar regions to the southern hemisphere made $\sim 2$ days apart. Image reproduced with permission from Eddy and Ise (1979), copyright by NASA. Center: Comparison of the X-ray intensity (top, in wavebands at 3–35Åand 44–51Å) in a 4$^{\prime }$ latitude by 4$^{\prime \prime }$ longitude region with measurements of the solar wind speed from the Pioneer VI or Vela spacecraft mapped to the solar source longitude (bottom), showing the depressed X-ray intensity in the coronal hole that is the source of higher-speed solar wind. Image reproduced with permission from Krieger et al. (1973), copyright by D. Reidel. Right: A trans-equatorial coronal hole extending from the north polar coronal hole photographed by the Skylab ASE ATM X-ray Telescope at 3–35 Å and 44–51 Å on successive solar rotations between June 1 (top left) and November 11 (bottom right), 1973. Image reproduced with permission from Zirker (1977), copyright by AGU

The mystery of the nature of M-regions was eventually resolved using observations made during the Skylab mission in 1973–1974 (https://www.nasa.gov/mission_pages/skylab). These revealed regions of weak X-ray emission in the solar corona, termed “coronal holes”. An example [the aptly named “Boot of Italy” coronal hole (Zirker 1977)], observed in a sequence of soft X-ray images taken $\sim 2$ days apart, is shown in the left-hand panel of Fig. 7, where the rotation of the coronal hole with the Sun is clearly evident. (A movie of these observations is available at http://soi.stanford.edu/results/SolPhys200/Hudson/2000/001020/skylab.mpg.) The center panel (Krieger et al. 1973) shows the close association between a region of depressed coronal X-ray flux and higher speed solar wind that has been mapped back to the solar source longitude by assuming Parker spiral stream lines, while the right-hand panel (Zirker 1977) shows a coronal hole observed on seven successive solar rotations in 1973, including the observation in the top right of the left-hand panel. This coronal hole surrounds the north pole and has a narrow extension that crosses the equator, i.e., it is a “trans-equatorial” coronal hole. On the final rotation, the extension has disappeared, leaving what appears to be an isolated coronal hole in the southern hemisphere. This figure illustrates how although coronal holes may be long-lived structures present for multiple rotations, they also develop and evolve with time, in turn influencing the solar wind stream structure in the heliosphere, as will be discussed further below. For further details about coronal holes, see Cranmer (2002, 2009), and references therein.

**Fig. 8:**
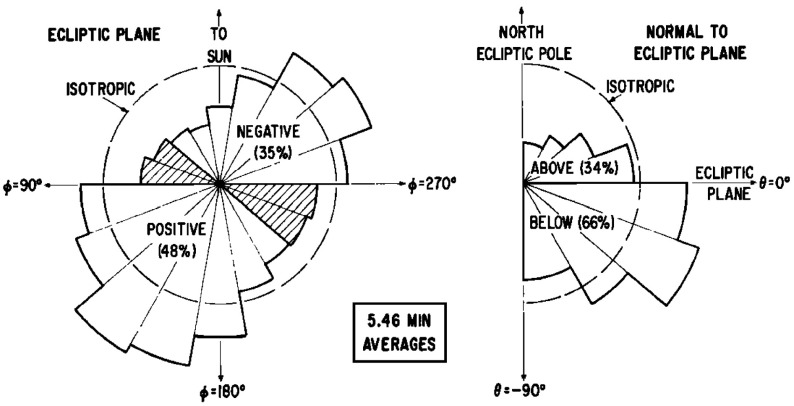
Distributions of the interplanetary magnetic field direction observed by IMP 1 in the ecliptic plane (left), showing the tendency for the field to be aligned towards (“negative”) or away from the Sun (“positive”) along the spiral direction proposed by Parker (1958), and in a plane perpendicular to the ecliptic (right), with a slight southern bias in this particular sample. Image reproduced with permission from Ness and Wilcox (1964), copyright by APS

### Early interplanetary magnetic field observations

Mariner 2 also detected a persistent interplanetary magnetic field that was typically aligned close to the ecliptic, as predicted by Parker (1958), but was also variable in both direction and intensity, ranging from 2 to 10 nT during the period of observations (Coleman et al. 1962). The existence of the predicted Archimedean spiral magnetic field was convincingly demonstrated in observations from IMP 1. Figure 8 from Ness and Wilcox (1964) shows distributions of the interplanetary magnetic field (IMF) direction in the plane of the ecliptic (left) and normal to the ecliptic made between November 27, 1963 and February 17, 1964, covering three solar rotations. The tendency for the field to be closely aligned towards (“negative”) or away from the Sun (“positive”) along the nominal Parker spiral direction (recall from Sect. 2.1 that this is at $\sim 47^\circ$ to the radial direction for 400 km s$^{-1}$ solar wind at 1 AU) is clearly evident, while in this sample, there is a slight southward-directed bias.

The IMP 1 observations also demonstrated the organization of the IMF into “sectors” in which the field is directed predominantly in one direction, either towards or away from the Sun, for several days, then reverses to the opposite direction for several days. This is illustrated in the top panel of Fig. 9 from Wilcox and Ness (1965), which shows the direction of the magnetic field ($+$ $=$ away, − $=$ toward) for 3-h averages during three solar rotations. Note that the transitions between sectors occur relatively abruptly, and the pattern of inward and outward fields recurs at intervals of a solar rotation, indicating that this pattern is corotating with the Sun. During this interval, the IMF had a four-sector structure, with two alternating pairs of inward and outward sectors which are assumed in the figure to follow the spiral field configuration and mapped to regions of weak magnetic field of similar polarity in the photosphere. Dessler (1967) gives a comprehensive review of the development of ideas of the solar wind, early observations, and the theory of the solar wind and interplanetary magnetic field.

**Fig. 9:**
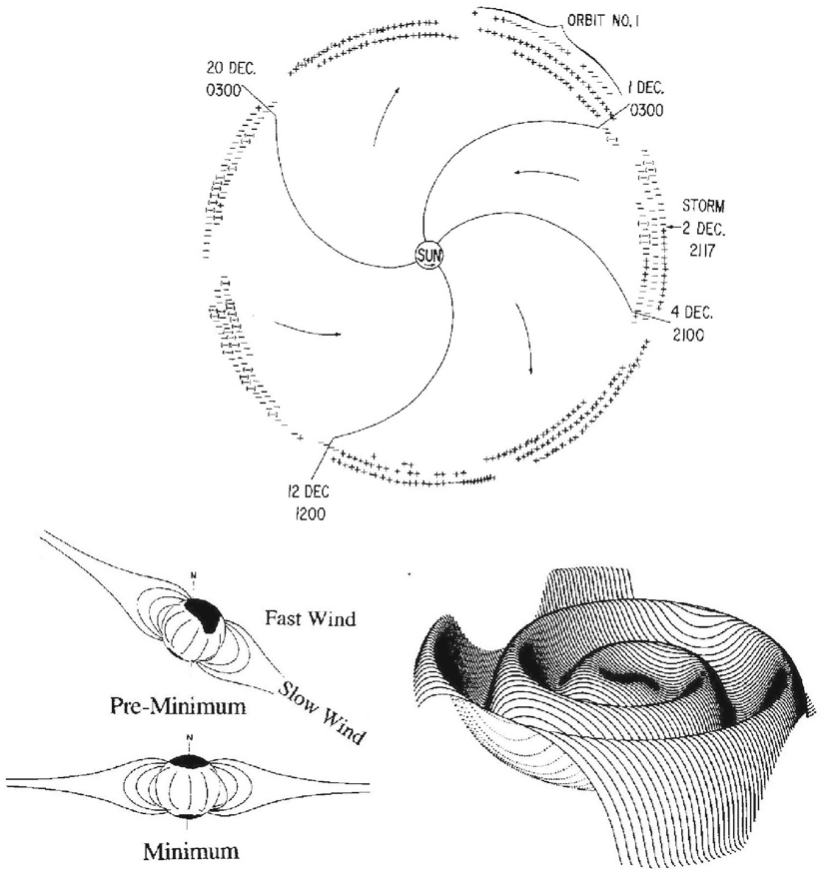
Top: 3-h averages of the IMF direction ($+$ $=$ away from the Sun, − $=$ toward) measured by IMP 1 over three solar rotations, showing the corotating four sector (two away, two toward) structure present at this time. Image reproduced with permission from Wilcox and Ness (1965), copyright by AGU. Bottom left: Temporal changes in the inclination of the solar dipole magnetic field (‘tilt-angle’). Image reproduced with permission from Gosling and Pizzo (1999) (after Hundhausen 1977), copyright by Kluwer. Bottom right: The configuration of the heliospheric current sheet (HCS) in the solar wind for a substantial tilt-angle. Image reproduced with permission from Jokipii and Thomas (1981), copyright by AAS. Crossings of the HCS correspond to sector boundaries as observed in the top panel

**Fig. 10:**
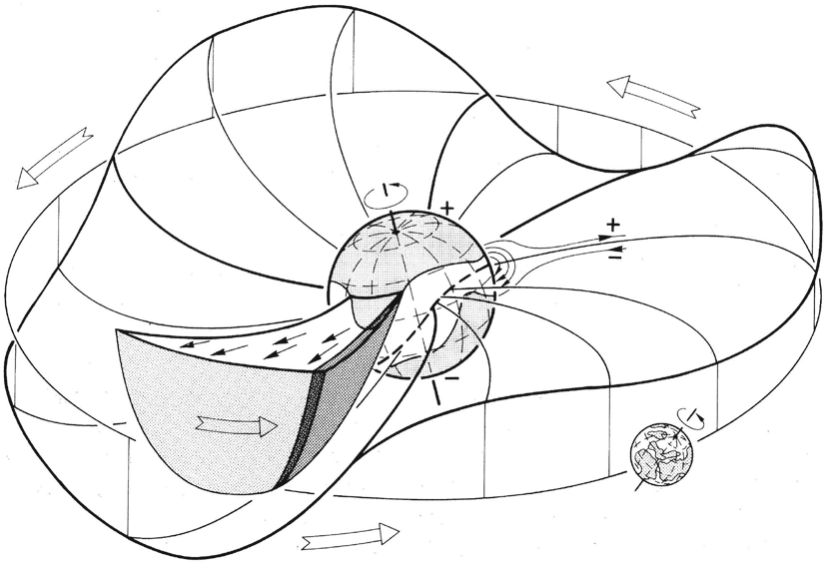
Configuration in the inner heliosphere of a “ballerina skirt” heliospheric current sheet (Alfvén 1977) extending above the streamer belt near solar minimum (for $A>0$ solar magnetic field polarity, i.e., outward field at the north pole), which lies ahead of a high-speed stream (drawn truncated at high latitudes) from an equatorward extension of a northern polar coronal hole. The dark shaded region is the interaction region. Image reproduced with permission from Schwenn (1990), copyright by Springer

Though the terms ‘sector’ and ‘sector boundary’ persist in use, the sector structure is associated with crossings of the heliospheric current sheet (HCS), which is embedded in slow, dense solar wind emerging from the ‘streamer belt’ that typically overlays the solar magnetic equator. The bottom-left panel of Fig. 9 shows how the inclination of the streamer belt varies with time in response to changes in the inclination (‘tilt-angle’) of the solar magnetic dipole with respect to the rotation axis, which is near $0^\circ$ around solar minimum. Figure 10 shows a “ballerina skirt” current sheet (Alfvén 1977) in the inner heliosphere extending above the streamer belt at the Sun lying ahead of, then deflected southward by, a high-speed stream from an equatorward extension of a polar coronal hole. The lower-right panel of Fig. 9 shows an idealized “corrugated” configuration of the HCS extending far out into the solar wind for a substantial tilt-angle (Jokipii and Thomas 1981).

**Fig. 11:**
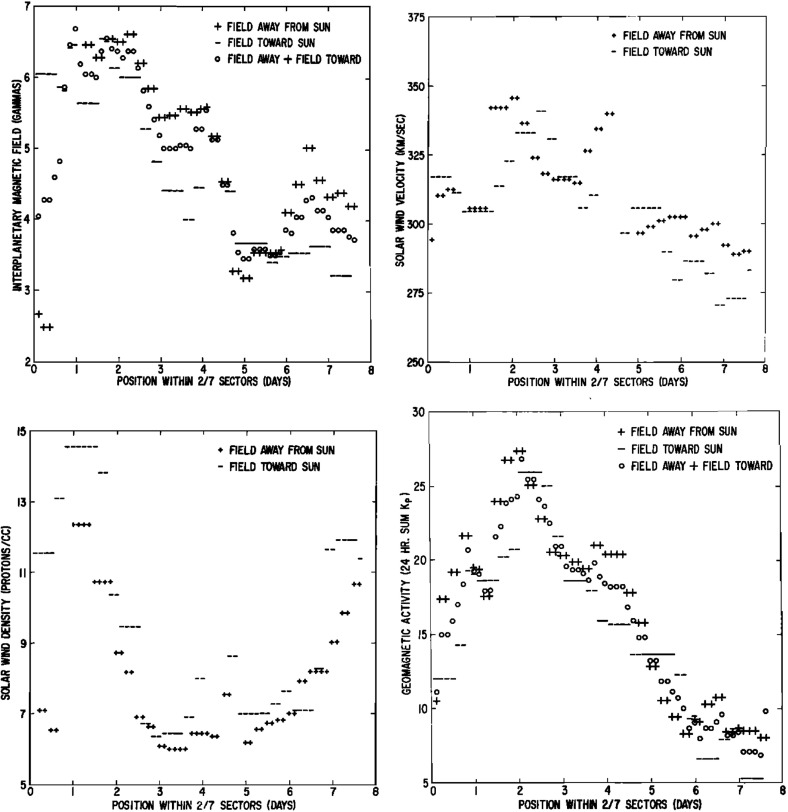
Superposed epoch analyses of (top left) the IMF intensity, (top right) solar wind speed, (bottom left) solar wind density and (bottom right) geomagnetic activity ($K_p$) during the sectors in Fig. 9. Image reproduced with permission from Wilcox and Ness (1965), copyright by AGU

## Stream interaction regions near 1 AU

Wilcox and Ness (1965) also pointed out that the magnetic sector structure orders variations in other solar wind parameters, as illustrated in Fig. 11, which shows superposed epoch analyses (Chree 1913) of several parameters relative to the sector boundary crossing time. In particular, the average IMF intensity (top left) was found to rise rapidly and peak $\sim 1$ day after the crossing, then decay. The solar wind speed rises to peak late on days 1–2, then decays, though there are some observational issues. The density also peaks around day one, then falls to a minimum in the center of the sector before rising again toward the end. Finally, geomagnetic activity increases, like the solar wind speed, to peak late on day 1–day 2, then decays gradually to the end of the sector. Similar patterns were found in both toward and away sectors. These observations clearly point to a large-scale organization, and consistent inter-relationship between solar wind parameters, that is related to the rotation of the Sun and long-lived structures on the Sun. In particular, the density and field enhancements shortly following the sector boundary crossing are suggestive of plasma compression that occurs in the vicinity of the positive speed gradient.

**Fig. 12:**
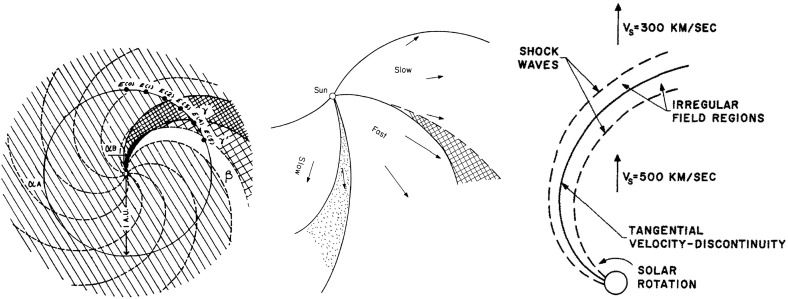
Three sketches of the interaction between slow and fast solar wind streams. Left: Formation of a turbulent compression region $\gamma$ and cavity $\beta$ at leading and trailing edges of a high-speed stream, respectively. Image reproduced with permission from Sarabhai (1963), copyright by AGU. Center: A region of compressed plasma (hatched) at the leading edge of the fast stream and a rarefaction (dotted) at the trailing edge. Image reproduced with permission from Parker (1965a), copyright by D. Reidel. Right: A region of turbulence caused by the Kelvin–Helmholtz instability at the stream interaction, also suggesting the formation of shocks at the boundaries of the interaction region and a tangential discontinuity separating slow and fast solar wind. Image reproduced with permission from Dessler and Fejer (1963), copyright by Elsevier

The close inter-relationship of the variations in solar wind properties found in the IMP 1 data can be explained by considering the interaction of high-speed solar wind from a coronal hole with the preceding slower solar wind. As discussed in Sect. 2.1, spiral field lines and flow stream lines will be less tightly wound in the fast solar wind than in the preceding slower solar wind. According to the frozen in field principle (Alfvén 1943), field lines in the different plasma regimes cannot mix. Instead, the faster flow interacts with and deflects the slower flow to the west, while the slower flow deflects the faster flow to the east. The resulting compression leads to increases in the plasma density and magnetic field intensity, forming the stream interaction region. Such a scenario was considered by several early authors: The left-hand panel of Fig. 12 from Sarabhai (1963) illustrates the compression region $\gamma$ and “cavity” $\beta$ that were expected to be formed ahead of and following a high-speed flow, respectively. The center panel is a sketch from Parker (1965a) (see also Parker 1963) showing a compression region (hatched) at the leading edge of the fast stream and a rarefaction (dotted) at the trailing edge. The right-hand sketch is from Dessler and Fejer (1963). They suggested that the interaction between the slow and fast solar wind would be characterized by turbulence formed by the Kelvin–Helmholtz instability, which could be responsible for generating recurrent geomagnetic activity. Other notable features of this sketch (which we will return to below) are the two shock waves formed at the edges of the interaction region, and the tangential discontinuity separating slow and fast solar wind in the middle of the interaction region.

**Fig. 13:**
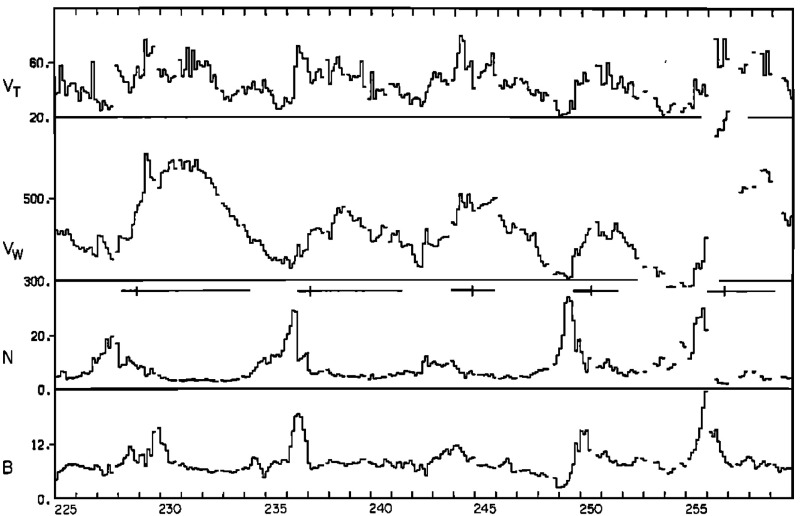
A 35-day interval of Mariner 5 data, showing several high-speed streams separated by periods of slower solar wind. The solar wind thermal speed ($V_T$), solar wind speed ($V_W$), density (*N*) and magnetic field intensity (*B*) are shown. Note that the density peaks tend to occur at or just ahead of the start of the speed gradients, and the magnetic field peaks occur later, within the gradient. The horizontal lines indicate where Alfvén waves were identified, with thicker lines indicating where the strongest waves were observed, in the speed gradients. Image reproduced with permission from Belcher and Davis (1971), copyright by AGU

**Fig. 14:**
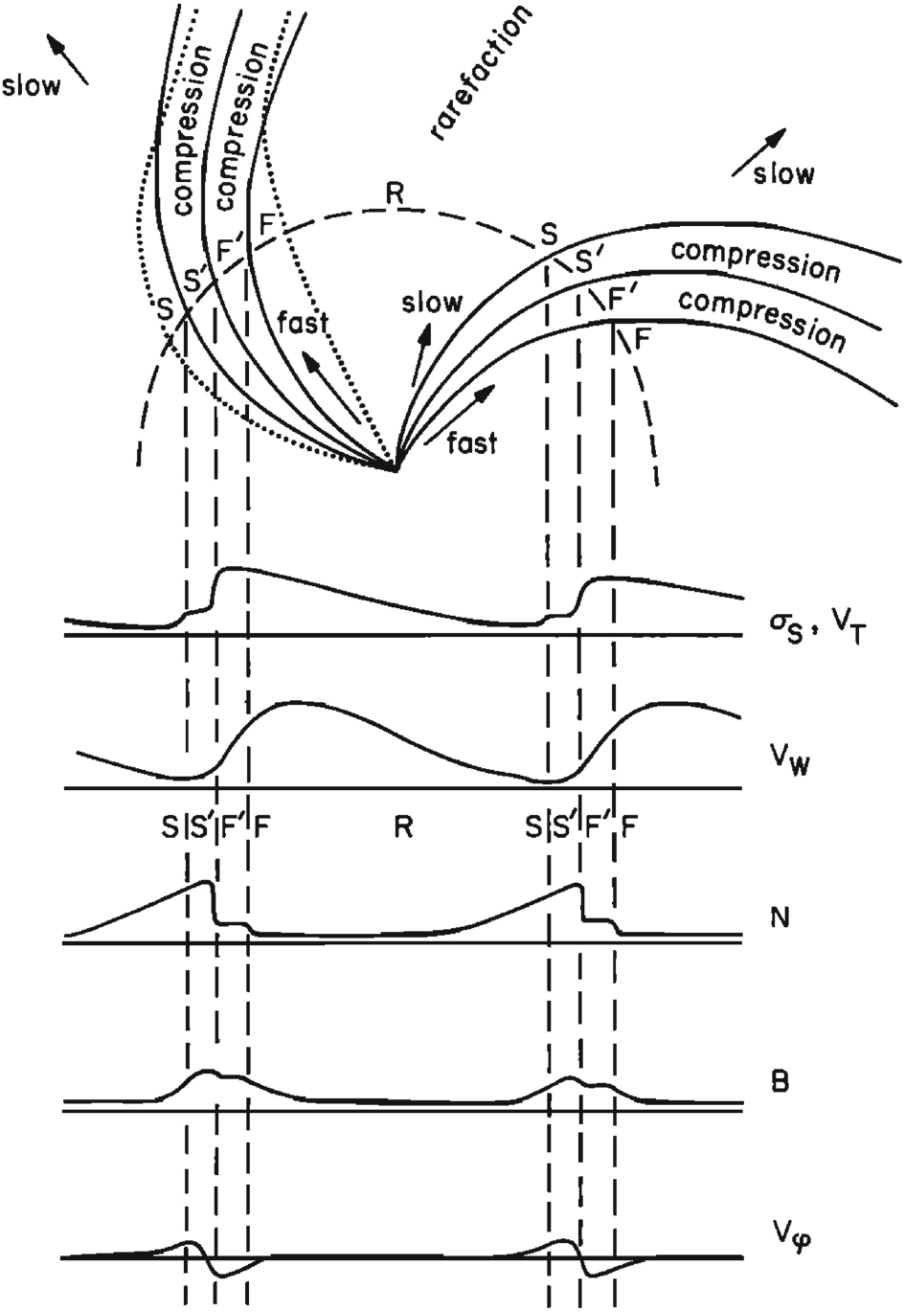
Schematic of two high-speed streams corotating with the Sun and the associated variations in several plasma parameters at 1 AU: Thermal temperature ($V_T$), magnetic field fluctuation level ($\sigma _s$; see Belcher and Davis (1971) for details); solar wind speed ($V_W$); density (N); magnetic field intensity (B); and transverse component of the solar wind velocity ($V_\phi$). The regions indicated are: the unperturbed slow solar wind (S), compressed, accelerated slow solar wind (S$^{\prime }$), the compressed, decelerated fast solar wind (F$^{\prime }$), the unperturbed fast solar wind (F), and a rarefaction in the region of declining solar wind speed (R). S$^{\prime }$ and F$^{\prime }$ form the interaction region, and the stream interface is at the S$^{\prime }$–F$^{\prime }$ boundary. Dotted lines indicate magnetic field lines in the slow and fast solar wind, which thread into the interaction region beyond 1 AU. Image reproduced with permission from Belcher and Davis (1971), copyright by AGU

Figure 13 from Belcher and Davis (1971) shows a 35-day period of Mariner 5 data that includes several alternating intervals of slow and fast solar wind. The other parameters illustrated are the solar wind thermal speed ($V_T$), which shows the usual correlation with solar wind speed ($V_W$) (e.g., Burlaga and Ogilvie 1973; Lopez and Freeman 1986; Matthaeus et al. 2006; Elliott et al. 2012), density (*N*) and magnetic field intensity (*B*). The density and magnetic field enhancements associated with the positive speed gradients, similar to those inferred from the superposed epoch analysis in Fig. 11, are prominent features. Note that the highest densities tend to occur ahead of the strongest magnetic fields within the speed gradient, as is also evident in Fig. 11. Furthermore, the “cavities” following high-speed streams suggested by Sarabhai (1963) (cf. the left-hand panel of Fig. 12) are absent; the solar wind is continually present and the low densities in the declining phases of the streams are more consistent with the rarefactions suggested by Parker (1965a) (center panel in Fig. 12).

Belcher and Davis (1971) summarized the typical profiles of the plasma parameters at 1 AU associated with stream interactions in Fig. 14 (also shown in Fig. 1). The upper part of the figure shows two high-speed streams corotating with the Sun, as viewed from above the north solar pole, with spiral regions of compressed plasma along their leading edges. Dotted lines indicate representative magnetic field lines/streamlines in the slow and fast solar wind that thread into the compression region in the outer heliosphere. Variations in plasma parameters observed as the stream structures corotate past a spacecraft at $\sim 1$ AU are shown in the lower part of the figure. Belcher and Davis (1971) identify four regions: the ambient, undisturbed, slow solar wind (S); slow solar wind, which has been compressed and accelerated by the interaction with the fast solar wind (S$^{\prime }$); fast stream plasma, which as been compressed and decelerated by the interaction with the slow solar wind (F$^{\prime }$), and the ambient, undisturbed, fast-stream plasma (F). The S$^{\prime }$ and F$^{\prime }$ regions form the stream interaction region, characterized by enhanced plasma densities and magnetic field intensities. The plasma pressure $P=Nk(T_e+T_p)+B^2/2\mu _o$, where $T_e$ is the plasma electron temperature, is enhanced within an interaction region, causing it to expand into the ambient solar wind.

**Fig. 15:**
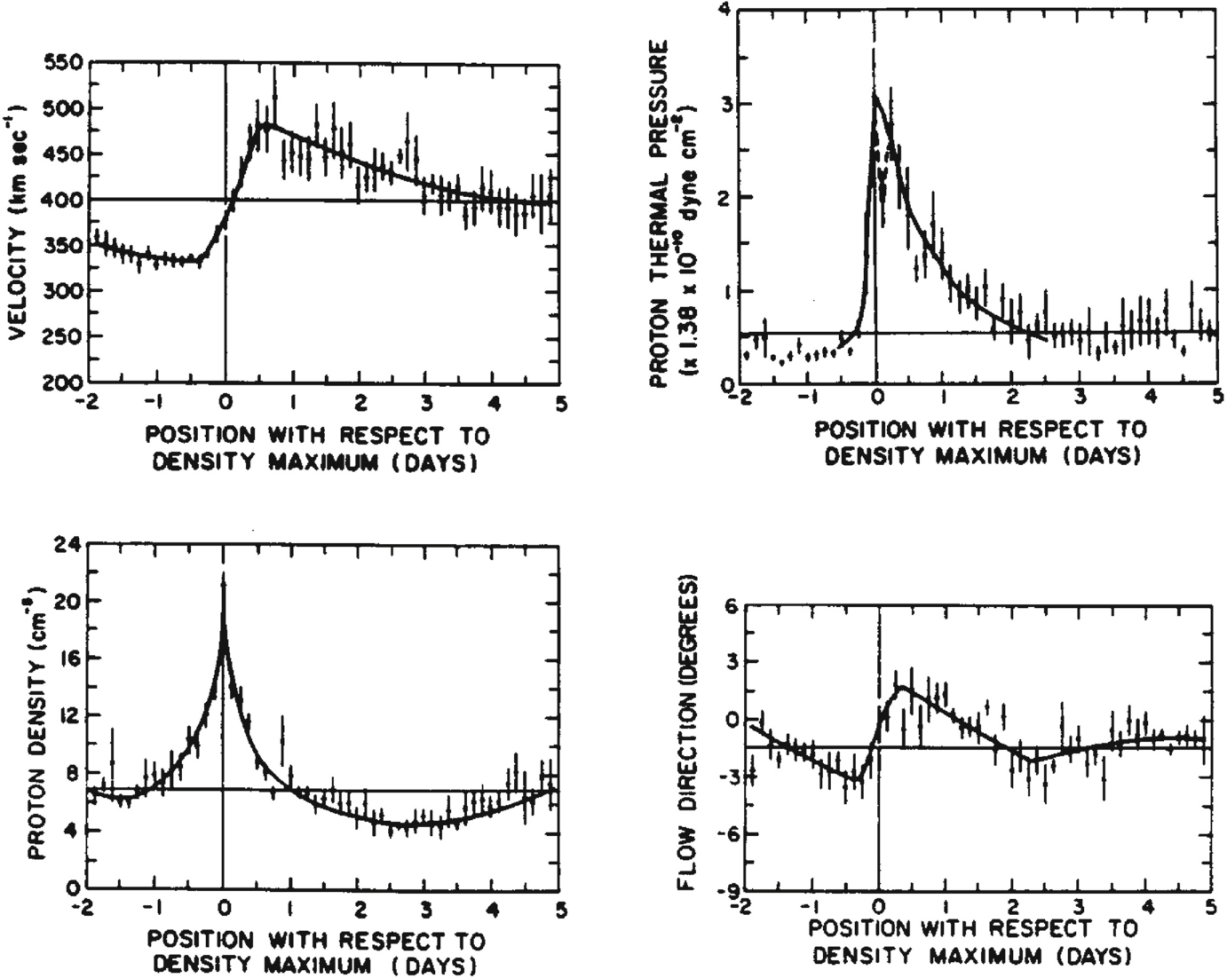
Characteristic temporal variations of the solar wind speed (top left), thermal pressure (top right), proton density (bottom left), and flow direction (bottom right) based on the average of 25 events (Gosling et al. 1972)

Figure 15 (Gosling 1996, adapted from Gosling et al. 1972) shows a superposed epoch analysis of the solar wind plasma parameters in the vicinity of 25 density increases associated with gradients in the solar wind speed, here aligned by the peak density, that largely conforms with the scenario on Fig. 14. Note again that peak density occurs early in the speed gradient, the reason being that densities tend to be larger in the slow solar wind (Feldman et al. 1981; Gosling et al. 1981). The proton thermal pressure ($P_p=NkT_p$) also peaks near peak density, but is highly asymmetric, being lower in the slow solar wind than in the fast solar wind, where the proton temperature is higher. The transverse solar wind flow deflection to the west (negative) then to the east (positive) during the interaction is also evident in the bottom-right panel, with the transition from west to east occurring close to peak density.

**Fig. 16:**
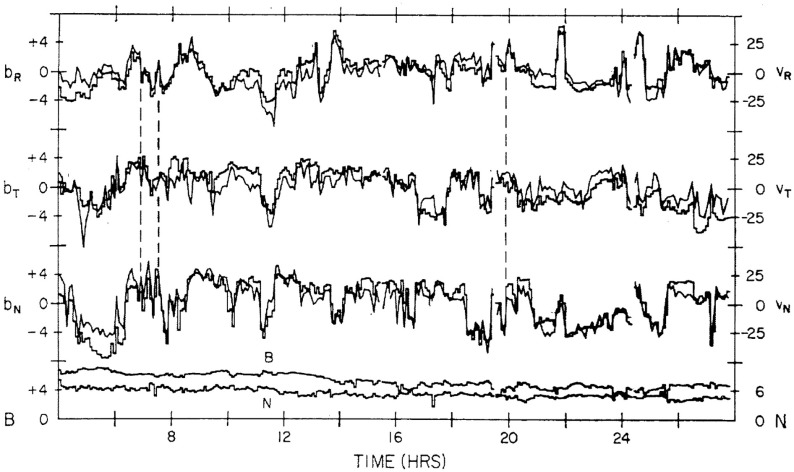
Examples of Alfvénic fluctuations showing correlated variations in the three components of the IMF and solar wind speed in RTN coordinates. The total field strength (*B*) and density (*N*) are also shown. Image reproduced with permission from Belcher and Davis (1971), copyright by AGU

Figure 14 also shows a parameter $\sigma _s$ that represents the level of Alfvénic fluctuations, characterized by correlated variations in the direction of the magnetic field and solar wind velocity related by the Alfvén speed $V_A=B/\sqrt{\mu _o\rho }$ (Alfvén 1942); examples are illustrated in Fig. 16 from Belcher and Davis (1971). The horizontal bars in Fig. 13 indicate that such fluctuations were observed throughout the high-speed streams, with the strongest fluctuations (indicated by thicker bars) occurring within the interaction regions. Belcher and Davis (1971) concluded that since these fluctuations were present throughout high-speed streams, and were propagating outwards, they were generated at the Sun rather than by the stream interaction, in contrast to the earlier proposal of Coleman (1968) that these fluctuations were turbulence generated by the shear in the solar wind speed across the interaction region. Since the Alfvén speed is only around a tenth of the solar wind speed at 1 AU, the Alfvénic fluctuations are convected with the solar wind. Subsequent observations confirm that they are a ubiquitous feature of corotating high-speed streams (e.g., Smith et al. 1995), and may increase in amplitude when convected into the interaction region (e.g., Tsurutani et al. 1995, 2006b).

The boundary between the S$^{\prime }$ and F$^{\prime }$ regions in Fig. 14 marks the “stream interface” between slow and fast solar wind (e.g., Burlaga 1974; Gosling et al. 1978; Schwenn 1990; Forsyth and Marsch 1999; Crooker et al. 1999). The interface is typically characterized by a transition (which may be relatively abrupt) that includes a fall in plasma density (*N* in Fig. 14), because slow solar wind is typically denser than fast solar wind, as well as an increase in the plasma proton temperature ($T_p$, indicated by the proton thermal speed $V_T$ in Fig. 14) across the interface, since faster solar wind has a higher temperature than slow solar wind as noted above. The interface is also indicated by an increase in the“specific entropy”, which is proportional to $T_P/n^{\gamma -1}$, where $\gamma$ is the ratio of specific heats (e.g., Intriligator and Siscoe 1994). In Fig. 14, the magnetic field intensity profile is drawn with a small decline across the interface but observations (e.g., Fig. 13) indicate that the field intensity profile within an interaction region and the change at the interface are variable from event to event. The bottom parameter in Fig. 14 ($V_\phi$) is the transverse component of the solar wind velocity. This indicates that the slow solar wind is deflected toward the west ahead of the interface, while the fast solar wind is deflected toward the east following the interface, passing through the radial direction in the vicinity of the stream interface. A similar pattern is evident in the bottom right panel of Fig. 15.

**Fig. 17:**
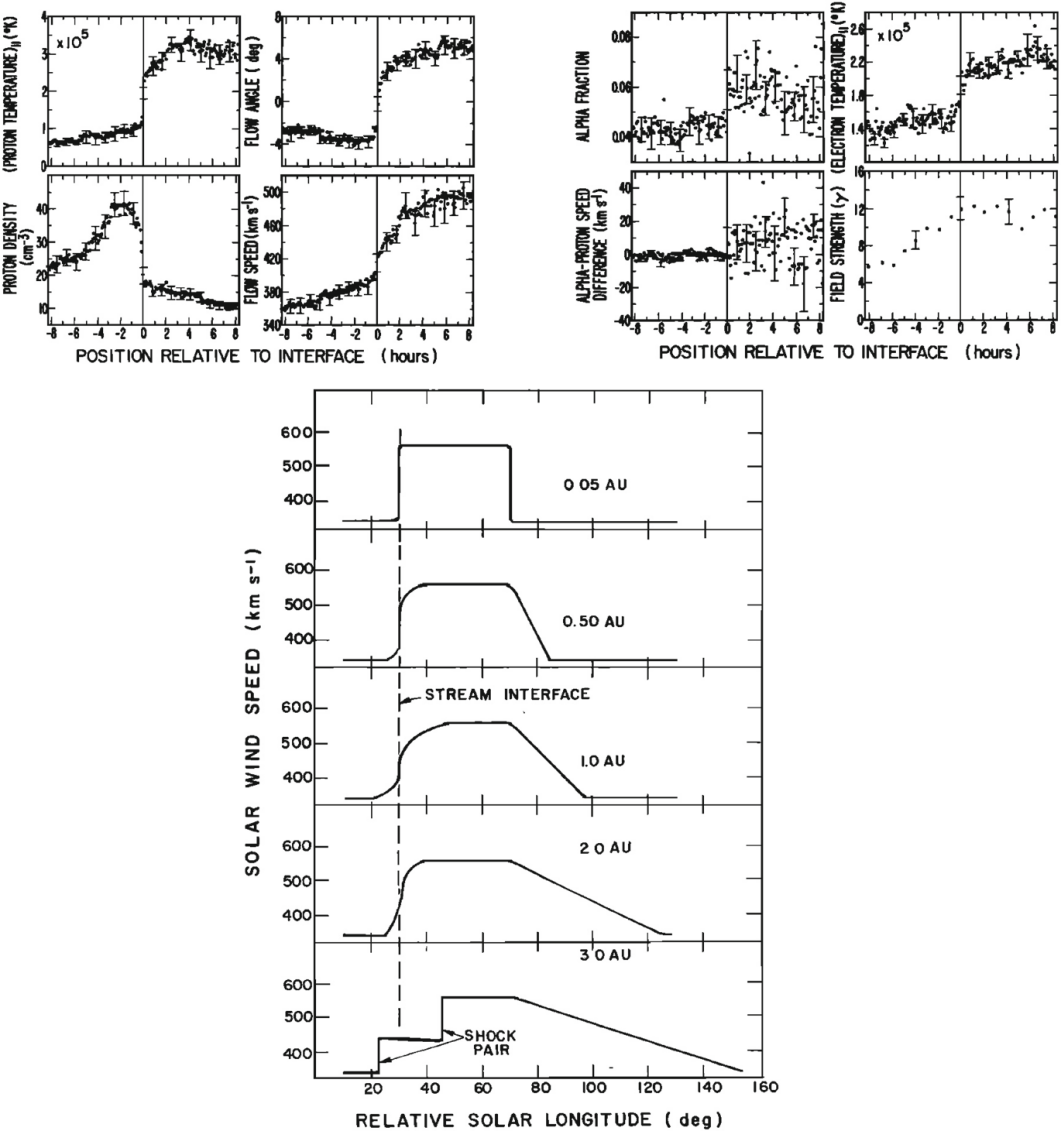
Features of stream interfaces. The top panels show superposed epoch analyses based on 5-min-averaged observations of the solar wind proton temperature, density, flow angle, flow speed, fraction of alpha particles, alpha particle-proton speed difference, electron temperature, and magnetic field strength, in the vicinity of 23 abrupt stream interfaces and aligned at the interface (vertical line). The bottom panel shows the evolution of the stream speed profile with heliocentric distance proposed by Gosling et al. (1978), including erosion of the sharp speed transition present near the Sun due to momentum transfer. Image reproduced with permission from Gosling et al. (1978), copyright by AGU

Belcher and Davis (1971) proposed that the interface originates as a sharp transition between slow and fast flows near the Sun, ideally a tangential discontinuity, which magnetic field lines do not cross, but this view was challenged by Burlaga (1974), who proposed instead that a gradual speed transition near the Sun becomes steepened by the stream interaction, as modeled by Hundhausen and Burlaga (1975). The presence of a sharp transition between different plasma regimes was clearly demonstrated by Gosling et al. (1978) using the superposed epoch analysis shown in the top panels of Fig. 17. This is similar to that shown in Fig. 15 but uses the interface, defined as a discontinuous drop in density and increase in temperature, to align the observations in 23 interaction regions. The abrupt changes in many solar wind parameters at the interface demonstrate that this is a narrow structure that separates originally slow and dense plasma from faster, more rarefied plasma and is a site of a discontinuous shear in the solar wind velocity. Other changes, such as in the alpha-proton ratio (“alpha fraction”), the difference between the alpha and proton flow speeds, and the electron temperature, also indicate that the plasma on each side of the interface is of different origin at the Sun, and hence the interface is not simply a dynamical feature formed through the interaction of the slow and fast streams. Figure 17 also illustrates that while the magnetic field intensity tends to be enhanced in the vicinity of the interface, there is no abrupt change at the interface. Another point noted by Gosling et al. (1978) (not shown in this figure) is that while sector boundaries between regions of opposite magnetic polarity might be expected to be coincident with the interface, this is not usually the case. Rather, sector boundaries were found from 1.5 h to 1.5 days ahead of the interface in all but one case. This is consistent with the results in Fig. 11, indicating that the density enhancement is delayed relative to the sector boundary and also with the scenario in Fig. 10, where the HCS lies in the slow solar wind ahead of the interaction region.

The bottom panel of Fig. 17 shows the erosion with heliocentric distance of the initially sharp speed gradients near the Sun at the leading and trailing edges of a high-speed stream due to momentum transfer between the slow and fast streams, as envisaged by Gosling et al. (1978). It is suggested that the speed transition will evolve into a pair of forward and reverse shocks bounding the expanding interaction region at several AU, the reason being that the magnetosonic speed in the solar wind $V_f=\sqrt{V_A^2+V_s^2}$, where $V_A=B/\sqrt{\mu _o\rho }$ is the Alfvén speed, and $V_s=\sqrt{5P/3\rho }$ is the sound speed ($\rho$ is the plasma mass density), decreases with increasing distance from the Sun so that the expanding boundaries of the interaction region are more likely to steepen into shocks with increasing distance from the Sun. However, such shocks can form by 1 AU. For example, Jian et al. (2006) report that $\sim 17\%$ of interaction regions at 1 AU in 1995–2004 had a forward shock at the leading edge, $\sim 6\%$ had a reverse shock at the trailing edge, and $\sim 1.4\%$ had a forward–reverse shock pair. Note also that in the scenario in the right-hand panel of Fig. 12, shocks were expected to bound the interaction region at all heliocentric distances.

**Fig. 18:**
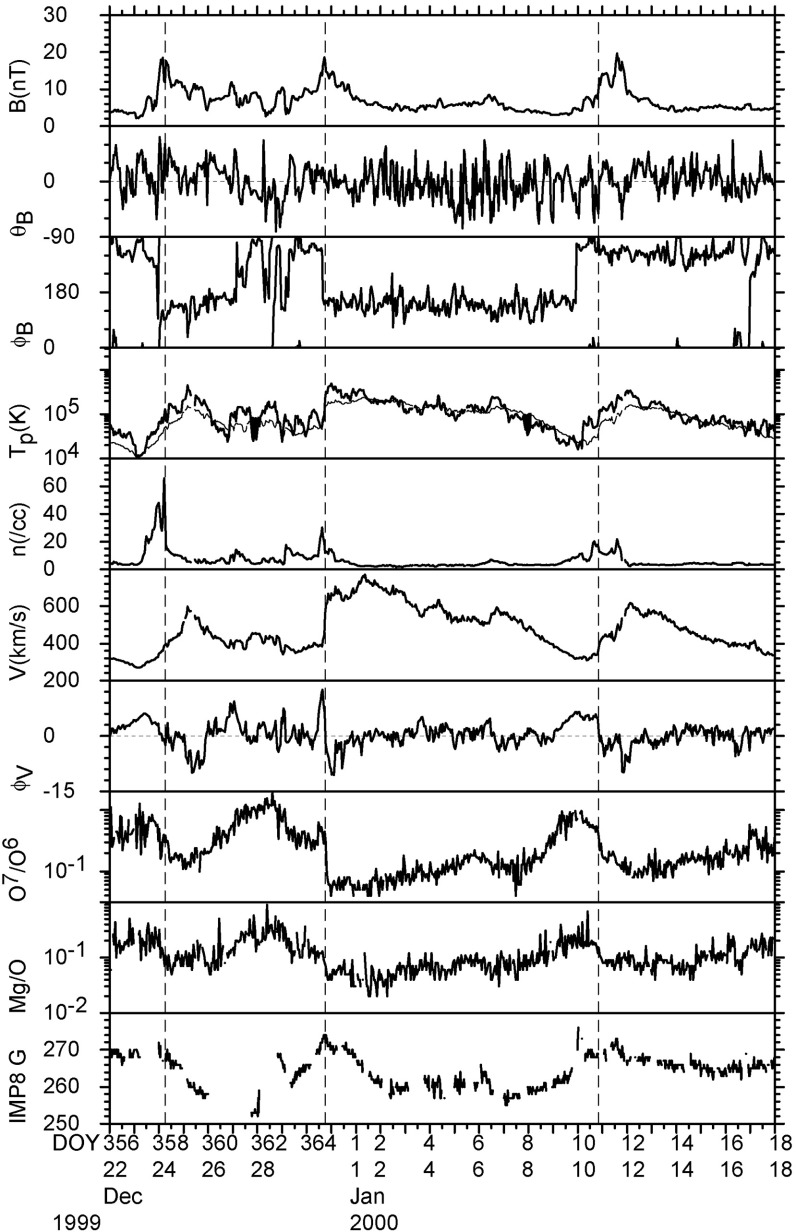
Observations of three high-speed streams with interaction regions at their leading edges made near the Earth during a 27-day period in December 1999–January 2000. The vertical dashed lines indicate the stream interfaces. Solar wind plasma and magnetic field data are from the ACE spacecraft, while the bottom panel shows the modulations in the galactic cosmic-ray intensity indicated by the counting rate of the anti-coincidence guard (G) of the IMP 8 GME instrument. Image reproduced with permission from Richardson (2006), copyright by AGU

Figure 18 shows more recent observations of three corotating high-speed streams with interaction regions at their leading edges observed near Earth during one 27-day period by the ACE spacecraft in December 1999–January 2000 during the ascending phase of solar cycle 23 that exhibit many of the features discussed above. The data illustrated include the magnetic field intensity, polar and azimuthal angles (in GSE coordinates), the plasma proton temperature, density, speed, flow angle, and $\hbox {O}^7$/$\hbox {O}^6$ and Mg/O ratios, all from ACE, and the Galactic cosmic-ray intensity from IMP 8, specifically, the count rate of the GME anti-coincidence guard (Richardson 2004). Dashed vertical lines within the magnetic field intensity and plasma density enhancements associated with the interaction regions indicate stream interface crossings. These are characterized by decreases in density, increases in solar wind speed and proton temperature, and inflections in the solar wind flow angle moving through the radial direction. In addition, the solar wind $O^7/O^6$ and *Mg* / *O* ratios both decrease, reflecting the differences in these parameters in slow and fast solar wind (Geiss et al. 1995b; Wimmer-Schweingruber et al. 1997), in the vicinity of the interface. Since these parameters are determined close to the Sun in the solar wind source region, such variations are additional evidence that the interface is a structural, not a dynamical feature of the solar wind. Figure 18 also shows cosmic-ray modulations (depressions), to be discussed further in Sect. 7.2, which commence in the vicinity of the interfaces and extend through the high-speed streams. Several sector boundaries/crossings of the heliospheric current sheet (abrupt $\sim 180^\circ$ changes in field azimuth $\phi _B$) occur within this period. One is in the first interaction region, a few hours ahead of the interface. Others are close to the interface in the second interaction region, and near the leading edge of the third interaction region, consistent with the conclusion of Gosling et al. (1978) that sector boundaries lie ahead of the interface.

**Fig. 19:**
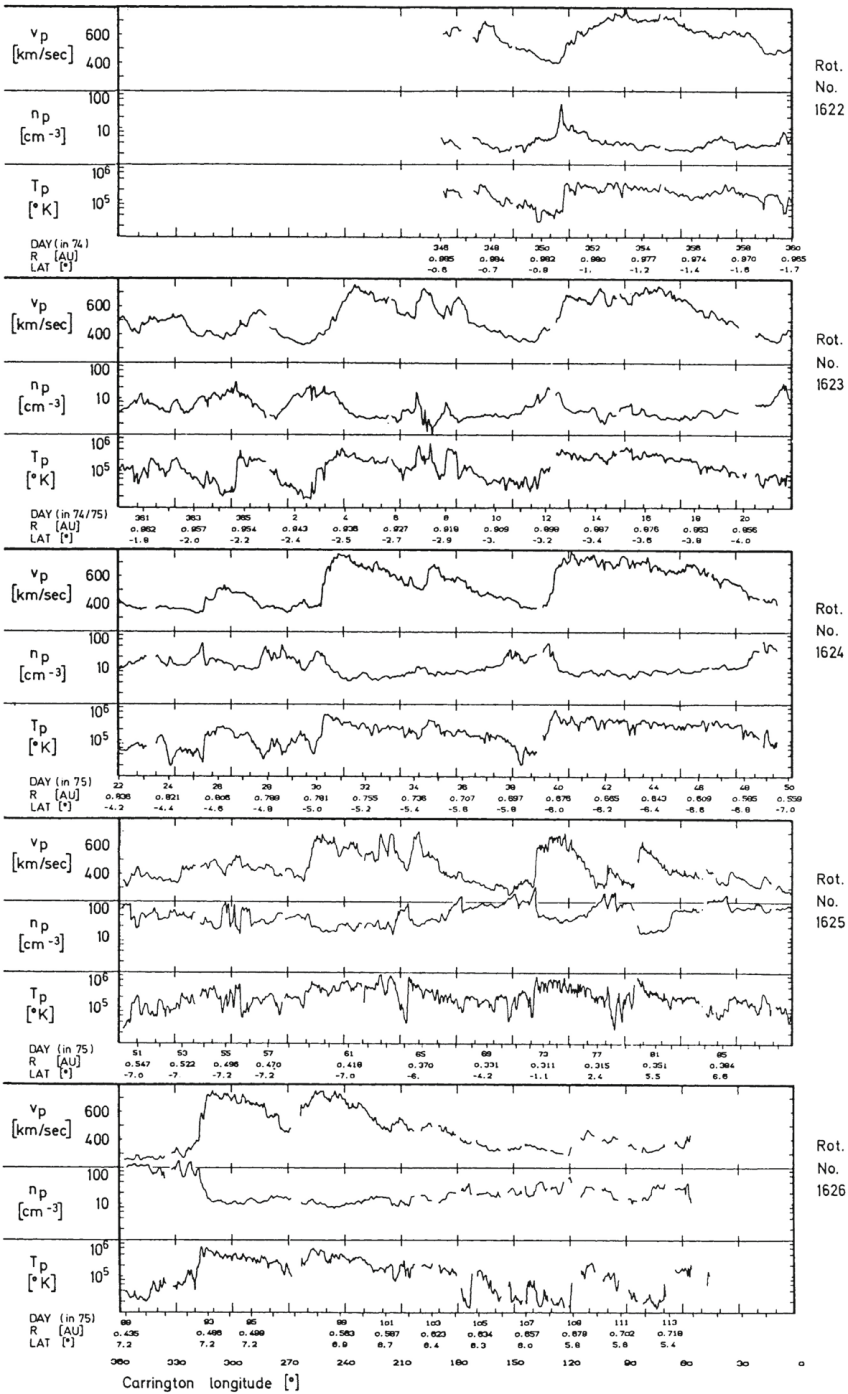
One-hour averages of the solar wind proton speed, density and radial temperature versus Carrington longitude measured by Helios 1 between December 12, 1974 and April 24, 1975. The time, heliocentric distance and heliographic latitude are indicated on the horizontal axis. Speed gradients at the high-speed stream leading edges tend to steepen as the spacecraft moves sunward from $\sim 1$ AU at the beginning of this period to $\sim 0.3$ AU in the fourth panel, before moving back to $\sim 0.7$ AU at the end of this interval. Image reproduced with permission from Schwenn (1990), copyright by Springer

## Observations of stream interaction regions inside 1 AU

Comprehensive observations of stream interaction regions in the inner heliosphere were made by the Helios 1 and 2 spacecraft, which were placed into heliocentric orbits with perihelia of $\sim 0.3$ AU and aphelia of $\sim 1$ AU. Helios 1 was launched on December 10, 1974 with the end of mission occurring on February 18, 1985. Thus, Helios 1 observed the inner heliosphere for over 10 years, extending from the solar minimum between solar cycles 20 and 21 to the minimum between cycles 21 and 22. Helios 2 was launched on January 15, 1976; the mission ended nearly four years later, on December 23, 1979. Results from the Helios missions are extensively reviewed in the two volume “Physics of the Inner Heliosphere” edited by R. Schwenn and E. Marsch (Schwenn and Marsch 1990a, b). Of particular relevance here are the chapters on the large scale structure of the interplanetary medium (Schwenn 1990) and the interplanetary field (Mariani and Neubauer 1990). Chapter 7 of Burlaga (1995) also focuses on Helios observations of corotating streams and interaction regions.

**Fig. 20:**
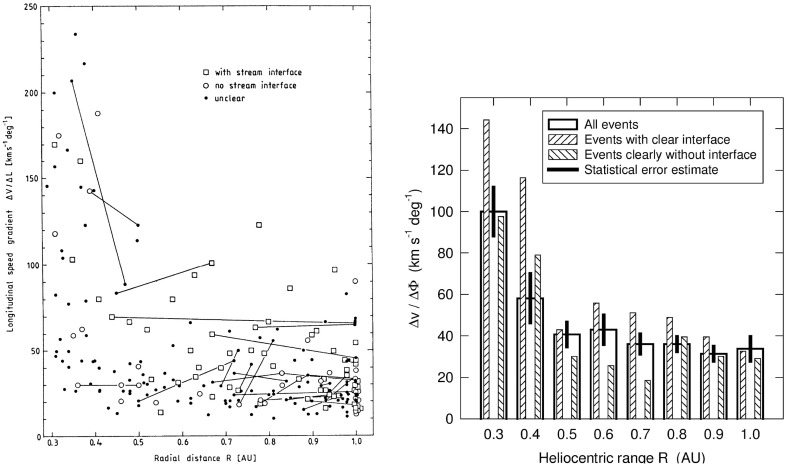
Left: Longitudinal bulk speed gradients at the leading edges of high-speed streams at 0.29–1 AU with “amplitudes” of $\ge 200$ km s$^{-1}$ observed by the Helios spacecraft together with additional observations at 1 AU from IMP 7/8. Stream leading edges observed by two spacecraft are connected. The symbols indicate whether the stream interface is a sharp discontinuity (“with stream interface”). Right: Average longitudinal speed gradients at high-speed stream leading edges between 0.29 and 1 AU. Images reproduced with permission from [left] Schwenn (1990); and [right] Balogh et al. (1999) (adapted from Schwenn 1990), copyright by Springer

As discussed above, one topic of debate before the Helios mission was whether the velocity shears associated with stream interactions steepen or relax between the Sun and 1 AU. Figure 19 from Schwenn (1990) shows the solar wind speed, density, and radial temperature plotted versus Carrington (solar) longitude measured by Helios 1 between 12 December, 1974 and 25 April, 1975 as the spacecraft moved from $\sim 1$ AU in to $\sim 0.3$ AU (in the fourth panel) and returned to $\sim 0.7$ AU at the end of this interval. Inspection of this figure suggests that the speed gradients at the leading edges of the high-speed streams tend to steepen closer to the Sun. This is shown more clearly in the left-hand panel of Fig. 20, which illustrates the longitudinal velocity gradients at the leading edges of a large sample of streams observed by the Helios spacecraft in the inner heliosphere or by IMP 7 or 8 at 1 AU, expressed in km s$^{-1}$ deg$^{-1}$. It is evident that the gradients are steepest nearest to the Sun and tend to flatten out within $\sim 0.5$ AU from the Sun. The right-hand panel of Fig. 20 summarizes these results, showing the average longitudinal speed gradients for streams at different heliocentric distances in bins of 0.1 AU width. Again, these are steepest within $\sim 0.5$ AU and more constant beyond this distance. The longitudinal gradients appear to be larger at a given heliocentric distance for streams “with clear” (discontinuous) interfaces than for those where the interface is less well-defined (“without”). (Notwithstanding these results, Richter and Luttrell 1986 came to the opposite conclusion, that the speed gradient increases with heliocentric distance, based on superposed epoch analysis of a subset of interaction regions at 0.3–0.4 AU and 0.9–1.0 AU, though they acknowledged that this conflicted with earlier work examining individual events by Schwenn et al. 1981.)

Figure 19 also illustrates the evolution of the high-speed stream structure as the prominent streams evident in the first three panels evolve into a more complex structure in panel 4 when close to the Sun, A more prominent stream then emerges near the beginning of the bottom panel. These observations suggest that the stream structure may be more complex close to the Sun, though with observations from just one Helios spacecraft, it can be difficult to separate spatial and temporal variations. Thus, Fig. 21 from Schwenn (1990) shows observations of the solar wind speed at both Helios 1 (dashed line) and 2 (solid line) plotted versus Carrington longitude (to remove the difference in spacecraft longitude) during Carrington rotation 1639 in early 1976. During the first half of this interval, the solar wind speed profiles at both spacecraft are similar (this includes a period when the spacecraft were aligned radially), but they become more structured and differ considerably during the last third of the period when Helios 2 was at $\sim 0.5$ AU, $\sim 7^{\circ }$ south, while Helios 1 was at $\sim 0.3$ AU and ranged from $\sim 2^\circ$ south to $\sim 7^\circ$ north. The differences are interpreted as evidence of considerable latitudinal structure in high-speed streams in the inner heliosphere.

**Fig. 21:**
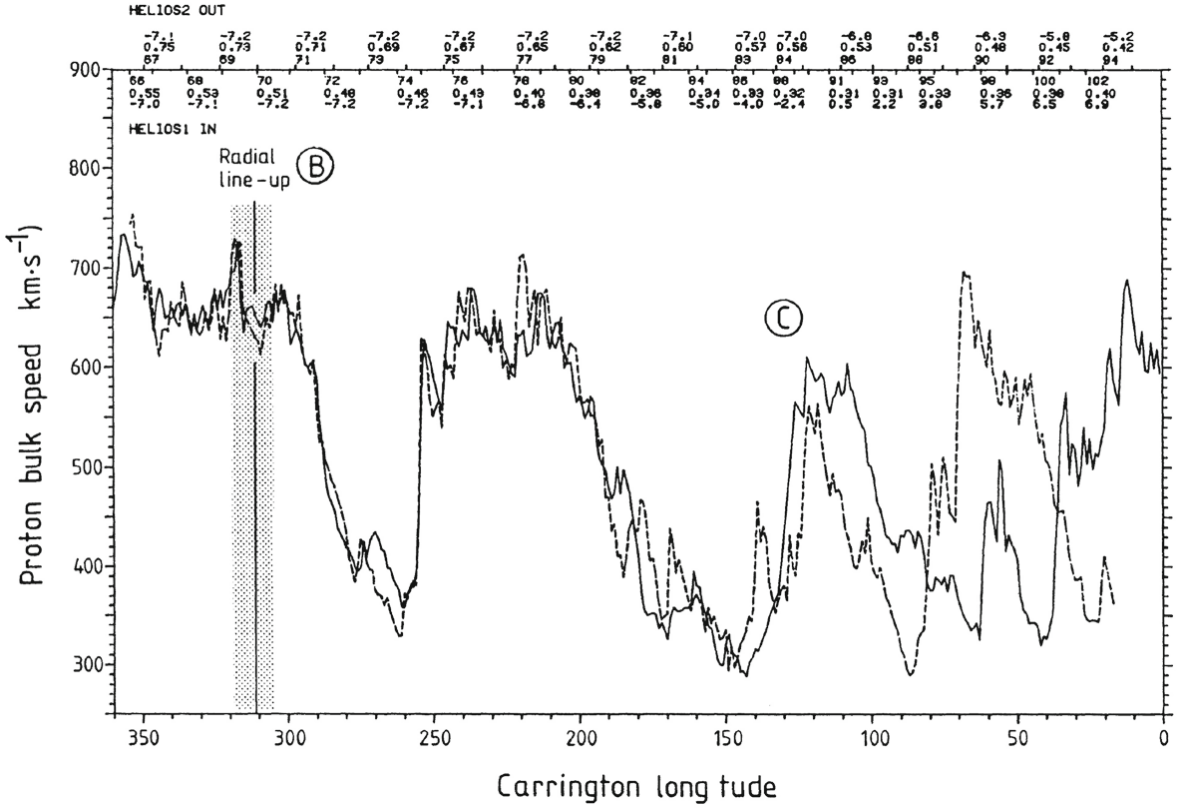
The solar wind speed at the Helios 1 (dashed line) and 2 (solid line) versus Carrington longitude during Carrington rotation 1639 showing the difference in stream structure as the spacecraft separate in latitude (after ‘C’) in the second half of the rotation. ‘B’ indicates when the spacecraft were radially aligned. Image reproduced with permission from Schwenn (1990), copyright by Springer

**Fig. 22:**
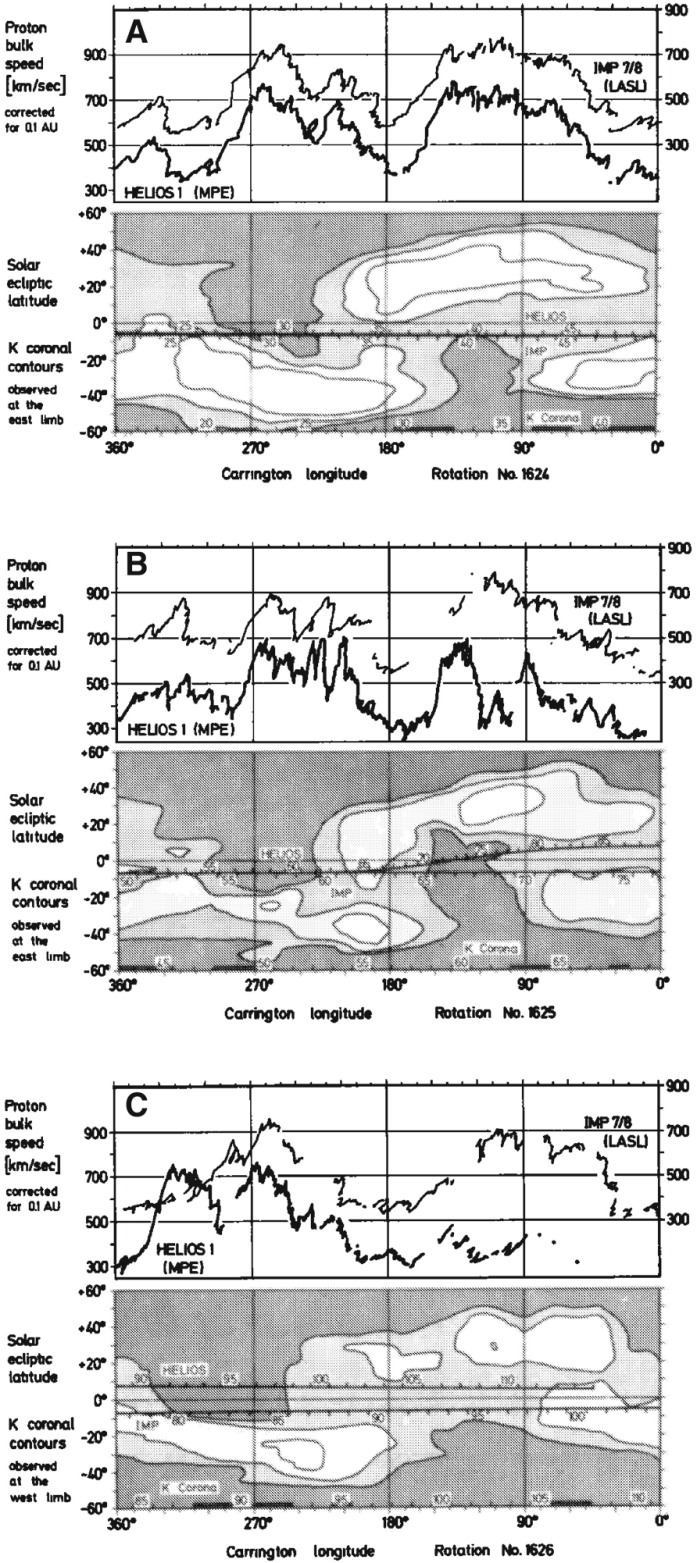
Comparison of solar wind speeds measured at Helios 1 and IMP 7/8 with K corona contours (darker shaded regions indicate coronal holes, lighter regions, the streamer belt) for Carrington rotations 1624 (A), 1625 (B) and 1626 (C). After the middle of B, the spacecraft become separated in latitude (the spacecraft tracks in latitude are indicated on the K corona plots) and the speed profiles then differ considerably. Image reproduced with permission from Schwenn et al. (1978), copyright by AGU

Other evidence for latitudinal structure was presented by Schwenn et al. (1978). In each of the panels in Fig. 22, the top half shows the solar wind speeds from IMP 7/8 in Earth orbit and at Helios 1. As before, the observations are plotted versus Carrington rotation to remove the longitudinal separation between the spacecraft. The bottom half of each panel shows the coronal hole configuration as inferred from K coronal observations at the east limb made at Mauna Loa, Hawaii (Hansen et al. 1976) indicating the bright “streamer belt” threading around the equatorial regions and the darker coronal hole regions. The latitudes of the spacecraft are superposed on the K corona maps. Until the middle of panel B, the spacecraft were at similar latitudes and observed generally similar solar wind speed profiles. After this time, the spacecraft became separated in latitude, and the profiles show larger differences. In particular, the right-hand side of panel C shows that the IMPs observed a high-speed stream but Helios 1 did not. This is consistent with Helios 1 being north of the IMPs so that it did not encounter the flow from the southern coronal hole in which the IMPs were immersed. Such observations again suggest that the boundaries of high-speed streams near the Sun are rather sharp in latitude, as discussed by Schwenn et al. (1981). In particular, they concluded that if two spacecraft are separated by more than $5^\circ$ in latitude, they have only a relatively small chance of encountering similar streams, and that large differences in solar wind speed (up to 250 km s$^{-1}$) can occur even for small latitudinal separations of $\approx 1.5^\circ$.

**Fig. 23:**
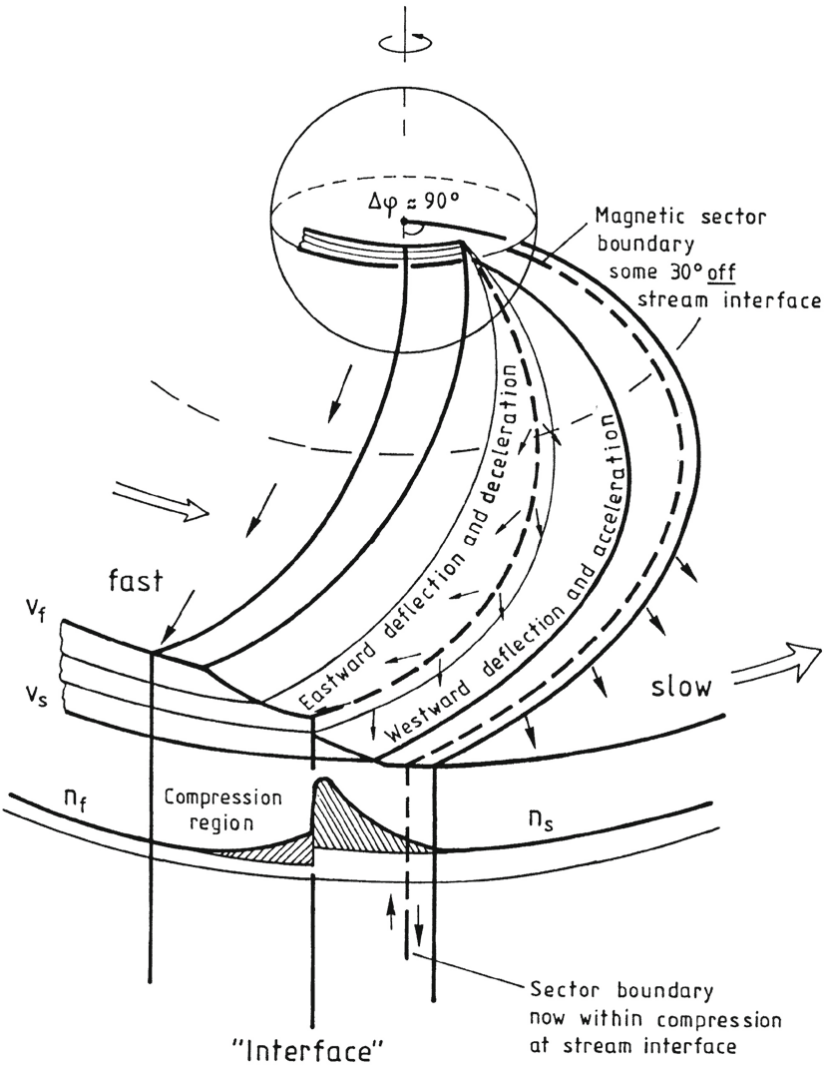
Idealized view of a stream interaction region and its evolution in the inner heliosphere based on Helios observations. Image reproduced with permission from Schwenn (1990), copyright by Springer

**Fig. 24:**
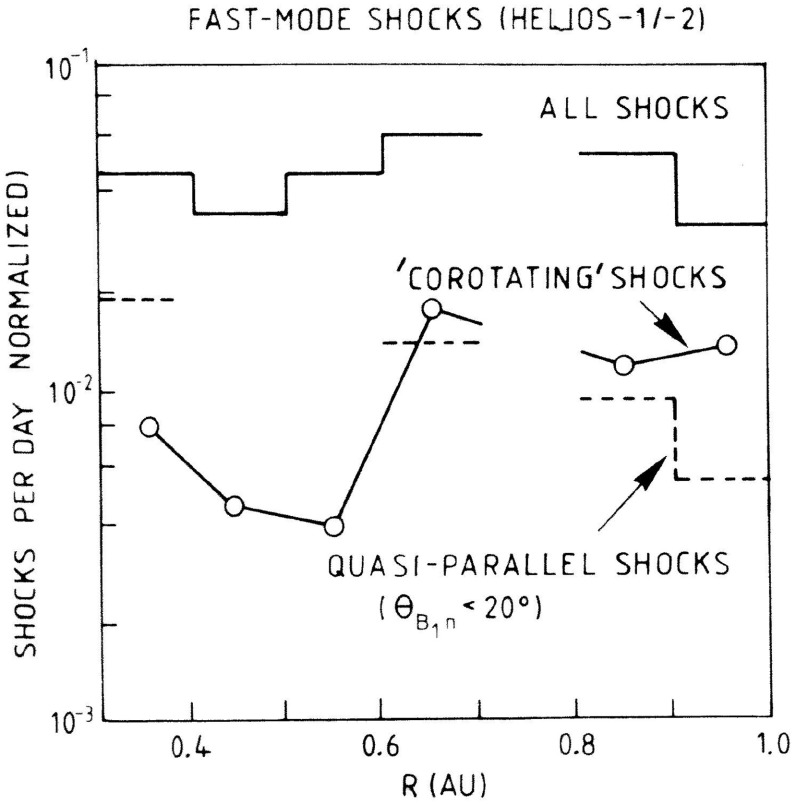
Normalized rate versus radial distance of “fast mode shocks” identified in Helios 1 and 2 measurements from December 1974 to December 1980. The rate of corotating shocks, of interest here, appears to fall off within $\sim 0.5$ AU from the Sun. Image reproduced with permission from Richter et al. (1985), copyright by AGU

Figure 23 shows an idealized view of a stream interaction region in the inner heliosphere based on Helios observations (Schwenn 1990) that indicates the change from a “rectangular” speed profile at the Sun to a more gradual speed increase in the solar wind. One point that it illustrates is the tendency for the magnetic sector boundary to be well ahead of the interface near the Sun, but to move closer to, and become entrained within the compression region with increasing distance from the Sun.

Shocks were very occasionally associated with the interaction regions observed by Helios. For example, Schwenn (1990) notes that around 25 interaction regions in the Helios data set showed evidence of a possible forward shock, though on further investigation, only around six cases were found likely to be fully-developed shocks. Fast reverse shocks were found at “less than ten” interaction regions. The closest shock to the Sun was observed at 0.63 AU. Schwenn (1990) does not indicate the total number of interaction regions examined, so the fraction with shocks is difficult to estimate. Making a crude estimate based on say ten years of observations and two interaction regions/rotation (ignoring any solar cycle variation, that there may be more or fewer interaction regions/rotation, and many other factors) suggests around 270 interaction regions, indicating that the fraction with shocks may be only around 2% (forward shocks) to $<4\%$ (reverse shocks). This appears to be less than the $\sim 17\%$ and $\sim 6\%$, respectively, reported by Jian et al. (2006) for interaction regions at 1 AU, suggesting that the fraction of interaction regions with shocks may be lower in the inner heliosphere. In an earlier study, Richter et al. (1985), using a subset of Helios 1 and 2 observations from December 1974 to December 1980, inferred the radial dependence of the “fast mode” corotating shock rate shown in Fig. 24 (it is not specifically stated whether or not this includes both forward and reverse fast shocks). The rates are corrected for the time spent by Helios at different radial distances. The results indicate that corotating shocks are less frequent within 0.5 AU of the Sun (at a rate of around one shock every 200 days) than at 0.5–1 AU (around one shock per 100 days or less). There are also shocks in this study inside the distance of the closest shock to the Sun (at 0.63 AU) reported by Schwenn (1990) suggesting that the identification criteria are not completely consistent between the studies. Shocks at the Helios (and other) spacecraft are also included in the new shock data base developed at the University of Helsinki (http://ipshocks.fi/). For example, only six fast reverse shocks, all apparently at the trailing edges of interaction regions from inspection of the data figures in the data base, are identified in the Helios 1 and 2 data, again indicative of the low rate of corotating shocks in the inner heliosphere.

**Fig. 25:**
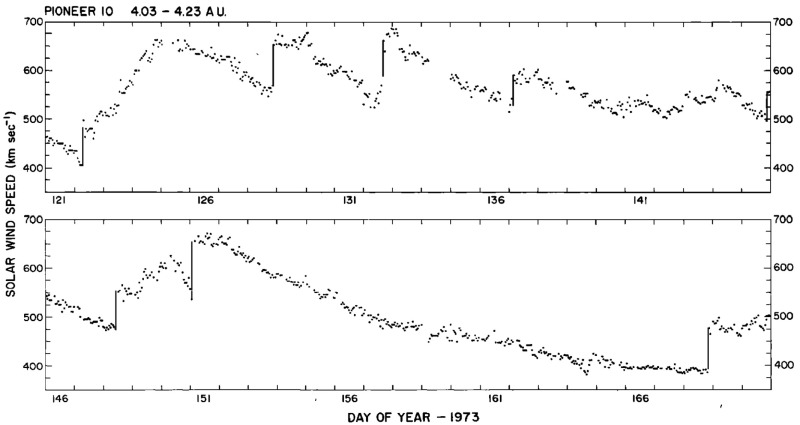
The solar wind speed at Pioneer 10 at $\sim 4$ AU during a 50 day period in 1973, showing the frequent steepening of stream leading edges into abrupt jumps in speed, typically associated with shocks. Image reproduced with permission from Hundhausen and Gosling (1976), copyright by AGU

## Observations of stream interaction regions beyond 1 AU

The first detailed observations of stream interaction regions beyond the orbit of Earth were made by the Pioneer 10 and 11 spacecraft, launched towards Jupiter on March 2, 1972 and April 6, 1973, respectively. Figure 25 shows a 50 day interval of solar wind speed data from Pioneer 10 as it moved from 4.03 to 4.23 AU (Hundhausen and Gosling 1976). The vertical lines indicate abrupt jumps in speed at the leading edges of many of the streams present. In particular, it was found that interaction region boundaries tend to steepen to form a forward fast shock at the leading edge of the interaction region and a reverse shock, propagating sunward in the solar wind frame, at the trailing edge, as previously discussed in relation to Fig. 17. Examples of shocks observed by Pioneer 10 are shown in Fig. 26 from Smith and Wolfe (1976). The Pioneer 10 and 11 observations demonstrated that such shocks tend to form beyond 2 AU (e.g., Gosling et al. 1976; Hundhausen and Gosling 1976; Smith and Wolfe 1976) and that by 3–5 AU, over 90% of interaction regions were found to have forward shocks and $\sim 75\%$ reverse shocks, far higher rates than found at 1 AU or at Helios, as noted above,

**Fig. 26:**
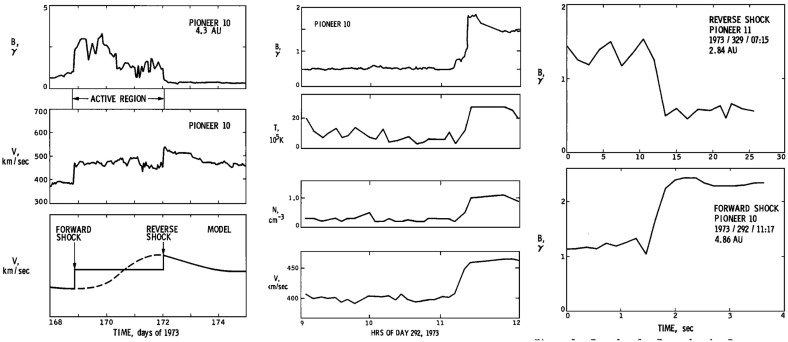
Left: An interaction region (termed an “active region”) observed by Pioneer 10 at 4.3 AU, showing a fast forward shock at the leading edge and a fast reverse shock at the trailing edge. The bottom panel indicates how the gradual speed transition observed at 1 AU is replaced by a region of small speed gradient bounded by the pair of shocks. Center: High resolution (5-min averaged plasma and 1-min averaged magnetic field) data showing a fast forward shock. Right: High resolution observations of a forward shock (1.5 s averages) and reverse shock (0.1875 s averages). Image reproduced with permission from Smith and Wolfe (1976), copyright by AGU

**Fig. 27:**
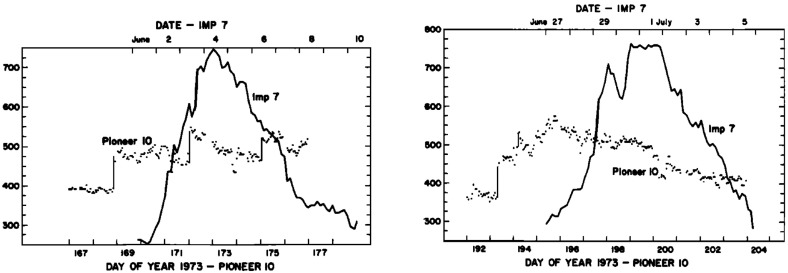
Two high-speed streams observed at IMP 7 and Pioneer 10 at 4 AU, showing the steepening of the stream leading edge, including the formation of abrupt speed jumps associated with shocks, and the reduction in the peak speed of the stream. Image reproduced with permission from Gosling et al. (1976), copyright by AGU

**Fig. 28:**
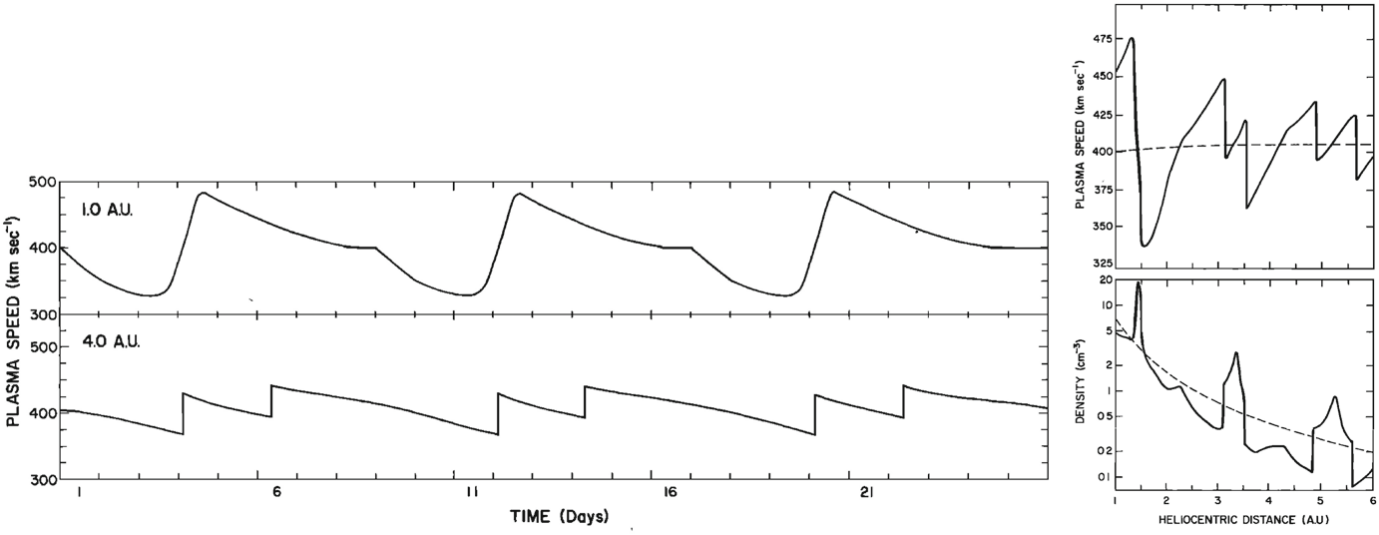
Left: Solar wind speed versus time introduced at 1 AU into the model of Hundhausen and Gosling (1976) (upper panel) and the profile predicted by the model at 4 AU (bottom panel), showing the development of shock pairs on the stream leading edges and erosion of the peak speeds similar to the observations in Fig. 27. Right: A snapshot of the modeled speed and density variation with heliocentric distance showing the three streams and the steepening of the interaction regions to form forward–reverse shock pairs. Image reproduced with permission from Hundhausen and Gosling (1976), copyright by AGU

Figure 27 compares the solar wind speed profiles at IMP 7 at the Earth and at Pioneer 10 at 4 AU for two streams—the profiles have been shifted to allow for the Archimedean spiral configuration between the two spacecraft. The steeping of the leading edge of the streams in the outer heliosphere is evident, including the development of steps associated with shocks. Other clear differences are that the stream peak speeds are reduced and streams are less structured than at 1 AU. The reduction in peak speed is consistent with the expansion of the interaction region into the fast stream, decelerating and “eroding” the high-speed plasma. Hundhausen and Gosling (1976) showed that this stream development was qualitatively consistent with the predictions of a hydrodynamic, time-dependent, spherically symmetric solar wind model in which turbulent dissipation is negligible except at shocks (Hundhausen 1973b, a) as illustrated in Fig. 28, including the formation of pairs of abrupt jumps resembling forward and reverse shocks. The observations and model results were inconsistent with competing views that the stream structure would decay beyond 1 AU, for example by turbulent dissipation (e.g., Jokipii and Davis 1969; Davis 1972).

**Fig. 29:**
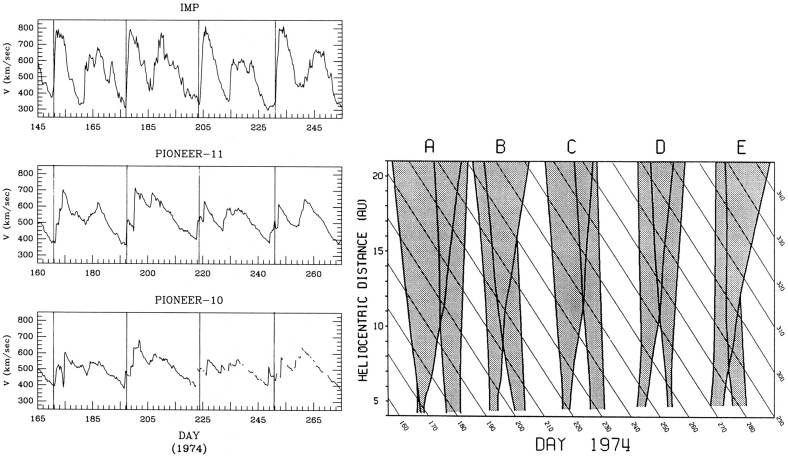
Left: Solar wind speed observations at IMP 7/8, Pioneer 11 at 4.6 AU and Pioneer 10 at 5.8 AU during a period in 1974 illustrating the merging of pairs of streams in the outer heliosphere (“period doubling”). Right: 1-D MHD modeling of the merging of the interaction regions (shaded) and shocks (black curves) during this period to form corotating merged interaction regions beyond $\sim 7$ AU. Note that the slower stream merges with the fast stream, not vice versa, because it is wider and the faster stream is more rapidly eroded by the expanding interaction region. Images reproduced with permission from [left] Burlaga et al. (1990) and [right] Whang and Burlaga (1990), copyright by AGU

Figure 29 compares, in the left-hand panel, the solar wind speed profiles at (top to bottom) IMP 7/8, Pioneer 11 at 4.6 AU, and Pioneer 10 at 5.8 AU, during an interval in 1974 and illustrates another interesting feature of solar wind stream development in the other heliosphere, in addition to the formation of shock pairs at the stream leading edges and the reductions in stream speeds. The stream structure becomes simpler, with pairs of streams bounded by the vertical lines at 1 AU tending to merge at larger radial distances (the observations have been aligned by assuming a spiral stream configuration). This “period doubling” process has been discussed by Burlaga et al. (1990). An interesting feature of the observations in Fig. 29 is that one of the two corotating streams is faster than the other, but the merging does not occur when the faster stream overtakes the preceding slower stream, as might be expected. Rather, the slower stream merges with the preceding fast stream. Among the contributing factors are that the slower stream is wider than the fast stream, and the interaction region tends to be stronger ahead of the fast stream, causing the fast plasma flows there to be eroded more rapidly. This process is modeled using a 1-D MHD code in the right-hand panel of Fig. 29 from Whang and Burlaga (1990), where the interaction regions are shaded and the black curves are the forward and reverse shocks. Each successive pair of interaction regions merges to form a corotating “merged interaction region” at $\sim 7$ to 10 AU. Thus, the stream structure evolves from two streams and interaction regions/solar rotation at 1 AU to one merged interaction region/rotation beyond $\sim 7$ AU. Burlaga (1995) notes that “the asymmetries in the widths and heights of the recurrent streams are crucial to the formation of corotating merged interaction regions and period doubling” and that small perturbations in these and other parameters “can produce qualitative changes in the structure of the outer heliosphere”. In particular, simple models including two similar streams may not be reliable predictors of how the outer heliosphere stream structure evolves.

**Fig. 30:**
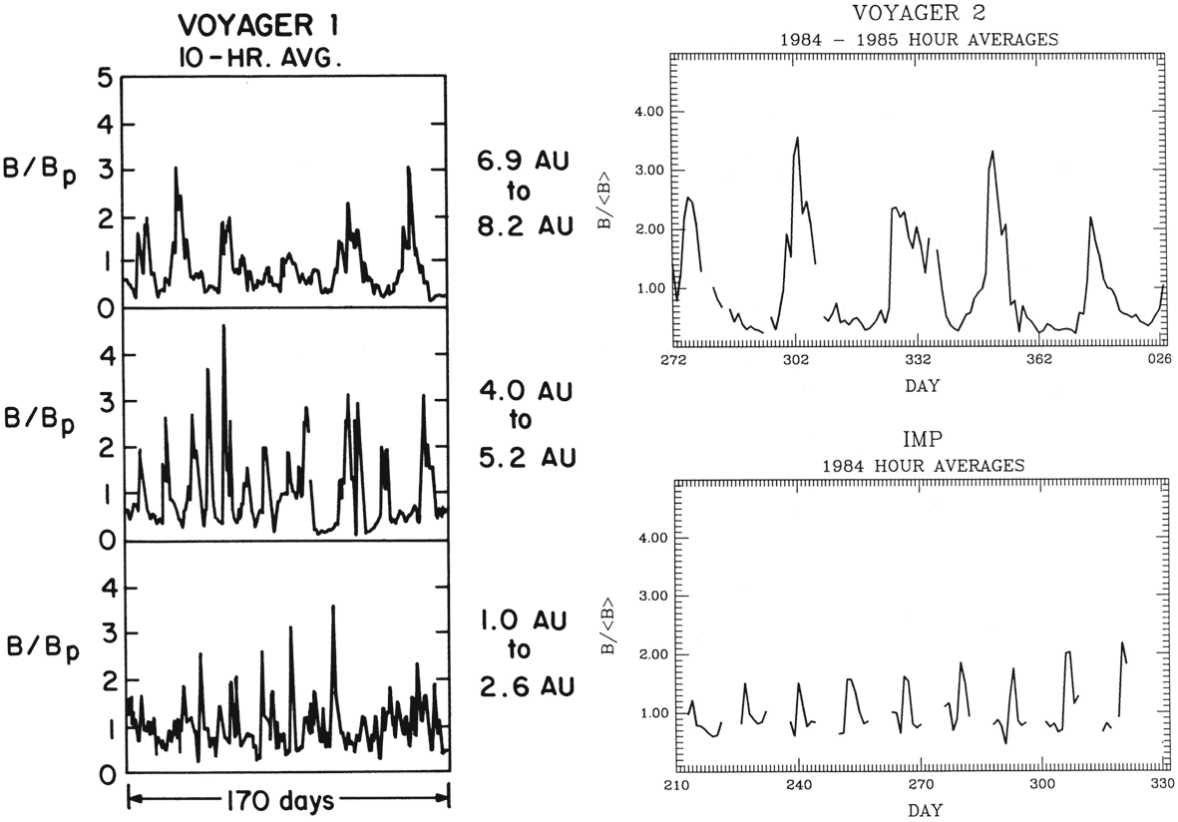
Left: Formation of merged interaction regions (indicated by magnetic field enhancements above the nominal Parker spiral value $B_p$) with increasing distance from the Sun. Right: Illustration of “period doubling” between IMP 8 at 1 AU and Voyager 2 at 15.2–16.2 AU. Images reproduced with permission from [left] Burlaga et al. (1984), copyright by AGU; and [right] Burlaga (1995) (after Burlaga 1988), copyright by OUP

The Voyager 1 and 2 missions (launched on September 5, 1977 and August 20, 1977, respectively; https://voyager.jpl.nasa.gov/) also observed interaction regions in the outer heliosphere en route to the outer planets and beyond. The left-hand panel of Fig. 30 from Burlaga et al. (1984) shows Voyager 1 magnetometer observations for three 170-day ($\sim 6$-solar-rotation) periods at different heliocentric distances showing the simplification of the interaction region structure (indicated by the enhancements in field strength relative to the nominal Parker spiral value $B_p$) with increasing distance. In particular, the merged interaction regions beyond 6.9 AU occur approximately once per solar rotation, notwithstanding the more complex structures observed closer to the Sun. The right-hand panel of Fig. 30 shows similar observations at IMP 8 and at Voyager 2 when at 15.2–16.2 AU, clearly illustrating the period doubling and formation of merged interaction regions recurring at the solar rotation period in the outer heliosphere. (The solar wind transit speed between 1 AU and Voyager 2 has been taken into account when comparing the intervals at the two locations.)

Figure 31 from Burlaga et al. (1984) shows in the left-hand panel the times of forward and reverse shocks observed at Voyager 1 after launch in a stacked solar rotation format (with time running downwards), illustrating the tendency for shock pairs to occur more frequently with increasing heliocentric distance and to recur at the solar rotation period. Note also that the stream interfaces (dots) tend to occur between the shocks, as would be expected. The right-hand panel illustrates the increasing separation between pairs of forward and reverse shocks with heliocentric distance, consistent with expansion of the interaction regions.

**Fig. 31:**
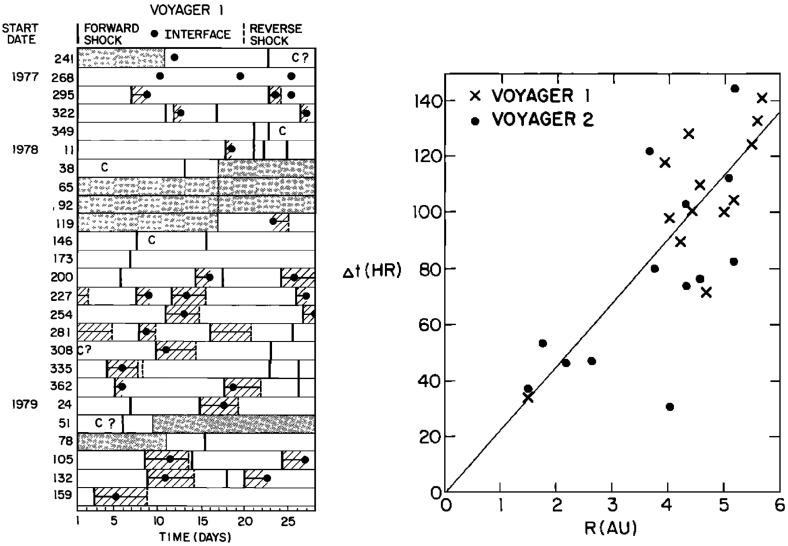
Left: Times of forward and reverse shocks and stream interface crossings observed by Voyager 1 from launch to day 186 of 1979, showing the more frequent formation of shock pairs with increasing heliospheric distance and the tendency for the shock pairs to recur at the solar rotation period. Diagonally hatched intervals indicate interaction regions, shaded intervals indicate data gaps, and ‘C’ denotes a transient “magnetic cloud” (Klein and Burlaga 1982). Right: The time separation between forward and reverse shocks associated with the same interaction region increases with heliocentric distance, consistent with expansion of the interaction region. Images reproduced with permission from [left] Klein and Burlaga (1982) and [right] Burlaga et al. (1984), copyright by AGU

**Fig. 32:**
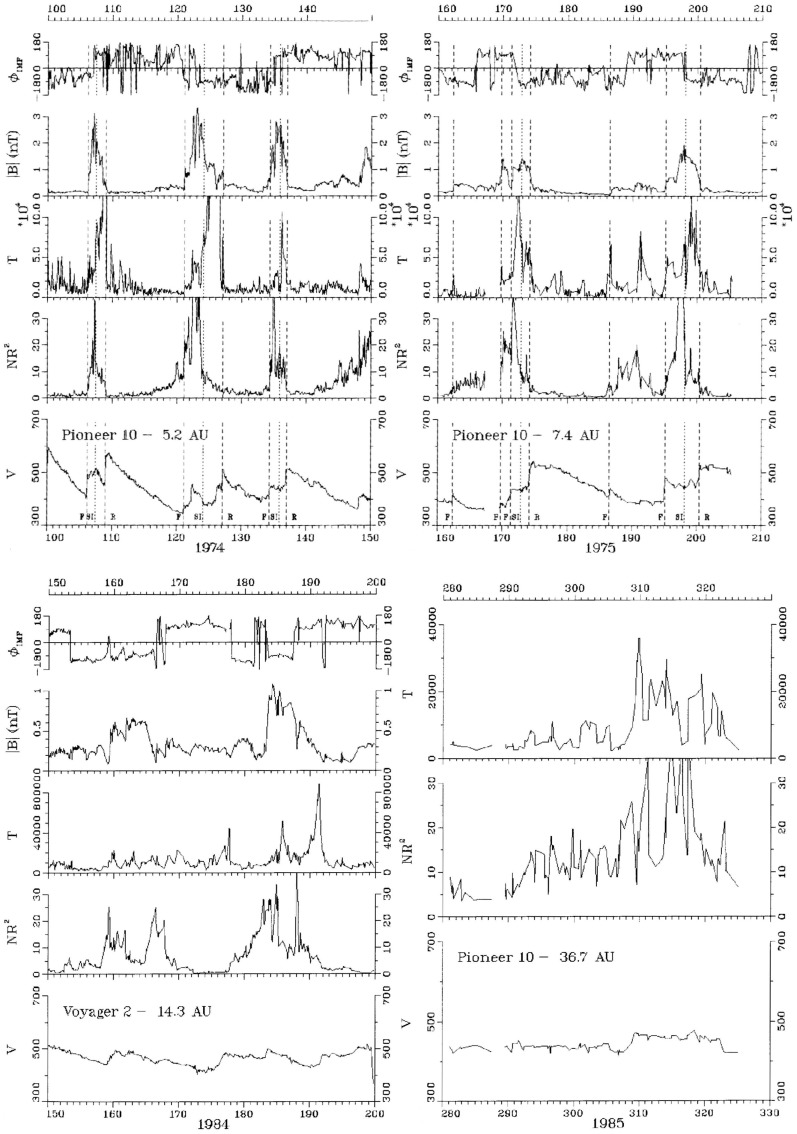
Top panels: Examples of stream interaction regions during 50 day (approximately 2 solar rotation interval) periods observed by Pioneer 11 at 5.2 AU (left) and 7.4 AU (right). Parameters shown are the magnetic field azimuthal angle and intensity, and solar wind temperature, density (multiplied by $R^2$ to account for radial expansion), and speed. Forward and reverse shocks are indicated by vertical dashed lines, and stream interfaces by dotted lines. Note the stream structure is simpler at the larger distance and includes two merged interaction regions. Bottom panels: Similar observations from Voyager 2 at 14.3 AU, showing two corotating “pressure waves”, and from Pioneer 10 at 36.7 AU near the solar equator, showing irregular, non-periodic density and temperature structures. Images reproduced with permission from Gazis et al. (1999), copyright by Kluwer

**Fig. 33:**
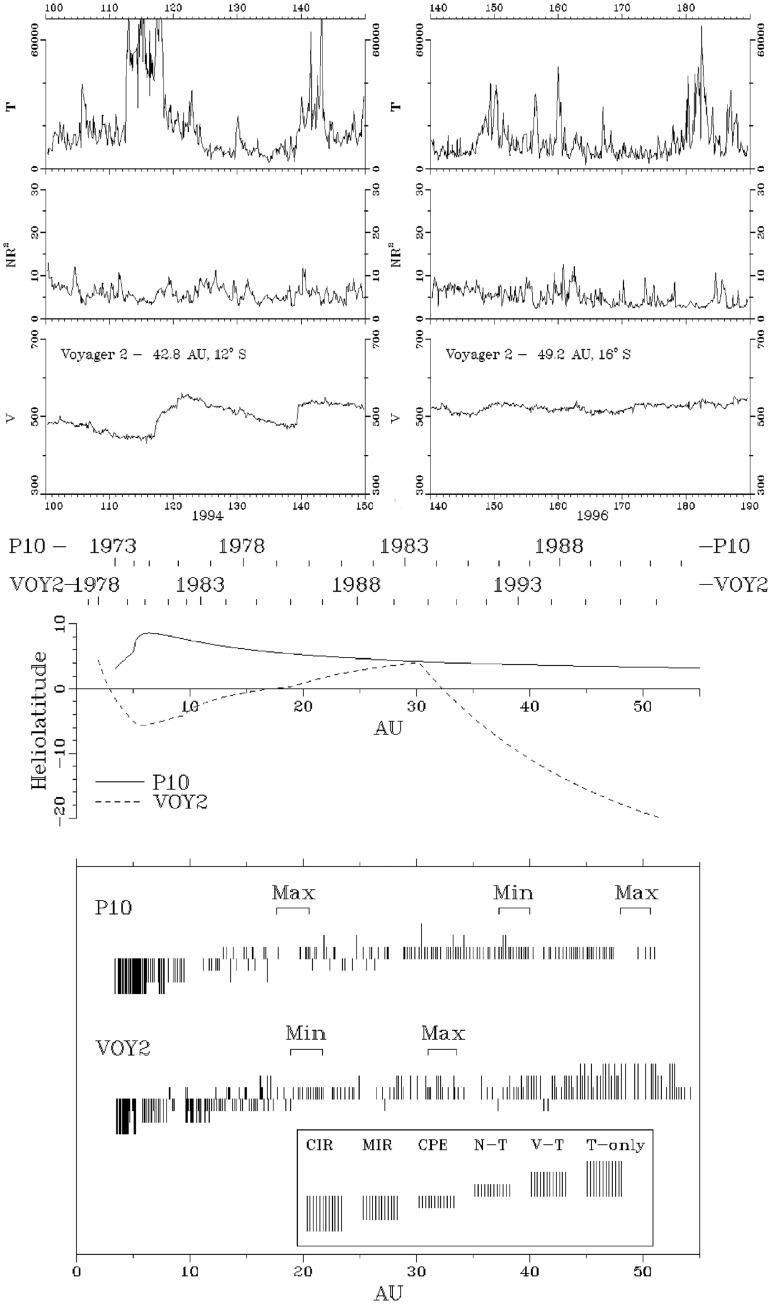
Top panel: Similar Voyager 2 solar wind plasma observations at 42.8 AU, $12^\circ$S (left) and 49.2 AU, $16^\circ$S. In contrast to the lack of periodic structures seen at Pioneer 10 near the ecliptic at a similar distance in the bottom-right panel of Fig. 32, Voyager 2 observed quasi-periodic speed and temperature enhancements at higher latitudes. By 49.2 AU, the quasi-periodic structures are predominantly present in the temperature. Bottom panel: Summary of the types of interacting structures observed by Pioneer 10 and Voyager 2 as a function of heliocentric distance; the spacecraft radial distance and latitude are also shown. “Max.” and “Min.” indicate the level of solar activity at the time of the observations for each spacecraft. The evolution of interaction regions with distance illustrated in Fig. 32 and the top panel of this figure is evident. Images reproduced with permission from Gazis et al. (1999), copyright by Kluwer

Figures 32 and 33 from Gazis et al. (1999) illustrate the evolution of interaction regions from 5.2 AU to nearly 50 AU. Each data figure shows a 50 day (approximately 2 solar rotation period) sample interval at a certain heliocentric distance and illustrates the magnetic field azimithal angle and intensity, proton temperature, the density multiplied by $R^2$ and solar wind speed. In the Pioneer 10 observations at 5.2 AU in the top left panel of Fig. 32, three interaction regions can be identified, typically bounded by forward–reverse shock pairs as discussed above. Stream interfaces within the interaction regions may also be identified, indicated by decreases in density and increases in temperature. The top right panel shows Pioneer 10 observations at 7.4 AU. Here, as discussed above, the interaction regions are wider and may be formed from mergers of interaction regions but the pattern of dense, lower temperature, lower speed plasma ahead of the interaction region followed by less dense, higher temperature, higher speed plasma following the interaction region is still maintained. In the Voyager 2 observations at 14.3 AU in the lower left panel of Fig. 32, the stream structure is highly eroded and shocks are not present. The main features are two corotating regions of stronger magnetic field and plasma density, i.e., pressure enhancements (Burlaga 1983) that tend to recur at the solar rotation period. The bottom right panel of Fig. 32 shows observations from Pioneer 10 at 36.7 AU that show a broad, irregular density and temperature enhancement unrelated to shocks and associated with only a slight increase in solar wind speed. There is little evidence of periodicity, which is typical of observations beyond 15 AU in the vicinity of the solar equator. In contrast, the plasma observations in the top left-hand panel of Fig. 33 from Voyager 2 at a similar distance but at $12^\circ$ south, still show evidence of quasi-periodic enhancements in the solar wind speed and temperature, though only small variations in density (Burlaga et al. 1997). However, when Voyager 2 reached 49.2 AU, $16^\circ$ (top-right panel), only periodic variations in the proton temperature remained.

The lower panels of Fig. 33 indicate the heliolatitudes of Pioneer 10 and Voyager 2 as a function of radial distance and the different types of interacting structures observed as they moved out through the heliosphere. Between 2–8 AU, corotating interaction regions were most prominent, with merged interaction regions starting to replace corotating interaction regions between 5–8 AU and becoming most common at 8–12 AU. At 10–12 AU, shocks decline and merged interaction regions are replaced by corotating pressure enhancements, which are common out to 15–20 AU. Beyond 30 AU, the spacecraft became significantly separated in latitude, and observe different structures, including variations in speed and/or temperature. Since the topic of this review is “stream interaction regions”, and the fundamental role of speed gradients and streams in producing interaction regions is evidently reduced much beyond $\sim 10$ AU, we will not consider the evolution of structures in the distant heliosphere further here.

**Fig. 34:**
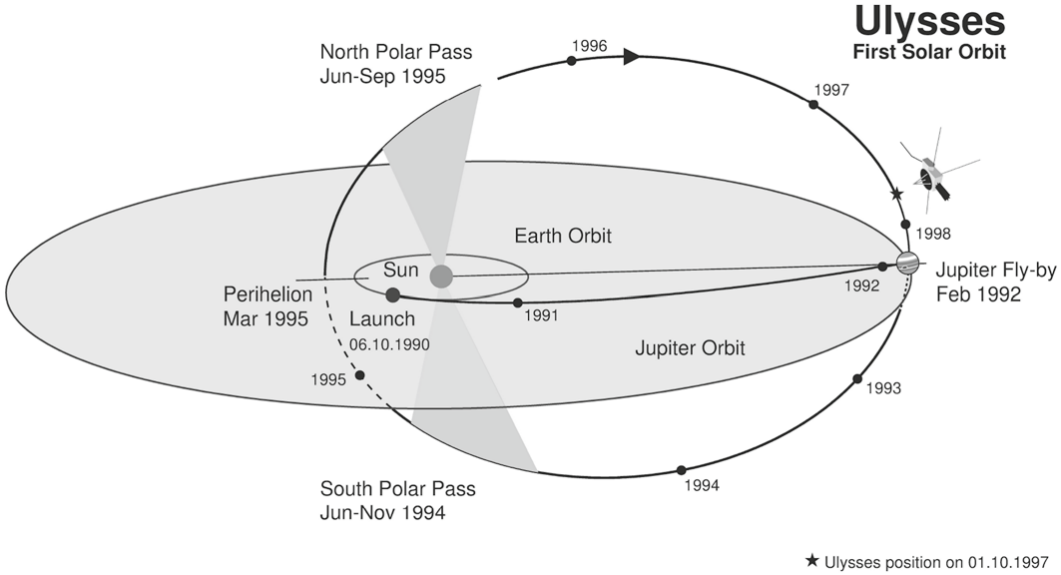
Ulysses spacecraft trajectory from launch to Jovian swing-by and first orbit of the Sun. Image by European Space Agency

## Observations of stream interaction regions by Ulysses: the three dimensional aspect

Another major advance in understanding stream interaction regions was provided by the Ulysses mission (http://sci.esa.int/ulysses/). Ulysses was launched on October 6, 1990, and, following a polar swing-by of Jupiter, was placed into a high inclination ($79^\circ$) heliocentric orbit, thereby extending our view of the heliosphere to high latitudes (Fig. 34). Note that Ulysses was close to the ecliptic only near the orbit of Jupiter at $\sim 5$ AU, and again near 1 AU during pole to pole “fast latitude scans” around perihelion. The first $\sim 6$ year orbit (from aphelion in 1992–1998) occurred predominantly in the solar minimum between solar cycles 22 and 23, the second (1998–2004) encompassed the peak of cycle 23, while the third orbit (incomplete due to mission end on June 30, 2009) occurred during the solar minimum between cycles 23 and 24. Results from Ulysses during the first orbit are summarized in Balogh et al. (2001). In particular, Chapter 3 (Forsyth and Gosling 2001) discusses stream interactions. Kunow et al. (1999) also focus on observations of interaction regions during the first orbit, while Gosling and Pizzo (1999) review the three-dimensional structure of stream interaction regions from a modeling perspective.

**Fig. 35:**
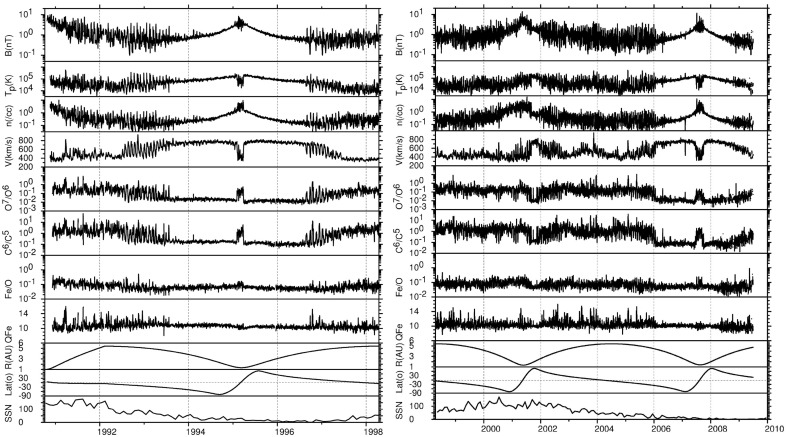
Left: Solar wind observations made during the first orbit of the Ulysses mission, specifically the magnetic field intensity, proton temperature, density and speed, $\hbox {O}^7/\hbox {O}^6$, $\hbox {C}^6/\hbox {C}^5$, and Fe/O ratios and mean Fe charge state. The bottom panels show the spacecraft radial distance and latitude and the sunspot number. Right: Ulysses solar wind observations during the second and (partial) third orbits until the end of the mission. Note the contrast between the variable solar wind flows at all latitudes during the second orbit, around the peak of solar cycle 23, and those during the third orbit at lower activity levels, which more closely resemble the configuration during the first orbit in the left panel

Figure 35 summarizes solar wind observations during the Ulysses mission, specifically the magnetic field intensity, proton temperature, density and speed, $\hbox {O}^7/\hbox {O}^6$, $\hbox {C}^6/\hbox {C}^5$, and Fe/O ratios and mean Fe charge state. The bottom panels show the spacecraft radial distance and latitude and the sunspot number. Considering the first orbit (left panel), following launch near the maximum of solar cycle 22, Ulysses moved away from the Sun at low latitudes, encountered Jupiter in early 1992, and was placed in an orbit that took it first to high southern latitudes while moving closer to the Sun. Maximum latitude of $80.2^\circ$S during the first polar pass occurred on September 13 1994. A fast scan in latitude from south to north then took place, and maximum northern latitude ($80.2^\circ$N) was reached on July 31 1995.

**Fig. 36:**
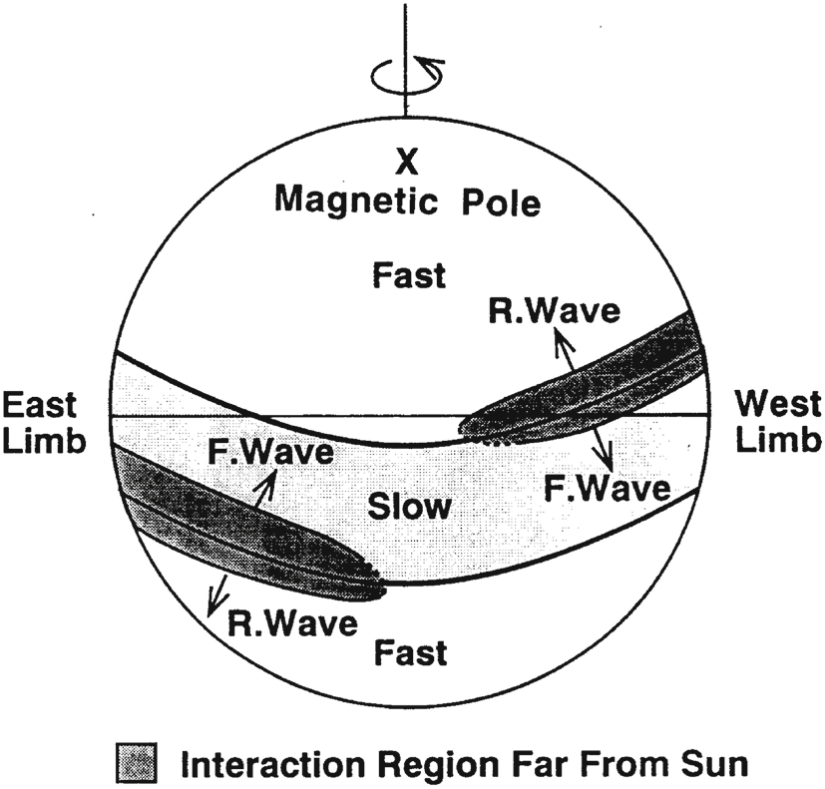
Simple configuration of slow solar wind from a tilted streamer belt and fast flows from higher latitudes showing the development of interaction regions (dark shading) including equatorward-propagating forward waves or shocks and poleward-propagating reverse waves/shocks. Image reproduced with permission from Gosling and Pizzo (1999), copyright by Kluwer

The first orbit of the Sun occurred during the decline of solar cycle 22 and the subsequent solar minimum. Ulysses observed a simple configuration of slow solar wind at low latitudes, and high-speed flows at higher latitudes originating in polar coronal holes, which is characteristic of low solar activity conditions. [The presence of fast solar wind at high latitudes at solar minimum had been previously inferred from interplanetary scintillation measurements (Kakinuma 1977; Kojima and Kakinuma 1990; Rickett and Coles 1991).] At mid-latitudes, alternating streams of fast and slow solar wind are evident. Interaction regions are indicated by the enhancements in the magnetic field intensity, density, and proton temperature, and variations in the solar wind speed, recurring at the solar rotation interval ($\sim 26$ days at Ulysses); examples will be shown in more detail below. Also evident in the left panel of Fig. 35 are the differences in solar wind composition between slow and fast solar wind (e.g., Geiss et al. 1995b; von Steiger et al. 2000; Richardson and Cane 2004; Richardson 2014), in particular lower values of $\hbox {O}^7/\hbox {O}^6$, and $\hbox {C}^6/\hbox {C}^5$ in faster solar wind.

The Ulysses observations clearly demonstrate that the pattern of alternating slow and fast solar wind streams and associated interaction regions observed near the ecliptic discussed above is a manifestation of the large scale, three-dimensional structure of the solar wind. Figure 36 from Gosling and Pizzo (1999) shows a schematic of the interaction of slow, low latitude solar wind associated with a tilted streamer belt (due to the magnetic pole being displaced from the rotational axis) and fast solar wind from high latitudes, producing such a recurring pattern of slow and fast solar wind at low latitudes and forming interaction regions (dark shading) at the leading edges of the faster solar wind; a warped streamer belt would also result in a similar configuration. One point to note, which we will return to, is that in this scenario, the forward waves (or shocks) at the leading edges of the interaction regions ahead of both the northern and southern fast flows are propagating away from the poles while the reverse waves/shocks are propagating poleward.

**Fig. 37:**
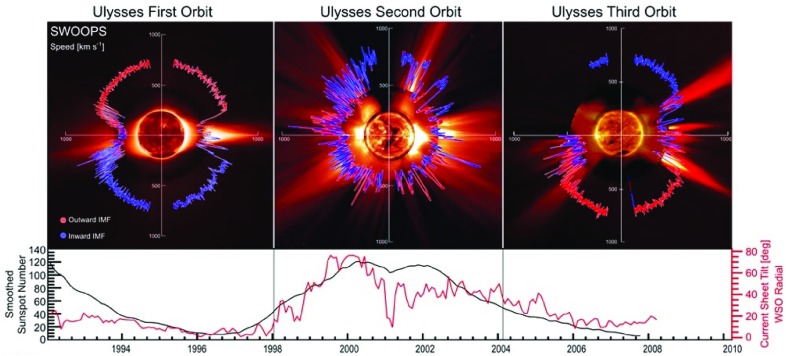
Polar plots of the solar wind speed during Ulysses’ three orbits of the Sun showing fast solar wind at high latitudes, slow solar wind at low latitudes, and alternating fast and slow solar wind at mid latitudes, during the first and third orbits around solar minimum. The speeds are more variable in latitude during the second orbit around the maximum of solar cycle 23. The representative background observations from SOHO and Mauna Loa illustrate the difference in the streamer belt configuration for each orbit. Image reproduced with permission from McComas et al. (2008), copyright by AGU

Returning to the overview of the Ulysses mission, as shown in the right panel of Fig. 35, the second Ulysses orbit was dominated by the increased solar activity associated with solar cycle 23. As discussed by McComas et al. (2001, 2003), the simple solar wind structure observed during the first orbit was absent, and variable solar wind speeds, including intervals associated with interplanetary coronal mass ejections (e.g., Ebert et al. 2009; Du et al. 2010; Richardson 2014, and references therein) were observed at all latitudes. This is also evident in Fig. 37 from McComas et al. (2008), which shows the solar wind speed observed at Ulysses plotted as a function of latitude with the magnetic field direction indicated by blue (inward) or red (outward) during each of the three orbits. During the third orbit, commencing in mid-2004, solar activity was low, and the latitudinal solar wind configuration was once again similar to that during the first orbit, though with some differences (e.g., McComas et al. 2008; Ebert et al. 2013). In particular, the lower speed solar wind extends to higher latitudes, which may be related to the larger tilt angle of the heliospheric current sheet and the broader streamer belt during this minimum evident in images from the Solar and Heliospheric Observatory (SOHO) Extreme ultraviolet Imaging Telescope, the Mauna Loa K coronameter, and the SOHO C2 coronagraph.

**Fig. 38:**
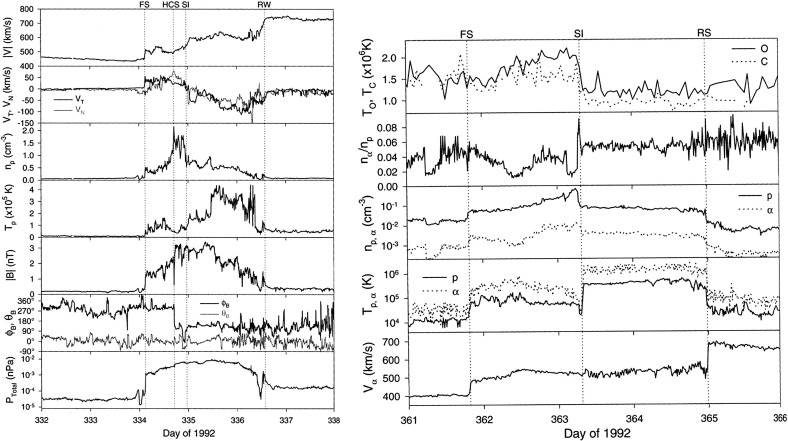
Left: An interaction region observed in November 1992 at 5 AU, $23^\circ$S. The parameters shown are: The solar wind speed and transverse and normal components, density, temperature, magnetic field intensity and polar and azimuthal angles, and the total pressure. Vertical lines indicate crossings of the forward shock (FS), heliospheric current sheet (HCS), stream interface (SI), and reverse wave (RW). Right: An interaction region observed in December 1992 showing the solar wind oxygen and carbon freezing in temperatures, alpha to proton ratio, densities and temperatures, and the alpha particle speed. Note the changes in the O and C freezing-in temperatures (i.e., charge states), and alpha/proton ratio at the stream interface indicating the different origins at the Sun of plasma on either side of the interface. Images reproduced with permission from [left] Forsyth and Gosling (2001), copyright by Springer; and [right] Wimmer-Schweingruber et al. (1997), copyright by AGU

**Fig. 39:**
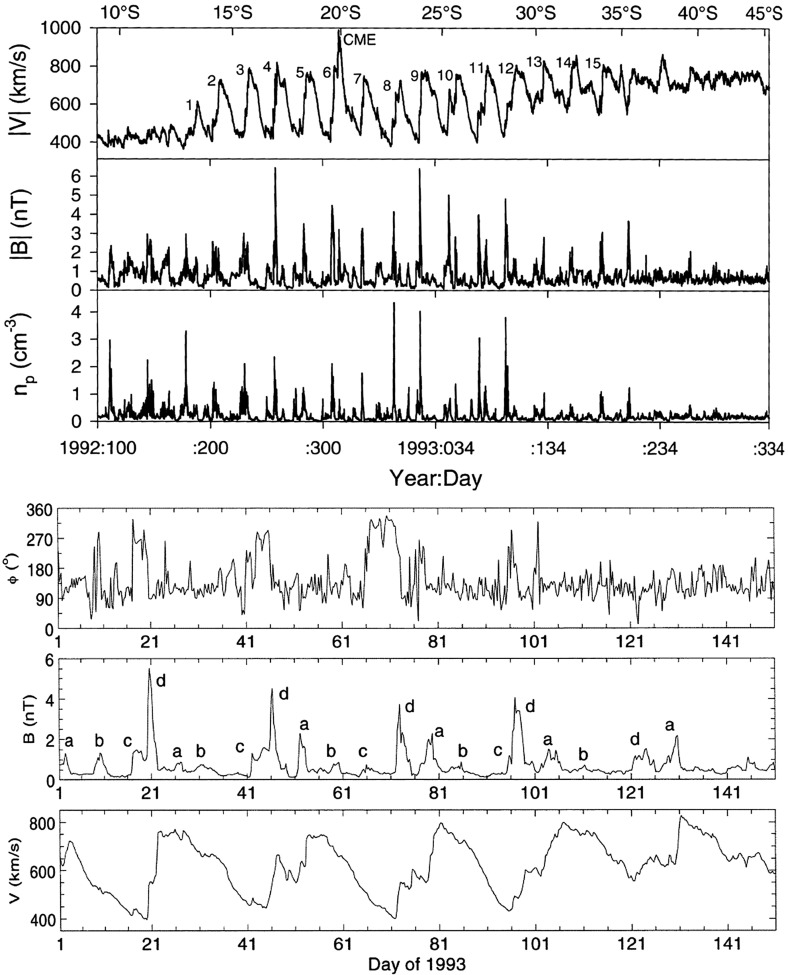
Top: Solar wind speed, magnetic field intensity, and density showing a sequence of stream interaction regions observed by Ulysses at $\sim 10$ to $45^\circ$S (Forsyth and Gosling 2001); the numbering follows Bame et al. (1993). Bottom: Magnetic field azimuth and intensity, and solar wind speed showing several southern hemisphere interaction regions (9–13 in the top panel) with recurrent features in the field intensity indicated by letters. Images reproduced with permission from [top] Bame et al. (1993), copyright by Springer, and [bottom] Smith et al. (1993), copyright by AGU

Figure 38 shows two examples of stream interaction regions observed by Ulysses. That in the left-hand panel was observed in November 1992, when the spacecraft was near 5 AU and $23^\circ$ south (Forsyth and Gosling 2001). This is rather similar to the interaction regions observed by the Pioneer 10 and 11 and Voyager spacecraft at several AU discussed in Sect. 5, and is bounded at the leading edge by a fast shock; the trailing edge shows a reverse “wave” that has not steepened into a shock. The stream interface (SI) may also be identified. This shows many of the characteristics already discussed in relation to observations at 1 AU including a slight increase in solar wind speed, a change in the flow direction (here indicated by the tangential and normal components of the flow velocity in the second panel), a fall in proton density, and an increase in proton temperature. At 1 AU, the pressure typically peaks in the vicinity of the interface, but here, the pressure is more dispersed through the interaction region. A crossing of the heliospheric current sheet (HCS) is also indicated ahead of the interface. The right-hand panel shows another interaction region, observed in December 1992 (Forsyth and Gosling 2001, adapted from Wimmer-Schweingruber et al. 1997). The main interest here is in identifying the stream interface in the center of the interaction region, which is bounded by forward and reverse shocks. The interface (see also Wimmer-Schweingruber et al. 1999) is associated with a fall in the oxygen and carbon “freezing-in” temperatures, increase in the alpha particle/proton ratio, decrease in alpha particle and proton densities, increase in the alpha particle and proton temperatures, and, in this case, no clear change in solar wind speed. The relatively abrupt changes in compositional/charge state parameters are consistent with the scenario that the interface is a distinct boundary between originally slow and fast solar wind at the Sun, as discussed in Sect. 3. Evidently, this boundary may persist to at least 5 AU with little or no mixing of the two plasma regimes. Wimmer-Schweingruber et al. (1997) note that in some interaction regions, the interface at Ulysses may be crossed multiple (but an odd number of) times.

The top panel of Fig. 39 from Forsyth and Gosling (2001) shows a sequence of southern hemisphere interaction regions showing the transition from slow solar wind at low latitudes through the region of alternating slow and fast streams to the persistent fast solar wind above $\sim 40^\circ$. The enhancements in the magnetic field intensity and proton density at the stream leading edges indicate the interaction regions, which weaken as the latitude increases due to the reduction in the speed transitions. Bame et al. (1993) note that the first appearance of the high-speed flows at Ulysses was not due to the spacecraft encountering faster flows as a result of the increase in latitude but to the development of an equatorial extension of a polar coronal hole. The later transition to continuous fast solar wind was, however, due to a latitude effect since the coronal hole configuration was stable (Phillips et al. 1994). The bottom panel shows several of these CIRs (numbers 9–13) in more detail (Forsyth and Gosling 2001, adapted from Smith et al. 1993) together with the disappearance of the heliospheric current sheet [crossings of the current sheet are indicated by transitions of the magnetic field azimuthal angle in the top panel between $\sim 100^\circ$ (inward polarity) and $270^\circ$ (outward)] above $28^\circ$S. The letters indicate various recurring magnetic field enhancements of which only ‘d’, associated with the interaction region at low latitudes, and ‘a’, which becomes associated with the reverse shock of the interaction region, persist to the end of this interval. Note the significant weakening of ‘d’ with increasing latitude.

Figure 40 from Gosling and Pizzo (1999) summarizes the strengths (defined as the ratio of the downstream to upstream solar wind densities) as a function of latitude of the forward and reverse shocks associated with the interactions during the southern (left) and northern (right) phases of the first orbit of the Sun. The southern shocks predominantly show a strengthening at mid latitudes (see also Burton et al. 1996), followed by weakening at higher latitudes. Furthermore, reverse shocks were observed up to higher latitudes than forward shocks, consistent with the configuration in Fig. 36. The northern-hemisphere shocks show a similar, though less organized pattern.

**Fig. 40:**
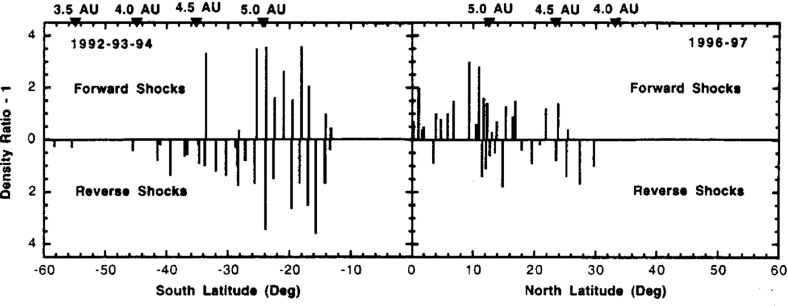
Strengths (ratio of the downstream to upstream plasma densities) of forward and reverse shocks observed by Ulysses in the southern or northern hemispheres during the first orbit of the Sun. Image reproduced with permission from Gosling and Pizzo (1999), copyright by Kluwer

**Fig. 41:**
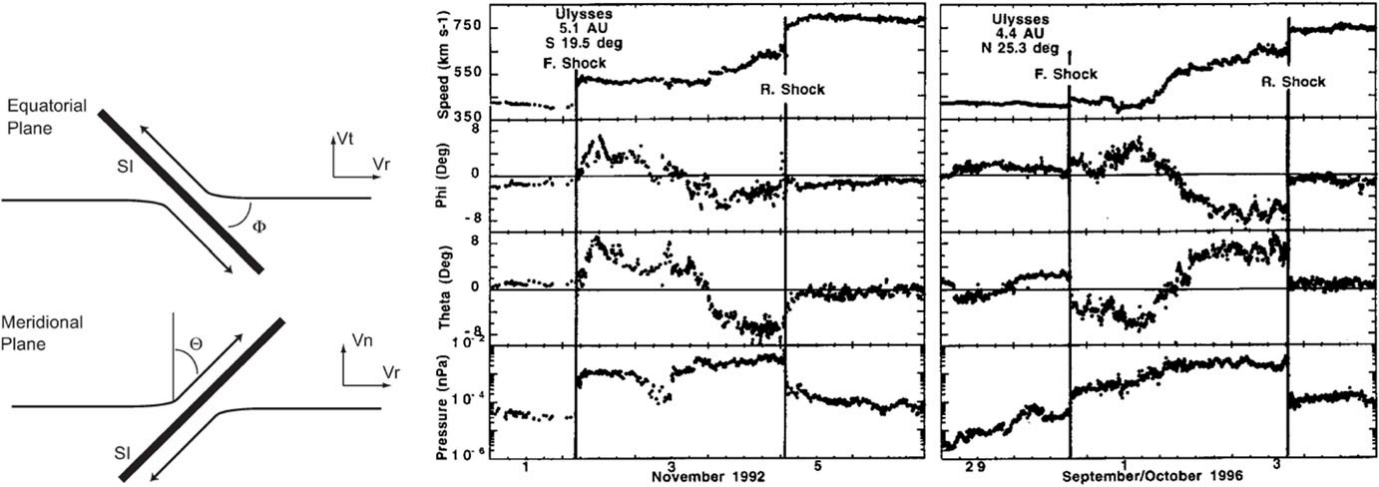
Left: Schematic of flow deflections in the equatorial and meridional planes; Southern-hemisphere (center) and northern-hemisphere (right) interaction regions showing similar patterns in the azimuthal flow direction (phi) but opposite patterns in the meridional flow angle (theta) that are in both cases consistent with equatorward deflections downstream of the forward shock and poleward downstream of the reverse shock. Images reproduced with permission from [left] (Riley et al. 2012a), copyright by Elsevier; and [right] Gosling and Pizzo (1999), copyright by Kluwer

As discussed above, near the ecliptic, solar wind flows are deflected in the east–west direction in a stream interaction region. In the scenario in Fig. 36, deflections in the meridional flow would also be expected, as illustrated in the left panel of Fig. 41. This figure also shows plasma parameters in two interaction regions, one in the southern hemisphere (center) and one in the northern (right), in particular the solar wind speed, azimuthal and meridional flow angles, and the proton pressure (Gosling and Pizzo 1999, based on Gosling et al. 1995 and Gosling et al. 1997). Both show similar deflection patterns in the azimuthal flow within the interaction region, consistent with the usual east–west deflection. However, the meriodinal angle variations follow opposite patterns in the two regions, in both cases being consistent with an equatorward deflection downstream of the forward shock and poleward deflection downstream (before passage) of the reverse shock, consistent with the expectation from Fig. 36.

**Fig. 42:**
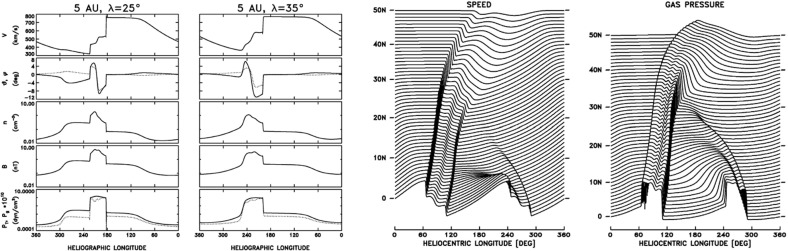
Results from the three-dimensional MHD interaction region model of Pizzo and Gosling (1994). Left: Simulated solar wind parameters for interaction regions at 5 AU and latitudes of 25 and $35^\circ$. The parameters are solar wind speed, north–south (solid) and east–west (dotted) flow angles, density, magnetic field intensity and total (solid) and gas (dotted) pressures. Right: Solar wind speed and gas pressure profiles versus longitude for latitudes of 0–$50^\circ$N showing the decay of first the forward then reverse shocks at increasing latitudes. Images reproduced with permission from Pizzo and Gosling (1994), copyright by AGU

Pizzo (1991, 1994), Pizzo and Gosling (1994), and Gosling and Pizzo (1999) discuss an MHD model of the three-dimensional evolution of stream interaction regions in a scenario similar to Fig. 36 with fast solar wind at high latitudes and slow solar wind from a tilted streamer belt that is consistent with the Ulysses observations. Figure 42 shows examples of results from this model. The left panels show the variation of various simulated solar wind parameters along a trajectory through an interaction region at 5 AU and at latitudes of 25 and $35^\circ$. In particular, the north–south (solid) and east–west (dotted) flow deflections in the second plot are similar to those observed in Fig. 41. The right-hand panels show traces at $1^\circ$ intervals in latitude at 5 AU for the solar wind speed and “gas pressure”. Two streams and interaction regions are evident at low latitudes, as well as the decay of first the forward shock then the reverse shock at the boundaries of the interaction regions with increasing latitude, also consistent with observations (Fig. 40).

**Fig. 43:**
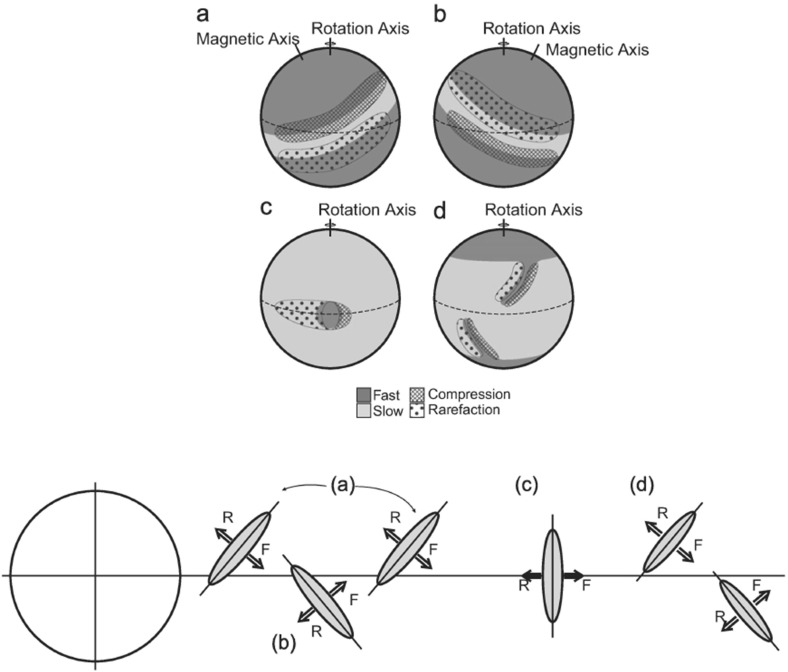
Examples of how different patterns of slow and fast solar wind near the Sun may give rise to interaction regions with different orientations further out in the heliosphere. Image reproduced with permission from Riley et al. (2012a), copyright by Elsevier

Figure 43 from Riley et al. (2012a) summarizes the various interaction region configurations that may result in the heliosphere from different patterns of slow and fast solar wind near the Sun. (a) and (b) show two views of a tilted dipole configuration with slow solar wind around the magnetic equator as discussed above, with opposite interaction region tilts in the northern and southern hemispheres and forward (reverse) shocks propagating equatorward (poleward). (c) shows an equatorial coronal hole with an approximately north–south aligned interaction region. Finally, (d) shows the case of equatorial extensions of both polar coronal holes, again giving inclined interaction regions, though the inclinations would depend on the particular configurations of the coronal holes.

## Energetic particle effects associated with stream interaction regions

There are two major energetic particle effects associated with stream interaction regions. The first is the acceleration of protons and other ions up to energies of $\sim 10$–20 MeV. Diffusive shock acceleration associated with the corotating forward and reverse shocks appears to be involved, but there is also evidence of additional acceleration, not involving shocks, within the interaction region. The second effect is the modulation of the galactic cosmic ray intensity, in particular a tendency for the GCR intensity to be depressed temporarily during the passage of an interaction region and high-speed stream. Both topics have been previously discussed in a review article by the author (Richardson 2004), so only a brief summary will be given here, together with a discussion of some related work since that article was written.

**Fig. 44:**
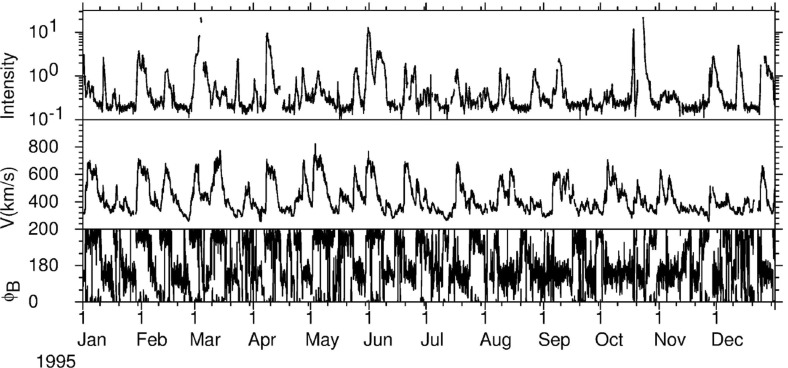
IMP 8 0.88–1.15 MeV proton intensity ((MeV s cms sr)$^{-1}$) during 1995, together with the solar wind speed and azimuthal angle of the IMF from WIND, showing proton increases predominantly associated with recurrent high-speed solar wind streams during this period of low solar activity at solar minimum. Image reproduced with permission from Richardson (2004), copyright by Kluwer

### Particle acceleration in the vicinity of stream interaction regions

Energetic ion enhancements with energies of a few MeV/n recurring at the solar rotation period were initially reported by Bryant et al. (1965) who interpreted them as evidence for a continuous “drizzle” of particles from localized regions on the Sun. Other examples were reported by Fan et al. (1965), Wilcox and Ness (1965), Fan et al. (1968), and McDonald and Desai (1971). These particle events were unusual compared to “normal” solar energetic particle events (see Desai and Giacalone 2016 for a recent review of solar particle events) in showing no clear association with solar activity (e.g., flares), rise and decay times of a few hours, no velocity dispersion at onset (faster particles do not arrive earlier), similar spectra at onset and event maximum, soft spectra, and weak directional anisotropies. McDonald and Desai (1971) noted that these particle events occurred within corotating high-speed streams and suggested that they were particles that were either continually accelerated or stored at the Sun and then escaped along open field lines above the sources of the high-speed streams (yet to be identified as coronal holes), a view also supported by, for example, Roelof and Krimigis (1973), Nolte and Roelof (1977), and Gold and Roelof (1979). Figure 44 shows the $\sim 1$ MeV proton intensity at IMP 8 and the solar wind speed and azimuthal angle of the IMF from WIND during 1995. The proton intensity during this period at solar minimum was dominated by similar enhancements within recurrent high-speed solar wind streams.

**Fig. 45:**
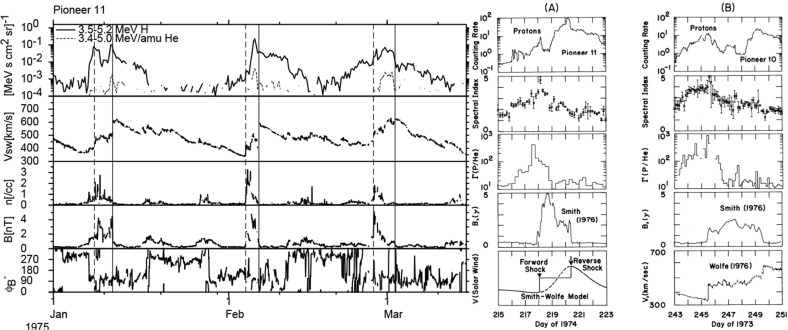
Left: Stream interaction region-associated particle enhancements (3.5–5.2 MeV protons and 3.4–5.0 MeV/n He) observed by the Goddard experiment on Pioneer 11 at $\sim 4.6$ AU in early 1975, showing intensity increases in the vicinity of forward and reverse shocks (dashed and solid vertical lines, respectively). Right: 0.5–1.8 MeV proton intensity in the vicinity of the forward and reverse shocks of two interaction regions observed by Pioneer 10 or 11. Other parameters shown are the proton differential spectrum power law index, proton/alpha particle ratio ($\Gamma$), magnetic field intensity and solar wind speed. Note that the reverse shock particle enhancement has a harder spectrum (smaller spectral index) and smaller proton/alpha particle ratio than the enhancement at the forward shock. Images reproduced with permission from [left] Richardson (2004), copyright by Kluwer; and from [right] Barnes and Simpson (1976), copyright by AAS

A major advance in understanding recurring particle events came from combining observations from the Helios spacecraft inside 1 AU, spacecraft near Earth, and the Pioneer 10 and 11 spacecraft beyond 1 AU (McDonald et al. 1976; Barnes and Simpson 1976; Kunow et al. 1977; Van Hollebeke et al. 1978, 1979; Christon and Simpson 1979). In particular, the Pioneer observations indicated that these particle events tend to peak in intensity in the vicinity of interaction regions, and more specifically, adjacent to the forward or reverse shocks, as illustrated in Fig. 45. The right panel (Barnes and Simpson 1976) illustrates that the particle enhancements at the forward and reverse shocks may have different characteristics, in particular a harder spectrum and larger alpha particle abundance at the reverse shock.

The left panel of Fig. 46 from Barnes and Simpson (1976) shows several corotating particle events tracked from IMP 8 to Pioneer 11 and Pioneer 10 by assuming that the related interaction regions follow an Archimedean spiral configuration. (As noted by Richardson 2004, the first two Pioneer enhancements should probably map to the enhancements marked ‘a’ and ‘b’ at IMP 8 rather than to the solar events ‘S’ indicated by the authors.) By following such events between spacecraft, and also to the Helios spacecraft inside 1 AU, Van Hollebeke et al. (1978) obtained the intensity variation (for 0.9–2.2 MeV protons) with heliocentric distance shown in the right panel. This clearly shows that peak intensities in corotating particle events occur at a few AU. The intensities then decline towards the Sun (with gradients of $\sim 100\%$/AU), and also further out in the heliosphere. A solar origin is therefore convincingly ruled out in favor of acceleration in the solar wind. Evidence of sunward streaming in corotating particle events (e.g., Marshall and Stone 1978; Mewaldt et al. 1978; Van Hollebeke et al. 1978; Christon 1981; Zwickl and Roelof 1981; Richardson 1985a; Richardson et al. 1993) also supports this conclusion.

**Fig. 46:**
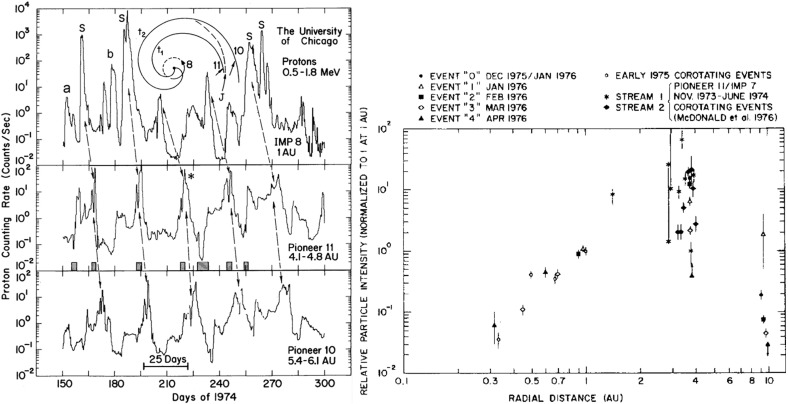
Left: 0.5–1.8 MeV proton counting rates at IMP 8 and Pioneers 10 and 11, located at 1 AU, 5.4–6.1 AU and 4.1–4.8 AU, respectively, during a period in 1974. Particle enhancements that corotate from one spacecraft to another are indicated. (As discussed by Richardson (2004), the first two Pioneer events probably map to the corotating events marked ‘a’ and ‘b’ rather then to the solar events (S) suggested by Barnes and Simpson (1976). Shaded rectangles indicate interaction regions at Pioneer 11. Right: Intensity of 0.9–2.2 MeV protons measured by the Helios, Pioneer 10/11 and near-Earth spacecraft (normalized to observations at 1 AU) in several corotating particle events plotted against radial distance from the Sun. Note that the intensities are largest at a few AU, and decline closer to the Sun, clearly arguing against a solar origin and favoring acceleration at interaction regions in the outer heliosphere. The intensities also decline in the most distant observations, consistent with the decay of interaction regions at these distances discussed in Sect. 5. Images reproduced with permission from [left] Barnes and Simpson (1976), copyright by AAS; and from [right] Van Hollebeke et al. (1978), copyright by AGU

**Fig. 47:**
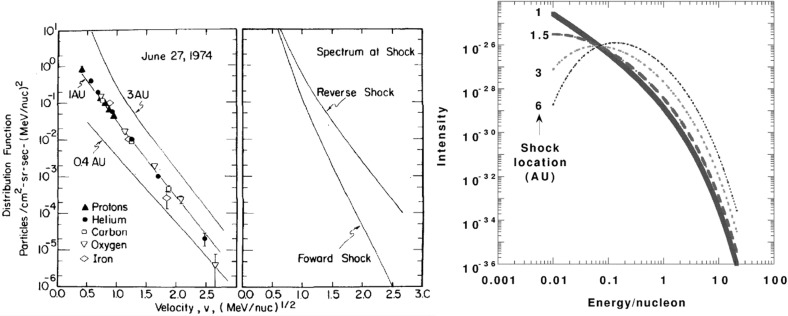
Particle distribution functions calculated by Fisk and Lee (1980) (left panel) for three heliocentric distances, with a fit to observations at 1 AU, and (center) at CIR forward and reverse shocks, assumed to be at 4 AU. Right: Differential energy spectra at 1 AU predicted by the Fisk and Lee (1980) model assuming different locations of the accelerating shock beyond 1 AU. Only if the source is near 1 AU is there no low energy turn down. Images reproduced with permission from [left] Fisk and Lee (1980), copyright by AAS; and from [right] Mason et al. (1999), copyright by Kluwer

Particle scattering from Alfvén waves and other turbulence has been proposed as a possible acceleration mechanism for corotating events (McDonald et al. 1976; Fisk 1976a, b), though this may not produce the correct radial gradients (Christon 1981). Rather, the tendency for intensities to peak near corotating forward and reverse shocks suggests energization by diffusive shock acceleration as a probable mechanism, as discussed for example by Palmer and Gosling (1978), Pesses et al. (1978, 1979), Hamilton et al. (1979), Fisk and Lee (1980), Scholer et al. (1980), Christon (1981), Decker et al. (1981), Classen et al. (1998), Keppler (1998), Simnett et al. (1998) and Scholer (1999). In particular, Fisk and Lee (1980) proposed a model in which acceleration at the interaction region shocks occurs by particle scattering between the shock and upstream magnetic fluctuations. The particles then stream sunward from the shocks at several AU. Thus, particles observed within the fast stream closer to the Sun would be expected to be accelerated at the reverse shock. Reasonable choices for the model parameters, such as the solar wind speed, shock connection distance, shock strength, and particle mean free path, give spectra and radial gradients that are consistent with those observed when the particles have streamed sunward to the inner heliosphere, at least at energies of a few MeV where unusual spectra of the form $f\propto exp(-v/v_o)$, where *f* is the distribution function and *v* is the particle speed, were reported (Gloeckler et al. 1979b, a), as shown in the left panel in Fig. 47. The center panel suggests that the particle spectrum at the reverse shock predicted by the Fisk and Lee (1980) model should be harder than that at the forward shock, a possible factor contributing to differences between the particle enhancements associated with the leading and trailing edges of interaction regions as indicated in Fig. 45. The harder reverse shock spectrum may also help to explain why particles are observed preferentially inside high-speed streams in the inner heliosphere rather than in the slow solar wind on field lines linking to the interaction region forward shock (cf. Fig. 14).

**Fig. 48:**
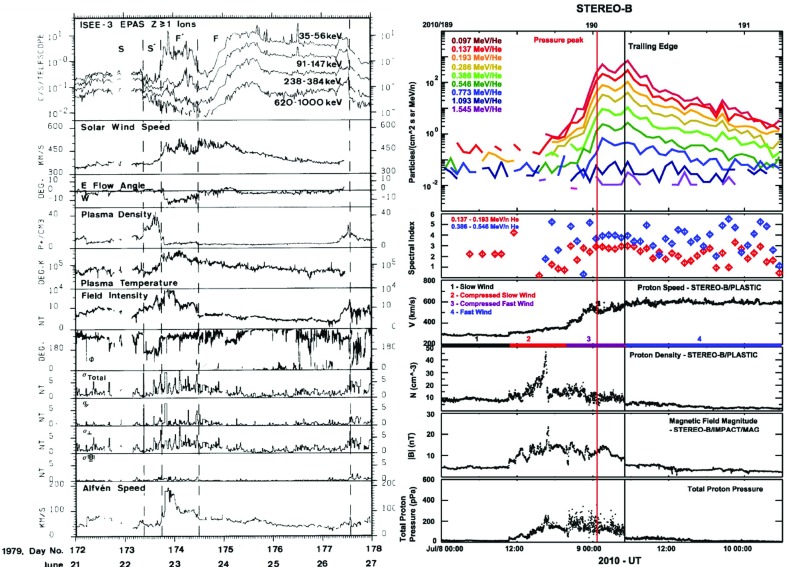
Examples of particle enhancements associated with stream interaction regions at 1 AU that extend down to less than 100 keV (left: at ISEE 3; right: at STEREO B). In neither case are forward or reverse shocks present at the spacecraft. Note that the particle enhancements commence inside the trailing edge of the interaction region following the interface. In the left-hand case, the enhancement extending up to $\sim 200$ keV is separate from the later particle increase extending to higher energies. This panel also shows several parameters indicating the enhanced solar wind temperature, magnetic field turbulence and higher Alfvén speeds in the trailing edge of the CIR that may be conducive to particle acceleration from the tail of the solar wind distribution in this region. Images reproduced with permission from [left] Richardson and Hynds (1981), Richardson (1985b), copyright by Elsevier; and from [right] Ebert et al. (2012), copyright by AAS

A characteristic of the Fisk and Lee (1980) spectra, however, is that when plotted as more conventional differential energy spectra ($dJ/dE\sim f(E)$), they turn down at low energies (below a few 100 keV). This is because the inclusion of adiabatic deceleration in the expanding solar wind in the model means that it is more difficult for lower energy particles to propagate from the shocks at several AU into the inner heliosphere. The right-hand panel of Fig. 47 from Mason et al. (1999) shows spectra at 1 AU calculated using the Fisk and Lee (1980) model that illustrate how the low energy turn down varies with different assumptions for the location of the accelerating shock. Only if acceleration occurs relatively close to 1 AU is there no significant turn down. In fact, many observations have demonstrated that the spectra of recurrent events at $\sim 1$ AU do not show such a turn down but extend down to energies of at least tens of keV/n (e.g., Richardson and Hynds 1981, 1990; Zel’dovich et al. 1981; Richardson 1983; Logachev et al. 1990; Mason et al. 1997, 1999, 2008; Gómez-Herrero et al. 2011; Ebert et al. 2012; Filwett et al. 2017) and appear to merge with the suprathermal tail of the solar wind distribution (Chotoo et al. 2000; Yu et al. 2017). This suggests that these particles are accelerated relatively close to 1 AU, possibly predominantly out of the tail of the local solar wind distribution, and not exclusively at distant corotating shocks. Two examples of particle events associated with stream interaction regions at 1 AU and extending to energies below 100 keV are shown in Fig. 48. No shocks are present at the observing spacecraft, suggesting that the particles are not accelerated at nearby shocks. In both cases, as is typical, the particle enhancements commence inside the trailing edge of the interaction region following the interface (F$^{\prime }$ region in Fig. 14). The left-hand panel of Fig. 48 indicates the enhanced magnetic field fluctuation levels, solar wind temperature, and Alfvén speed in this region that led Richardson (1985b) to suggest that this may be a favorable location for the stochastic acceleration of particles from the tail of the solar wind plasma distribution. Particle acceleration by scattering from converging scattering centers in the speed gradient within the interaction region has also been proposed (e.g., Jokipii et al. 2001; Giacalone et al. 2002; Jokipii et al. 2003; Malakit et al. 2003).

**Fig. 49:**
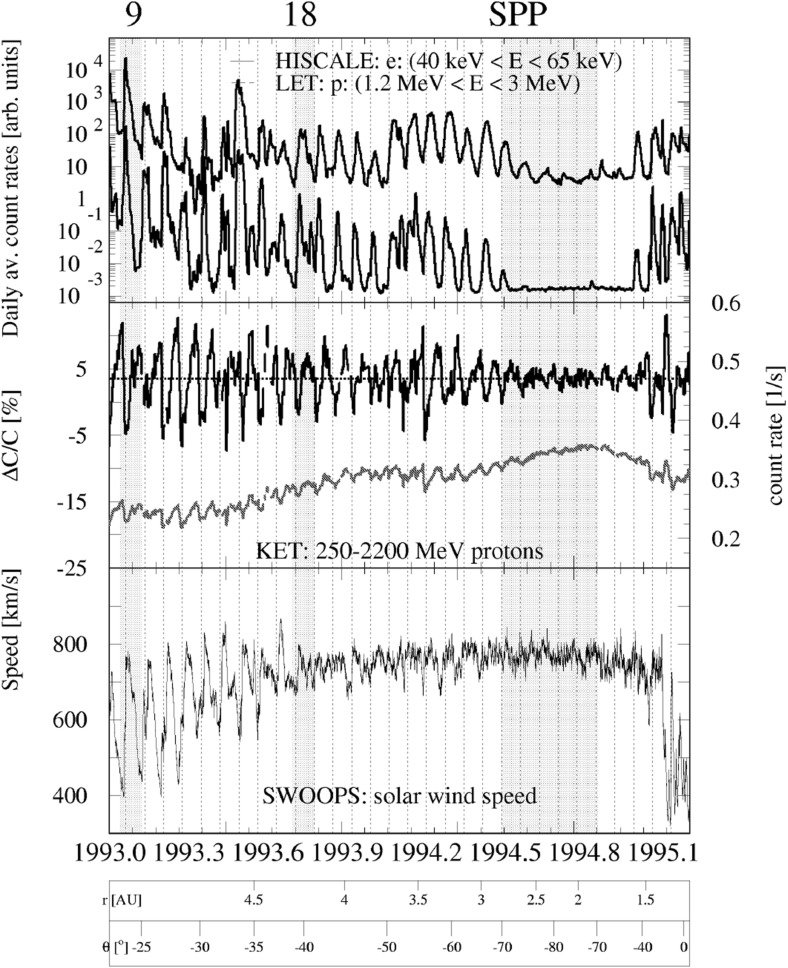
Ulysses energetic particle and solar wind speed observations in 1993–early 1995 when Ulysses moved from $\sim 5$ AU, $25^\circ$S, passed over the south solar pole at $\sim 2$ AU, and descended rapidly towards the equator. Vertical dashed lines are at 26-day intervals. At low latitudes, Ulysses sampled alternately fast and slow solar wind and the associated interaction regions and was continually in fast polar coronal hole flows above $40^\circ$. Recurrent low energy proton and electron enhancements extend up to $\sim 70^\circ$S for protons, and to the highest latitudes of Ulysses for electrons, and are accompanied by depressions in the galactic cosmic ray intensity evident in the 250–2200 MeV proton count rate (both the original rate and detrended data (percentage deviation from the mean) are shown). Image reproduced with permission from Heber et al. (1999), copyright by Elsevier

Recurrent particle events associated with interaction regions were also observed by Ulysses. In particular, observations made as the spacecraft moved southward towards the first polar passage showed that these particle events were observed well beyond the latitudes at which the interaction regions and corotating shocks were present. This was unexpected since if particles are accelerated at interaction region shocks and then travel along Parker spiral field lines, which lie on cones of constant latitude, they should not extend beyond the latitude range of the shocks; see Keppler (1998), Simnett et al. (1998), Kunow et al. (1999), Lanzerotti and Sanderson (2001) and Heber et al. (1999), for reviews of these observations. Figure 49 from Heber et al. (1999) shows a synopsis of Ulysses particle and solar wind speed observations in 1993–early 1995, when the spacecraft was climbing from $\sim 20^\circ$S at $\sim 5$ AU from the Sun to $>70^\circ$S during south polar passage (SSP). Vertical dashed lines indicate solar rotation intervals. The top panel shows 40–65 keV electron and 1.2–3 MeV proton count rates. These are both clearly dominated by recurrent particle increases that are observed up to $70^\circ$S for protons, while electron increases continued to be observed throughout the south polar pass. In contrast, the solar wind speed in the bottom panel indicates that the variations in speed associated with interaction regions only extended up to about $40^\circ$S. Two processes have been proposed to account for these observations. First, field lines may deviate from the simple Parker spiral field. In particular, Fisk (1996) proposed an extension of the Parker model incorporating the interplay between differential rotation of the photosphere and non-radial expansion from more rigidly-rotating polar coronal holes, which could bring high-latitude field lines at Ulysses down to lower latitudes, within the range of the interaction regions, further from the Sun. Second, cross-field diffusion may transport particles to latitudes that are not magnetically connected to lower latitude interaction regions (e.g., Kóta and Jokipii 1995, 1998).

**Fig. 50:**
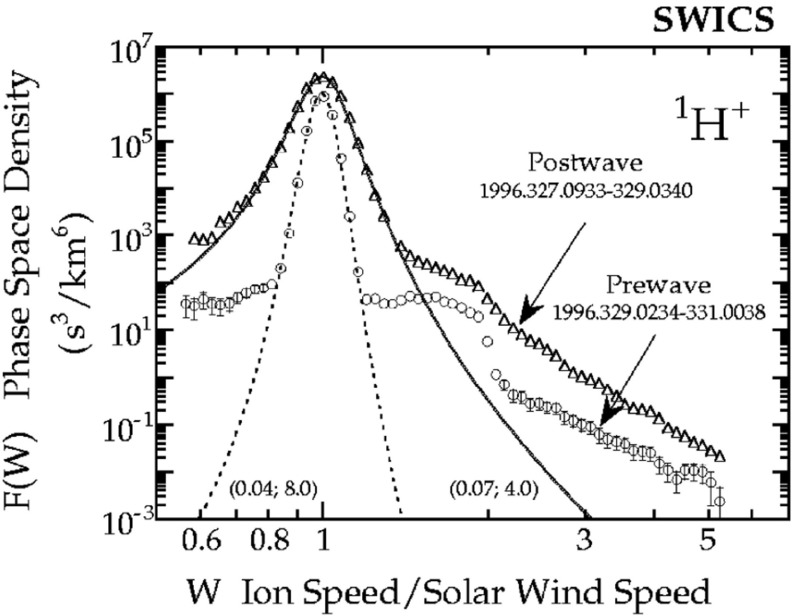
Proton distribution functions observed at 4.6 AU by the SWICS instrument on Ulysses in the vicinity of an interaction region ‘reverse wave’. The high velocity tails of the distributions consist of the interstellar pickup ion distribution at $\le 2$V and a population of locally-accelerated particles extending up to at least the upper limit of SWICS. Image reproduced with permission from Gloeckler (1999), copyright by Kluwer

**Fig. 51:**
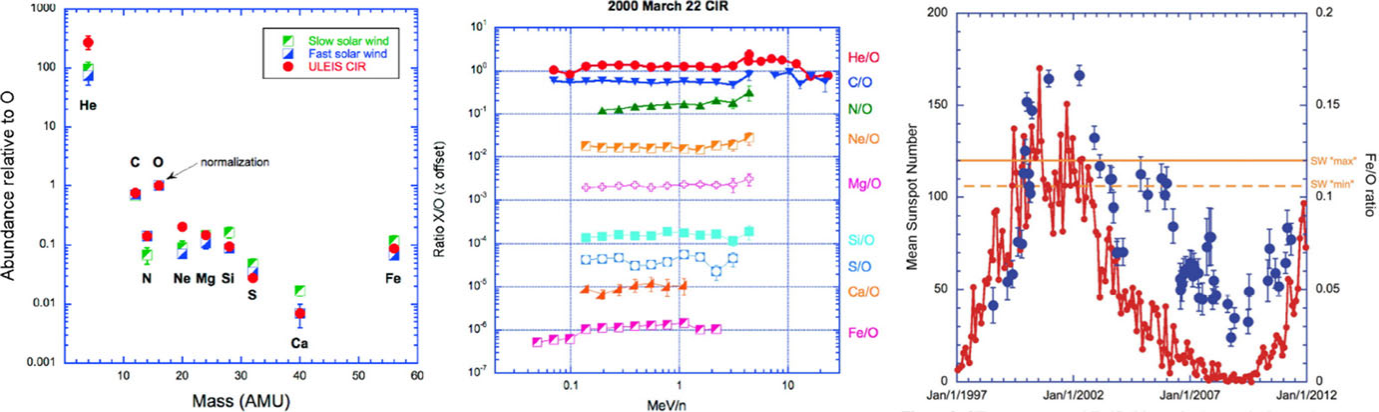
Left: Comparison of the abundances relative to oxygen summed over 41 corotating particle events, and in slow and fast solar wind, showing the general similarity (except He and Ne) with fast solar wind abundances. Center: Abundances relative to oxygen as a function of energy/amu (with arbitrary vertical displacements) in a corotating event indicating that the abundances are nearly independent of energy. Right: The variation in Fe/O in corotating events with solar activity (sunspot number, red) suggests a contribution from Fe-rich solar flare particles. Images reproduced with permission from [left, center] Mason et al. (2008) and from [right] Mason et al. (2012), copyright by AAS

Ulysses also provided comprehensive observations demonstrating how the energetic ion distribution function extends from the solar wind distribution in stream interaction regions. For example, Fig. 50 from Gloeckler (1999) illustrates proton distribution functions measured by the SWICS instrument at 4.6 AU and $21.4^\circ$ latitude upstream and downstream of a ‘reverse wave’ at the trailing edge of an interaction region. The suprathermal tails on the thermal proton distributions extend to at least five times the solar wind speed ($\sim 60$ keV) suggesting that the solar wind plasma is a major source of the particles extending down to similar energies accelerated in interaction regions. Particle composition measurements from Ulysses and other spacecraft (e.g., McGuire et al. 1978; Gloeckler et al. 1979a, b; Scholer et al. 1979; Christon and Simpson 1979; Scholer et al. 1980; von Rosenvinge and McGuire 1985; Dietrich and Simpson 1985; Reames et al. 1991; Marsden et al. 1993; Maclennan et al. 1993; Richardson et al. 1993; Mason et al. 1997; Fränz et al. 1999; Mason et al. 1999; Bučík et al. 2009; Mason et al. 2012; Bučík et al. 2012; Zel’dovich et al. 2016; Filwett et al. 2017) also support this view. For example, the left-hand panel of Fig. 51 from Mason et al. (2008) shows the similarity between abundance ratios (normalized to oxygen) for various ions summed over 41 corotating particle events observed near Earth in 1998–2007 and in the solar wind, in particular fast solar wind. There are also some differences, most notably the higher relative abundances of He and Ne. The center panel of Fig. 51 shows various abundance ratios relative to oxygen (with arbitrary offsets to separate the elements) as a function of energy/mass for one event. The relatively constant ratios indicate that the spectra have similar shapes for all these ions. Mason et al. (2008) (see their Table 2) highlight the differences in abundances in corotating events and solar energetic particle events associated with flares and coronal mass ejections that indicate that solar particle events do not usually provide a source population for acceleration at interaction regions. However, they can on occasions (e.g., Fränz et al. 1995; Richardson et al. 1998; Bučík et al. 2012). Furthermore, the right-hand panel in Fig. 51 from Mason et al. (2012) shows that the variation in the *Fe* / *O* ratio in recurrent events followed the solar cycle in 1997–2012, suggesting that an Fe-rich population associated with solar events contributes to the source for corotating events at higher solar activity levels (Filwett et al. 2017 show a similar cycle dependence for the Fe/CNO ratio). In contrast, the average solar wind Fe/O ratio shows little variation during the solar cycle. The detection of $^3$He in corotating events at levels far exceeding those in the solar wind (Mason et al. 2008) also suggests that particles from $^3$He-rich “impulsive” solar events accelerated by flares contribute to the source population for corotating events.

**Fig. 52:**
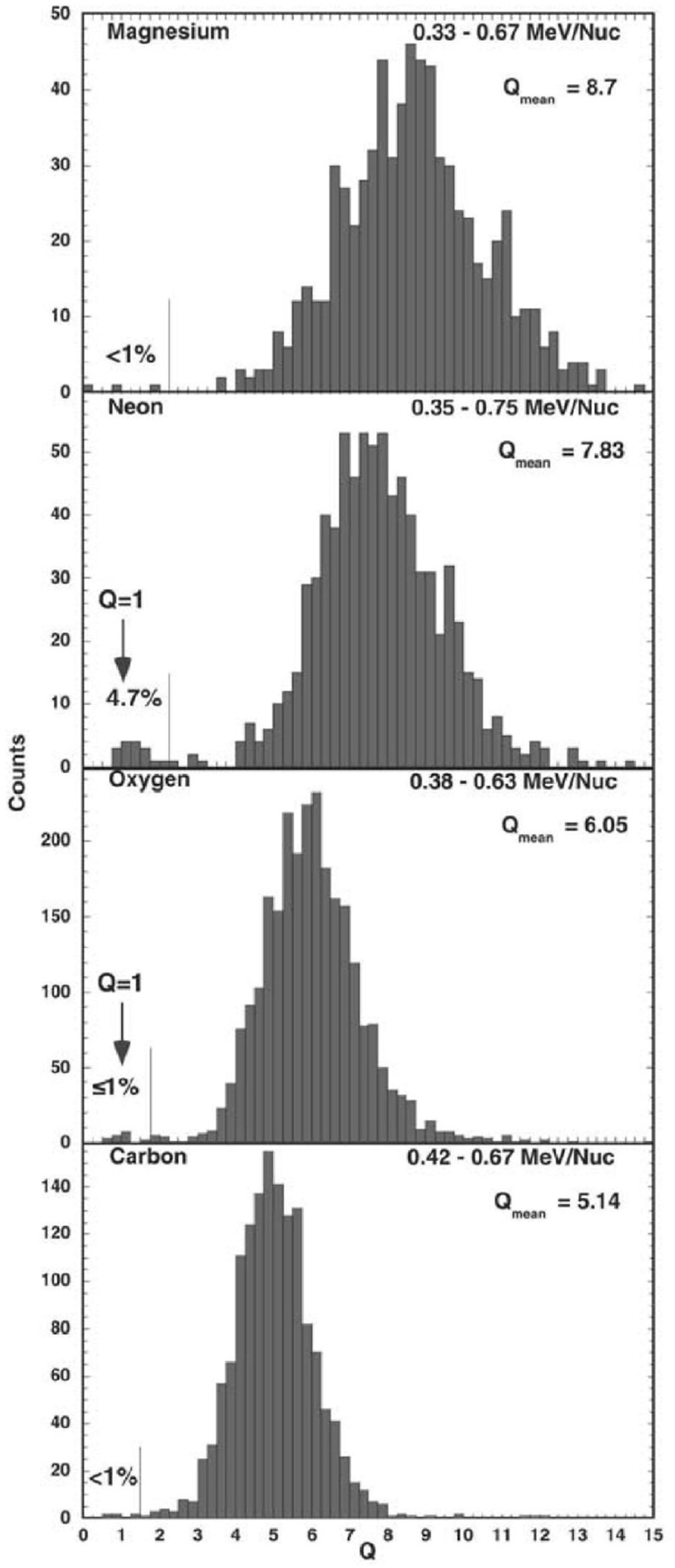
Heavy ion charge states integrated over six corotating particle events in 1999–2000, observed by the ACE/SEPICA instrument showing the negligible ($<1\%$) fraction of singly-charged ions, suggesting little contribution from a pickup ion source, except for Ne ($\sim 4.7\%$). Image reproduced with permission from Möbius et al. (2002), copyright by AGU

Returning to Fig. 50, the conspicuous drops in the distribution functions at an ion speed of $\sim 2V$ are evidence of an interstellar “pick up” proton component (Vasyliunas and Siscoe 1976) in the interaction region energetic particle population. (Briefly, interstellar neutral atoms streaming through the solar system may be ionized by charge exchange with the solar wind or photo-ionization near the Sun. Once ionized, they are picked up by the solar wind magnetic field and accelerated up twice the solar wind speed. For a review of pick up ions in the heliosphere, see Kallenbach et al. 2000.) SWICS also observed a similar pick up feature in the He$^+$ spectrum, and He$^+$ has also been observed in interaction regions near 1 AU (Chotoo et al. 2000; Hilchenbach et al. 1999). He$^+$, which may reach $\sim 25\%$ of the He$^{2+}$ abundance at 1 AU (Möbius et al. 2002) must be of interstellar origin since solar wind He is essentially fully ionized (He$^+$/He$^{2+}<5\times 10^{-5}$, Gloeckler and Geiss 1998). Schwadron et al. (1996) and Chen et al. (2015) discuss models of interstellar pick-up ion acceleration in interaction regions. Accelerated He pick up ions may help to account for the enhanced abundance of He relative to the solar wind evident in the left-hand panel of Fig. 51. A heavy ion contribution (e.g., C, Mg, Si) from an “inner source” of pick up ions released from interplanetary dust has also been proposed (Geiss et al. 1995a; Gloeckler et al. 2000).

Ion charge states provide a way to distinguish between pick up ions (singly charged) and ions accelerated from the bulk solar wind distribution or the suprathermal tail. In particular, Möbius et al. (2002) (using direct observations of charge states from the SEPICA instrument on the ACE spacecraft) and Mazur et al. (2002) (using 0.5–1.0 MeV/n data from SAMPEX and a geomagnetic cut-off method to determine charge state) both concluded that the majority of ions in corotating events have charge states similar to those of the solar wind, consistent with a solar wind source, and that singly charged pick up ions are relatively rare. Figure 52 from Möbius et al. (2002) shows Mg, Ne, O and C ion charge states summed over 6 corotating events, illustrating the lack ($<1\%$) of singly charged ions except for a 4.7% contribution for Ne. Similar upper limits were inferred by Mazur et al. (2002).

Much of the focus on particle acceleration at interaction regions has been on ions but as noted in relation to Fig. 49, recurrent energetic (tens of keV) *electron* enhancements were detected up to highest heliographic latitudes attained by the spacecraft (Simnett and Roelof 1995; Roelof et al. 1996). Considering observations at 1 AU, Anderson (1969) reported enhancements of low energy protons and $>20$ keV electrons un-associated with large solar flares that might have been related to interaction regions; pure electron events were rare and pure proton events relatively common. Zel’dovich et al. (1981) and Mineev et al. (1981a, b) reported recurrent low energy proton events at 1 AU in 1975–1977 accompanied by 40 keV–$\sim 1$ MeV electron enhancements. However, McDonald et al. (1976) found no correlation between recurring ion enhancements in the outer heliosphere and MeV electrons. Richardson (1983) noted that the $> 15$ keV electron flux was above background in five of the nine stream-associated low energy ion events studied, though the electron and ion temporal profiles were usually different. Scholer et al. (1999) suggest that acceleration by stochastic processes is unlikely to occur because energetic electrons are essentially scatter-free in interplanetary space, and that shock drift acceleration is a more likely acceleration mechanism, as also proposed by Simnett and Roelof (1995) and Roelof et al. (1996). In support of this, Mann et al. (2002) reported a correlation between the 30–50 keV electron intensity and the magnetic field compression at interaction region shocks observed by Ulysses.

### Modulation of galactic cosmic rays by interaction regions and high-speed streams

This topic is also reviewed in further detail by Richardson (2004); see also McKibben et al. (1999). Galactic cosmic rays are energetic charged particles believed to be accelerated by objects such as supernova remnants. After modulation in the heliospheric magnetic field (e.g., Potgieter 1998, 2013) they are observed in the inner heliosphere with maximum intensities at energies of $\sim 0.5$ GeV (Lockwood and Webber 1996). Modest cosmic ray depressions apparently modulated by the 27-day solar rotation period and associated with recurrent geomagnetic activity enhancements were among the first phenomena discovered using the world-wide network of ionization chambers set up by Forbush (see Van Allen 2013). Then in 1949–1951, using aircraft-mounted neutron monitors, Simpson demonstrated that short-term variations in cosmic ray intensity were not caused by changes in the geomagnetic field but were imposed by conditions in the interplanetary medium that were ultimately controlled by the Sun (Simpson 2000, and references therein). Meyer and Simpson (1955) then inferred that the 27-day cosmic ray variations were more prominent during the minimum of the 11-year sunspot cycle. After unsuccessful attempts using other solar features, Simpson et al. (1955) concluded that the 27-day cosmic ray variations were closely correlated with recurring unipolar magnetic field regions above the photosphere of the Sun later identified with coronal holes and the source of corotating high-speed streams. For a review of recurrent GCR modulations from a historical perspective, see Simpson (1998).

Figure 53 from Richardson et al. (1996) shows examples of recurrent GCR intensity modulations observed by the Helios 1 and 2 spacecraft, and at IMP 8 in Earth orbit, in January–March, 1976. The solar wind speed and cosmic ray intensity are shown for each spacecraft. The GCR observations here are counting rates of the anti-coincidence guards of the IMP 8 GME and University of Kiel instruments on the Helios spacecraft; see Richardson (2004) for further discussion of the use of these rates for GCR studies. The inset figure shows the Helios spacecraft locations relative to the Earth. The near-ecliptic inner heliosphere during this period was dominated by three intervals of high-speed solar wind flows, which corotated twice past each spacecraft during this interval (there are gaps in the solar wind speed at IMP 8 when the spacecraft was inside Earth’s bow shock). The high-speed streams are accompanied by depressions in the guard count rates of $\sim 1$–5%, which endure through the passage of the streams and tend to be anti-correlated with the solar wind speed. Consistent with the spacecraft locations, they corotated first past Helios 1, then Helios 2 and finally IMP 8 around 2 days later. Other examples of cosmic-ray depressions within corotating streams near the Earth are shown in Fig. 18, while Fig. 49 shows recurrent modulations of GCRs observed by Ulysses extending up to high latitudes, well beyond the latitudinal range of the interaction regions, during the first southern latitude pass. Interestingly, Dunzlaff et al. (2008) note that such high latitude GCR modulations were absent during the southern pass of Ulysses’ third orbit, also at solar minimum, which they attribute to the absence of a large stable coronal hole structure in this minimum.

**Fig. 53:**
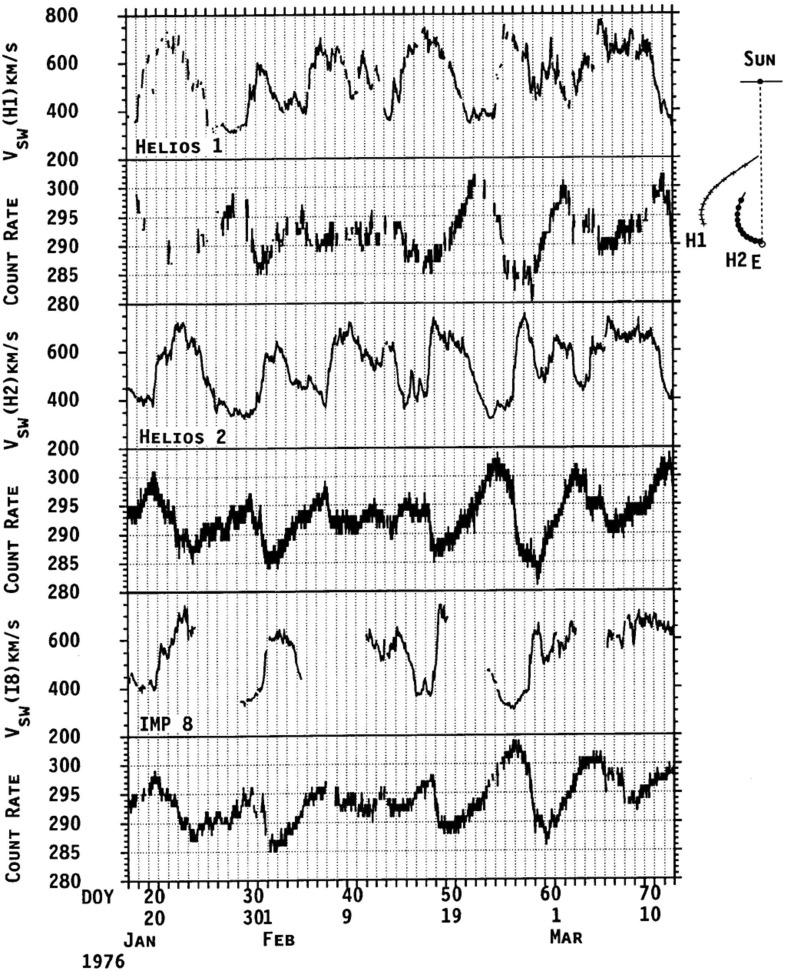
Corotating galactic cosmic ray depressions associated with high-speed solar wind streams observed by Helios 1, Helios 2 and by IMP 8 in Earth orbit, in January–March, 1976; the inset figure shows the spacecraft locations during this interval. The GCR observations are the counting rates (/s) of the anti-coincidence guards of the University of Kiel instruments on the Helios spacecraft and the GME instrument on IMP 8. Gaps in the IMP 8 solar wind speed indicate when the spacecraft was inside Earth’s bow shock. Image reproduced with permission from Richardson et al. (1996), copyright by AGU

Numerous studies (e.g., Lockwood 1960; Iucci et al. 1979; Duggal et al. 1981; Venkatesan et al. 1982; Burlaga et al. 1984; Newkirk and Fisk 1985; Richardson et al. 1996, 1999; Dumbović et al. 2011; Badruddin and Kumar 2016, and references therein), have examined the relationship between corotating features in the solar wind and GCR time variations with the aim of inferring the physical processes that give rise to these modulations. Briefly [based on Parker’s particle transport equation (Parker 1965b)] there are several processes that may contribute: (1) Increased turbulence in the interaction region may impede the entry of cosmic rays into the region sunward of the interaction region (e.g., McCracken et al. 1966; Burlaga et al. 1984; Kóta and Jokipii 1991, 1998; Quenby et al. 1995). With the usual assumption that turbulence levels scale with B, then the modulation (similar to the “CR-B” relation inferred by Burlaga et al. (1985) from Voyager observations in the outer heliosphere) would be expected to commence at the leading edge of the magnetic field enhancement associated with the interaction region. However, as noted above, turbulence levels tend to be higher following the interface, and hence may not strictly follow B in the interaction region. (2) Cosmic rays are swept away from the Sun more efficiently in the fast solar wind (e.g., Richardson et al. 1996). The close anti-correlation between solar wind speed and GCR intensity in Fig. 53 may be suggestive of this mechanism, and modulation might be expected to increase in the vicinity of the high-speed stream leading edge, often also near the stream interface; (3) GCR transport models that include particle drifts due to gradients and curvature in the heliospheric magnetic field predict latitudinal intensity gradients that are organized about the heliospheric current sheet. Thus, intensity modulations are observed as the distance between the HCS (corotating with the Sun) and observing spacecraft varies (e.g., Newkirk and Fisk 1985; Badruddin et al. 1985; 4) Enhanced drifts of particles out of interaction regions due to the stronger fields within them lead to GCR depressions in interaction regions (Barouch and Burlaga 1975, 1976).

**Fig. 54:**
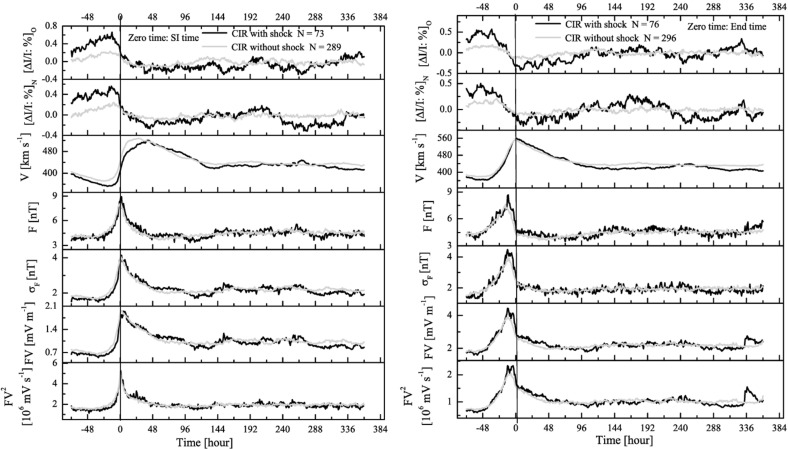
Superposed epoch analysis of GCR intensity variations during the passage of interaction regions and high-speed streams observed by the Oulu and Newark neutron monitors (top two panels), and the solar wind speed, magnetic field intensity (*F*), standard deviation of the magnetic field vector and *FV* and $FV^2$. In the left-hand panel, the stream interface is chosen as zero epoch, while the trailing edge of the interaction region is used in the right-hand panel. This choice clearly influences the profiles that result from the analysis; results are shown for interaction regions with (black graphs) or without forward shocks (grey graphs). Image reproduced with permission from Badruddin and Kumar (2016), copyright by Springer

Figure 54 from Badruddin and Kumar (2016) is a recent example of a large class of studies that use superposed epoch analysis (SEA) to combine observations for a large number of events with the aim of discerning trends and relationships between the GCR modulations and other parameters that may indicate the underlying physical processes. Both panels show at the top, the SEA results for the GCR intensity represented by the percentage change in the counting rates of the Oulu and Newark neutron monitors, for modulations associated with interaction regions with (black) or without (grey) forward shocks. Other data shown are the solar wind speed, magnetic field intensity (here denoted by *F*), the standard deviation of the magnetic field vector, and *FV* and $FV^2$. An important choice in SEA is the “zero epoch”, the feature that is used to line-up the observations of different events. In the left panel, the stream interface has been used, while the right uses the trailing edge of the interaction region. The choice clearly influences the profiles obtained, in particular for the solar wind parameters. In both cases, the GCR depressions are evidently weaker when no forward shock is present. The left-hand panel suggests that typically, the modulation commences ahead of the interface, but there is also a significant step down at the interface, where the solar wind speed also increases and the magnetic field peaks. In the right-hand panel, the modulation commences well ahead of the trailing edge of the interaction region and reaches maximum depression closely following the interaction region.

**Fig. 55:**
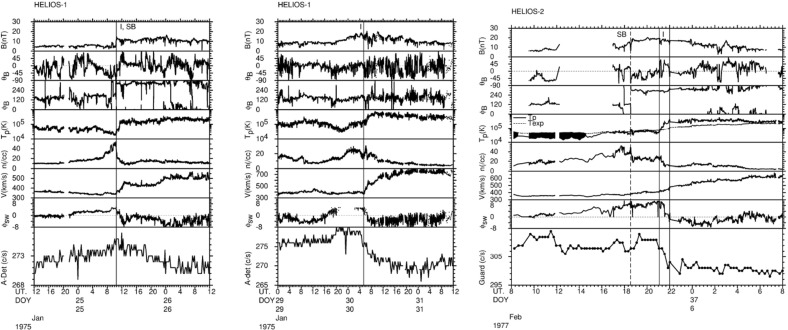
Examples of GCR depression onsets associated with interaction regions observed by the Helios spacecraft. ‘I’ indicates the stream interface, and ‘SB’ a sector boundary crossing. The GCR intensity is given by 15 min averages of the anti-coincidence guard of the University of Kiel instruments. Note that the depressions tend to commence in the vicinity of the stream interface/leading edge of the high-speed stream, and are not clearly associated with enhanced fields in the interaction region or sector boundaries (absent in the center panel). Image reproduced with permission from Richardson (2004), copyright by Kluwer

Although such analyses may provide insight into the causes of recurrent modulation, they also suppress information on event to event variations and the detailed relationship between the modulations and solar wind structure, which can also be valuable to study. For example, Fig. 55 from Richardson (2004) shows the onsets of three different recurrent modulations observed by the Helios spacecraft. Here the GCR observations are 15 min averages of the count rates of the anti-coincidence guards of the University of Kiel instruments on Helios. The left-hand event shows the GCR intensity peaking close to the stream interface, which is also nearly coincident with a sector boundary/HCS crossing and an abrupt increase in the magnetic field intensity, before declining in the high-speed stream. The center event shows the GCR intensity having a broad peak ahead of the interface within the interaction region and in the interval of increasing magnetic field strength, before declining relatively abruptly in the vicinity of the interface. The increase in the variability of the field and also solar wind direction (see $\phi _{sw}$) after the interface is clearly evident. In this case, there was no sector boundary in the vicinity of the modulation onset, nor at any other time during the interval shown. The right-hand event is expanded to show in detail the relationship between the GCR modulation and other structures. This clearly commences in the vicinity of the stream interface, which here extends over a period of around an hour, and is unrelated to the sector boundary around 3 h earlier or the enhanced magnetic fields associated with the interaction region.

**Fig. 56:**
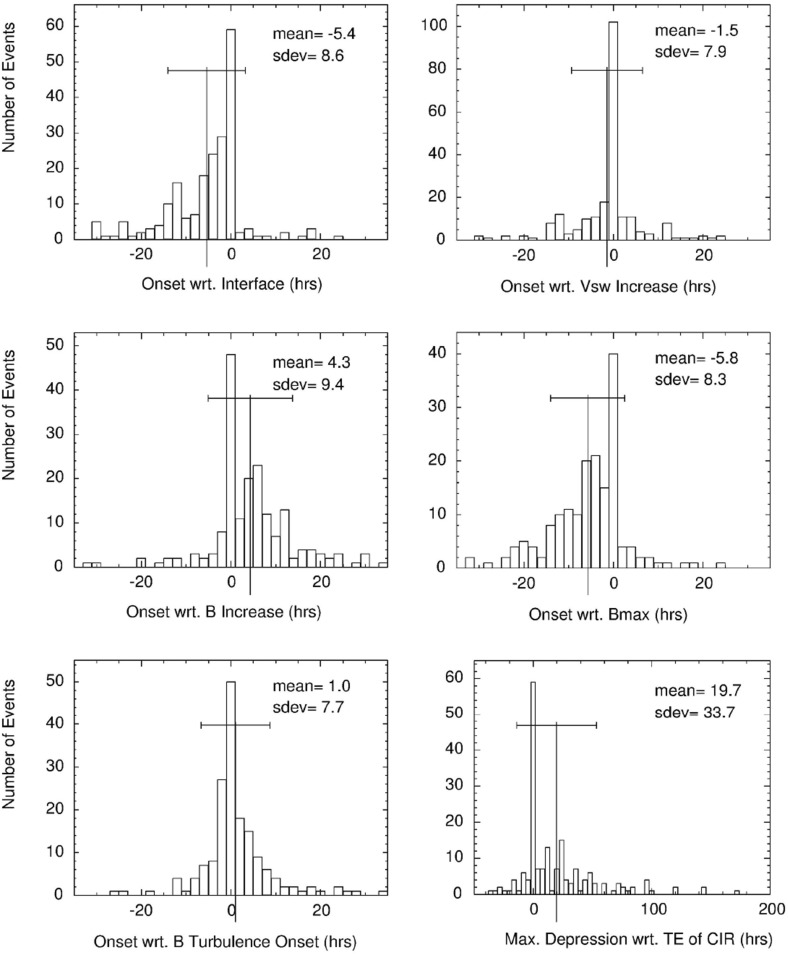
Summary of the onset times of Helios 1 or 2 or IMP 8 cosmic ray depressions with respect to various solar wind structures in the vicinity of interaction regions. A negative (positive) time indicates that the onset started ahead of (after) the solar wind structure. The bottom right panel shows the time of maximum particle depression relative to the trailing edge of the interaction region. Image reproduced with permission from Richardson (2004), copyright by Kluwer

Based on such observations at the Helios spacecraft and IMP 8, each panel in Fig. 56 from Richardson (2004) (except the bottom right) shows distributions of the time of the GCR depression onset relative to a specific solar wind feature, in particular the stream interface, increase in solar wind speed at the stream leading edge, the beginning and maximum of the interaction region-associated magnetic field enhancement, and the onset of enhanced magnetic field turbulence as indicated by the field component variances. A positive (negative) time indicates that the onset commenced after (or before) the solar wind feature. Overall, the particle depression onsets appear to be best ordered around the increase in solar wind speed at the stream leading edge (top-right panel) and the onset of field turbulence (bottom-left panel). The distribution in the top-left panel indicates that the depression onset frequently occurs in the vicinity of the stream interface (which also often coincides with the high-speed stream leading edge) while the asymmetry in this distribution indicates that some depressions commence ahead of the interface whereas those commencing after the interface are relatively rare. Richardson et al. (1996) noted that even if the depression does commence ahead of the interface, there is usually an additional relatively abrupt decrease in the vicinity of the interface, as also suggested by the SEA results in the left-hand panel of Fig. 54.

The middle-left panel in Fig. 56 shows the time of the depression onset relative to the start of the magnetic field enhancement associated with the interaction region. Although these features coincided to within 3 h in $\sim 40\%$ of events, the distribution is broad. The asymmetry arises because depressions tend to start at, or following, the start of the field enhancement associated with the interaction region (in particular in the vicinity of the stream interface/leading edge in the middle of the interaction region). The middle-right distribution shows the onset time relative to the time of maximum magnetic field in the vicinity of the interaction region. There is a peak at the time of field maximum but overall, the distribution is asymmetric with events commencing ahead of the field maximum considerably outnumbering those commencing afterwards. Overall, the increase in solar wind speed, which is frequently co-located with the stream interface and the onset of enhanced field turbulence, and may be accompanied by the start of a magnetic field intensity enhancement or maximum magnetic fields, organizes the onset times of corotating events reasonably well. However, there is evidently some event to event variation. Similarly, the conclusions from SEA and other studies are varied. For example, modulation onset has been found to occur at the increase in solar wind speed at the stream leading edge (e.g., Iucci et al. 1979), sector boundaries (e.g., Fujimoto et al. 1981; Badruddin et al. 1985), magnetic field enhancements (e.g., Murayama et al. 1979) and stream interfaces (e.g., Tiwari et al. 1983)

The bottom right histogram in Fig. 56 shows the time of maximum depression relative to the trailing edge of the interaction region (F’–F region boundary), where this can be inferred. The most likely location for the maximum depression ($\sim 30\%$ of events) is at the trailing edge of the interaction region (consistent with the SEA results in the right-hand panel of Fig. 54), though this can also lie several tens of hours after the interaction region in the high-speed stream. For 87% of the events, the maximum depression occurs at or following the trailing edge of the interaction region. Thus, the depression maximum occurs relatively infrequently inside the interaction region.

**Fig. 57:**
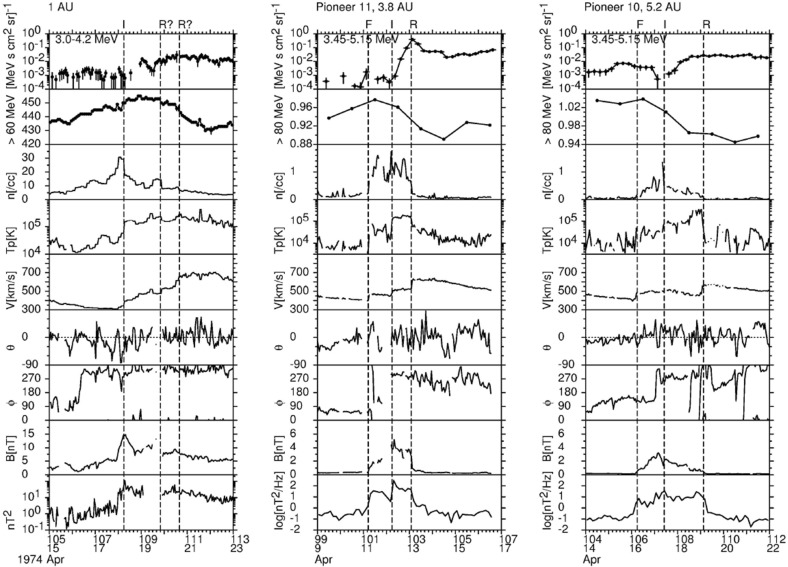
Observations of energetic particles, solar wind plasma and magnetic field (in HSE co-ordinates) associated with passage of the same interaction region at 1 AU, Pioneer 11 at 3.8 AU, and Pioneer 10 at 5.2 AU. Forward (F) and reverse (R) shocks and stream interface crossings (I) are indicated. The bottom panels indicate magnetic field turbulence levels. The GCR modulation associated with the interaction region is shown in the second graph in each panel

Figure 57 shows the onset of the cosmic ray modulation associated with the same interaction region observed at 1 AU (left), by Pioneer 11 at 3.8 AU (center), and by Pioneer 10 at 5.2 AU (right) (Richardson 2004). At 1 AU, this particular onset is unusual (in view of the previous discussion) because the modulation onset (observed by the IMP 8 GME guard) commences around a day after the stream interface (I) is crossed and occurs predominantly in the high-speed stream after the interaction region. It is evidently unrelated to the sector boundary crossing on April 16 ahead of the interaction region or the conspicuous local magnetic field enhancement encompassing the interface. At Pioneer 11, the interaction region is bounded by developed forward (F) and reverse (R) shocks, while the interface can be identified near the middle of the interaction region. The sector boundary, crossed during a data gap immediately before the interface, is now subsumed into the interaction region. The cosmic ray intensity is provided by $>80$ MeV proton observations from the University of Iowa instrument but low counting statistics and consequent time averaging prevent the precise relationship between the cosmic ray modulation and the interaction region structure from being established. Nevertheless, as at 1 AU, the cosmic ray intensity appears to reach a maximum in the vicinity of the interaction region before decreasing, most rapidly in the trailing part of the interaction region and high-speed stream. At Pioneer 10, the forward and reverse shocks and interface can again be identified, and the sector boundary crossing also precedes the interface. Again, the modulation onset (observed by the University of Iowa instrument) occurs within the interaction region, probably, within the limitations of the data, predominantly in the trailing part of the interaction region. Note that the highest intensity average is inside the leading part of the interaction region.

The bottom graph in each panel of Fig. 57 shows how the distribution of magnetic field turbulence levels within the interaction region evolves with increasing heliocentric distance. At 1 AU, the field turbulence (measured by the sum of the squares of the magnetic field component variances) increases in the vicinity of the stream interface and remains enhanced into the high-speed stream. At Pioneer 11, turbulence (represented by the power at wave numbers of 1.6–$3.3\times 10^{-5}$ km$^{-1}$ obtained from an Elsässer variable analysis; see Horbury and Schmidt (1999) for further details and discussion of the radial evolution of turbulence in interaction regions) increases first at the interaction region leading edge, but is further enhanced following the stream interface before declining in the high-speed stream. At Pioneer 10, turbulence again commences at the interaction region leading edge, but is more uniform within the interaction region, without an abrupt increase at the stream interface. Although, as discussed above, an association between enhanced turbulence and depressed cosmic ray intensity might be expected, these observations do not appear to be completely consistent with such a scenario. The topmost plots of Fig. 57 show MeV proton intensities that, as discussed in Sect. 7.1 show a tendency for enhancements to develop in the vicinity of the forward and reverse shocks beyond 1 AU. Note how the interface tends to lie at the leading edge of the “reverse shock” enhancement, as is typical (Intriligator and Siscoe 1994).

**Fig. 58:**
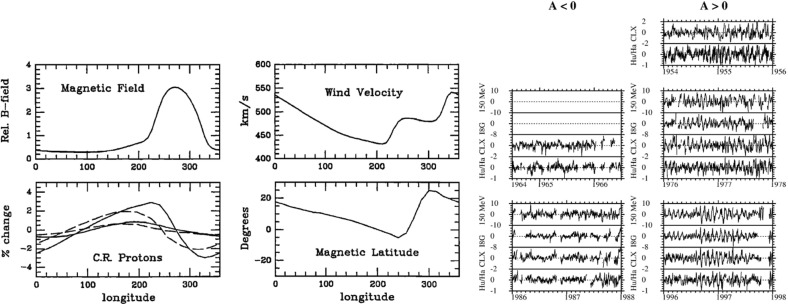
Left panels: parameters used to introduce an interaction region into the model of Kóta and Jokipii (1991) plotted versus solar longitude. The lower left panel shows the modeled modulation for $A>0$ (dashed lines) and $A<0$ solid lines assuming that the change in the diffusion coefficient associated with the magnetic field increase in the interaction region is turned on or off (weak modulation). Note that the modulation when $A<0$ is predicted to be larger than when $A>0$. Right panels: percentage variations in the cosmic ray intensity for 2-year periods around five solar minima, detrended to remove long-term variations. The data shown (where available) are 121–230 MeV (‘150 MeV’) proton and anti-coincidence guard observations (I8G) from the GME instrument on IMP 8, and observations from the Climax (CLX) and Huancayo or Haleakala (Hu/Ha) neutron monitors The recurrent $\sim$solar-rotation period variations are larger during $A>0$ epochs (right-hand panels) than when $A<0$ in all these data sets. Images reproduced with permission from [left] Kóta and Jokipii (1991), and from [right] Richardson et al. (1999), copyright by AGU

Considering modeling of recurrent GCR modulations, the left-hand panels of Fig. 58 show the parameters used to introduce an interaction region into the modulation model of Kóta and Jokipii (1991) plotted versus heliographic longitude (essentially time for a spacecraft “flying through” the simulation). The diffusion coefficient is assumed to be inversely proportional to B, i.e., particle diffusion is inhibited in stronger magnetic fields. The curves in the bottom left panel show the modeled 2 GeV proton intensity and also the weaker variations that result when the reduction in the diffusion coefficient associated with the interaction region is “turned off”. The line type indicates the direction of the global solar magnetic field (*A*). Specifically, the modulation is larger when $A<0$ (the solar field is inward at the north pole; solid line) than when $A>0$ (outward field at the north pole; dashed line). Briefly, the reason is that when $A>0$, positively charged particles drift in the heliospheric magnetic field inward at high latitudes and out along the current sheet at low latitudes, whereas when $A<0$, they drift inward along the current sheet and out at high latitudes (Jokipii et al. 1977). Hence, since interaction regions form at low latitudes, they are expected to be more efficient at modulating the incoming cosmic ray intensity when $A<0$. The model results led Kóta and Jokipii (1991) to conclude that the change in the diffusion coefficient related to the magnetic field increase is the most important parameter controlling the modulation process. However, it is also evident that the model predicts an intimate association between the magnetic field enhancement and the modulation that is not as observed. Furthermore, observationally, the amplitude of recurrent GCR modulations appears to be larger in solar minima when $A>0$, not $A<0$, as shown in the right-hand panels of Fig. 58, an effect (Richardson et al. 1999) that may be related to *A*-epoch dependencies in the particle diffusion coefficients (Chen and Bieber 1993; Wibberenz et al. 1998). Aspects of the *A* dependence of recurrent GCR modulations have also been discussed by, e.g., Alania et al. (2011), Modzelewska and Alania (2012), Thomas et al. (2014) and Gil and Mursula (2017).

**Fig. 59:**
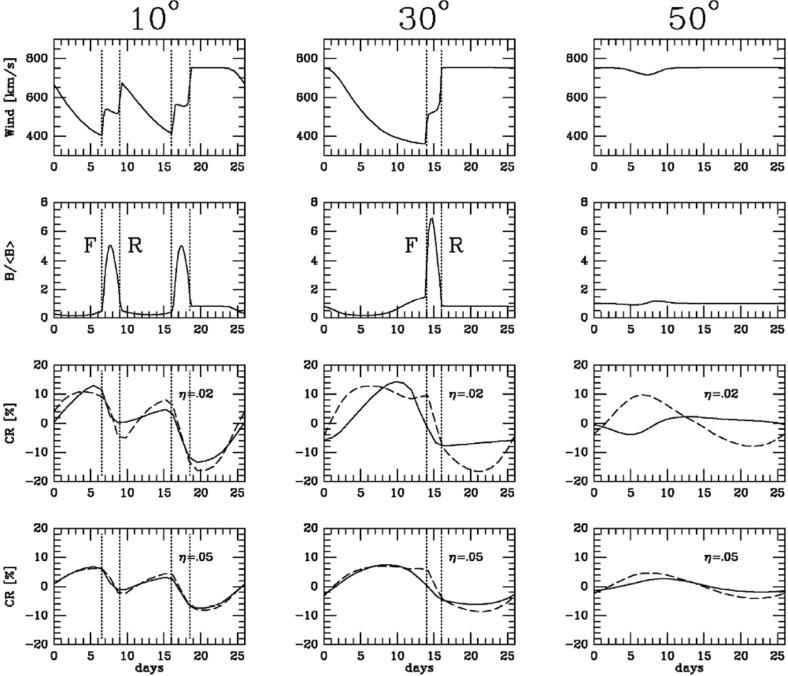
Variations in the solar wind speed, magnetic field intensity, and cosmic ray proton intensity at 3 AU and $10^\circ$, $30^\circ$ and $50^\circ$ heliolatitude obtained from a 3-D simulation by Kóta and Jokipii in McKibben et al. (1999). Solid and dashed lines indicate results for $A > 0$ and $A < 0$ epochs, respectively (note this convention is reversed from Fig. 58), for two values of the perpendicular/parallel diffusion coefficient ratio $\eta$. F (R) $=$ forward (reverse) shock. Image reproduced with permission from McKibben et al. (1999), copyright by Kluwer

Ulysses observations of GCR modulations to high latitudes have confronted modelers with similar issues to those involved in explaining how energetic ions accelerated at interaction regions also reach high latitudes. For example, maybe a non-Parker field could connect interaction regions at low latitudes to higher latitudes, or perpendicular particle diffusion might cause the modulation effects in the vicinity of interaction regions to extend to higher latitudes? Fig. 59 shows results of a model by Kóta and Jokipii (McKibben et al. 1999) of GCR modulation at 3 AU and at $10^\circ$, $30^\circ$ and $50^\circ$ heliolatitude. Again, larger modulations are (incorrectly) predicted when $A<0$ (note that the solid-dashed line convention is reversed here compared to Fig. 58), and the diffusion coefficient is assumed to be proportional to 1 / *B*. Two values of the ratio of the perpendicular to parallel diffusion coefficients ($\eta =0.02$ and 0.05) are assumed that are sufficient to allow the modulations to extend to at least $50^\circ$, where there is only a slight remnant of the interaction region that is evident at lower latitudes.

**Fig. 60:**
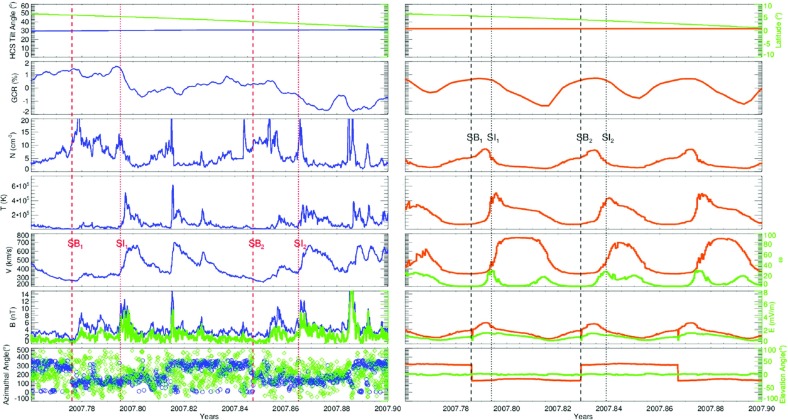
Solar wind and GCR observations (left) near Earth during a period in 2007, and corresponding modeled parameters. Note that the GCR modulations are unrelated to enhancements in the magnetic field intensity and sector boundary crossings, and commence in the vicinity of stream interfaces/fast stream leading edges, consistent with the observations in the left panel and discussed in the text. See Guo and Florinski (2016) for a detailed description of the parameters shown in the figure. Image reproduced with permission from Guo and Florinski (2016), copyright by AAS

As an example of more recent modeling, Fig. 60 from Guo and Florinski (2016) shows in the right panel modeled solar wind parameters and GCR modulations compared with the original data for a period in 2007 in the left panel, where ‘SB’ and ‘SI’ indicate a sector boundary/current sheet crossing and a stream interface crossing, respectively. [Note, however, that the lines indicating the interfaces in the left panel are incorrectly placed (Guo and Florinski, private communication, 2017).] The model, which does not scale the diffusion coefficient with B, but uses different values in slow and fast solar wind, captures two features of the observations evident in the left panel and also discussed above. First, the GCR modulations (second graph from top) are relatively independent of the magnetic field intensity and commence in the vicinity of the interface/stream leading edges, not at the magnetic field enhancements associated with the interaction region. Second, the GCR intensity variations are unrelated to the sector boundary/current sheet crossings. On the other hand, the tendency for the GCR intensity in the model to reach a minimum towards the trailing edge of the high-speed streams appears to be inconsistent with observations (e.g., Fig. 53) that indicate that the modulations are often deepest near the stream leading edge, then recover during stream passage. Nevertheless, such work exemplifies the progress that is being made in understanding recurrent GCR modulations, but that uncertainties still remain.

## Geomagnetic activity associated with stream interaction regions

As discussed in Sect. 2, stream interaction regions and the associated high-speed streams tend to enhance geomagnetic activity when they sweep past the Earth, and the resulting recurrent activity was an important early indicator of the influence of the Sun on the Earth’s environment. Furthermore, it was established from pioneering solar wind observations that the level of geomagnetic activity is correlated with the solar wind speed. However, Crooker (2000) notes that “a common misunderstanding about high-speed streams is that the high-speed flow itself causes geomagnetic storms”. Enhanced geomagnetic activity is a consequence of an increase in the rate of energy transfer from the solar wind into the Earth’s magnetosphere. This is largely determined by the strength and orientation of the interplanetary magnetic field, and the solar wind speed and density. One formulation (not including density) is the $\epsilon$ function of Perreault and Akasofu (1978), $\epsilon =l^2_oVB^2\sin ^4(\theta /2)$, where $l^2_o$ is the area of the magnetopause through which the energy enters, and $\theta$ is the “clock angle” of the IMF relative to the Sun–Earth line. (See Newell et al. 2007 for further discussion of solar wind-magnetospheric coupling functions.) Although increased energy transfer is expected in faster solar wind, the typical factor of $\sim 2$–3 variation in solar wind speed is much less than the variation in the magnetic field dependence, in particular arising from changes in the field orientation. The most efficient energy transfer occurs when the IMF has a southward component ($\theta =180^\circ$ for a southward clock angle), facilitating reconnection between the solar wind and magnetospheric magnetic fields (Dungey 1961).

**Fig. 61:**
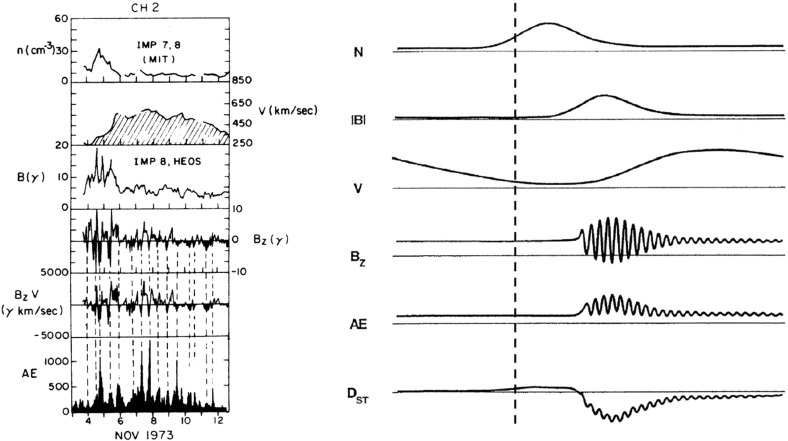
Left: An interaction region and high-speed stream in November 1973 showing the close relationship between bursts of geomagnetic activity indicated by the AE index and brief southward turnings (negative $B_z$) throughout passage of the stream. The extended activity is an example of “High Intensity Long Duration Continuous AE Activity” (HILDCAA, Tsurutani and Gonzalez 1987; Tsurutani et al. 1990) also illustrated schematically in the right-hand figure, where the behavior of the *Dst* geomagnetic index is also indicated. The vertical dashed line indicates a hypothetical location for the heliospheric current sheet. Images reproduced with permission from [left] Burlaga and Lepping (1977), copyright by Elsevier; and [right] from Tsurutani et al. (2006a, b), copyright by AGU

Burlaga and Lepping (1977) were among the first to examine in detail the interplanetary causes of geomagnetic activity associated with interaction regions and high-speed streams. The left panel in Fig. 61 shows one event from their study. In addition to the solar wind density, speed and magnetic field intensity, which clearly show the interaction region and high-speed stream, the figure includes the north–south component of the magnetic field ($B_z$), the *y*-component of the interplanetary electric field ($\sim B_zV$), and the geomagnetic AE index (Davis and Sugiura 1966), which measures auroral zone activity. Burlaga and Lepping (1977) noted a “striking correlation” between the bursts in AE and large southward (negative) values of $B_z$, concluding that “$B_z$ *is an essential factor in causing the geomagnetic activity*”. The burst-like nature of AE results from the highly variable magnetic field on time scales of a few hours throughout the passage of the high-speed stream that is predominantly related to large-amplitude Alfvénic fluctuations (cf. Fig. 16) moving outward from the Sun (e.g., Belcher and Davis 1971; Smith et al. 1995; Tsurutani et al. 1995). Burlaga and Lepping (1977) also noted that geomagnetic activity tends to be stronger in the vicinity of the interaction region-associated magnetic field enhancement, where compression would be expected to enhance any southward fields present, a scenario supported by Ulysses results (Tsurutani et al. 1995). A schematic of this process is shown in the right panel of Fig. 61 from Tsurutani et al. (2006a), Tsurutani et al. (2006b), which also indicates the different response in the AE auroral zone index and in *Dst* (http://wdc.kugi.kyoto-u.ac.jp/dstdir/dst2/onDstindex.html), which is a mid-latitude index measuring the strength of the ring current that is often used as a measure of geomagnetic storm size. The right-hand panel of Fig. 61 also illustrates the concept of “High Intensity Long Duration Continuous AE Activity” (HILDCAA, Tsurutani and Gonzalez 1987; Tsurutani et al. 1990), the extended activity in AE driven by southward magnetic field fluctuations associated with Alfvén waves extending throughout the high-speed stream, which is also evident in the observations in the left-hand panel of this figure, where activity persists for several days. Since the interaction region is the driver of the most intense recurrent activity, this led Crooker and Cliver (1994) to propound a “post-modern view of M-regions” in which the source at the Sun not only consists of a high-speed stream from a coronal hole but also the slower solar wind from the streamer belt that is necessary for the formation of the interaction region. Crooker and Cliver (1994) also noted that interplanetary coronal mass ejections propagating in the streamer belt ahead of streams may contribute to recurrent activity, as discussed further below.

**Fig. 62:**
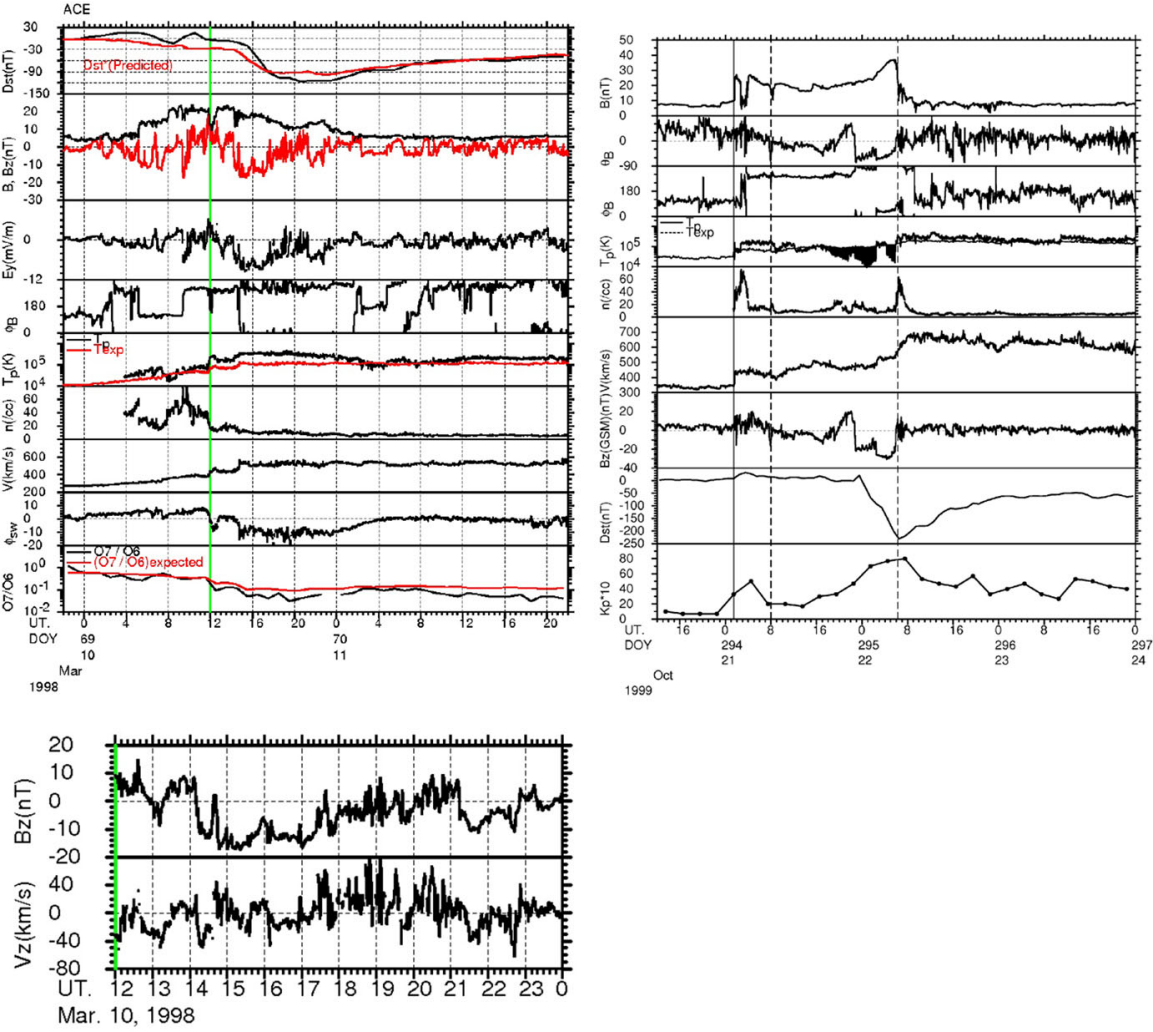
Top left: An example of an interaction region that gave rise to an “intense” ($Dst \le -100$ nT) storm (top graph) associated with southward magnetic fields (negative $B_z$) following the stream interface (vertical green line). Note that the *y* component of the solar wind electric field ($E_y$) is multiplied by $-1$ to track $B_z$. The bottom left panel shows the *z* components of the magnetic field and solar wind velocity during 12 h after the interface. The correlated variations are evidence of Alfvénic fluctuations. The right panel shows an example where an ICME (delineated by the dashed vertical lines) is present in the interaction region. Compression of the trailing part of the ICME by the following high-speed flow strengthens the southward fields inside the ICME and leads to a severe geomagnetic storm. Images reproduced with permission from [left] Richardson et al. (2006), copyright by AGU; and [right] Richardson (2006), copyright by AGU; see also Dal Lago et al. (2006)

The top-left panel of Fig. 62 shows an example of a geomagnetic storm associated with passage of an interaction region (Richardson et al. 2006), which exceeded the $Dst= -100$ nT threshold (top graph) for an “intense storm” (e.g., Tsurutani and Gonzalez 1997). The vertical green line indicates the stream interface. In this case, the storm was evidently driven predominantly by enhanced southward magnetic fields and solar wind electric field $E_y$ (here multiplied by $-1$ to track $B_z$) following the interface. The red curve in the top panel is the predicted *Dst* using the O’Brien and McPherron (2000) formula linking *Dst* with solar wind conditions, which is reasonably consistent with the observed *Dst*. The bottom-left panel shows the north–south components of the magnetic field and solar wind velocity for a 12 h interval following the stream interface. Clear correlations between these parameters, apparent by eye, are evidence of Alfvénic fluctuations in the region that generates the storm (cf. Fig. 16).

Examining intense storms with $Dst \le -100$ nT, Zhang et al. (2007) (see also Echer et al. 2008) concluded that 11 (13%) of the 88 intense storms in 1996–2005, during solar cycle 23 were associated with stream interaction regions [the remainder were associated with interplanetary coronal mass ejections (ICMEs)]. These storms and the related interaction regions are discussed in more detail by Richardson et al. (2006) (see also Alves et al. 2006). The largest of these storms had minimum $Dst= -128$ nT. Examining similar storms in 1972–1995, the strongest storm identified had minimum $Dst= -161$ nT. The left-hand panel of Fig. 63 from Richardson et al. (2006) compares the distributions of minimum *Dst* during the passage of 159 interaction regions and 281 ICMEs (Richardson and Cane 2010) in 1996–2005. In both cases, the distributions peak at $Dst\sim -40$ nT, but the ICME distribution clearly has a tail of severe storms, which is not present for interaction regions. As discussed by Richardson et al. (2006), the maximum size of an interaction region-associated storm is likely to be limited by the strength of the southward magnetic field component, which in turn is limited by the field enhancement generated by the compression associated with the stream interaction, and by the solar wind speed, which, in the interaction region between slow and fast solar wind, will never reach the high speeds (occasionally 1000 km s$^{-1}$ or more) associated with some ICMEs. Richardson et al. (2006) suggest that with maximum southward fields rarely exceeding $\sim 20$ nT and speeds of $\sim 450$ km s$^{-1}$ in the interaction region, then using the O’Brien and McPherron (2000) formula, *Dst* would be rarely expected to exceed $\sim -180$ nT, consistent with the strongest storm identified in their survey back to 1972.

A circumstance that may give rise to a stronger storm is when an ICME becomes incorporated into the interaction region. An example is illustrated in the right-hand panel of Fig. 62 where the trailing edge of the ICME (delineated by the vertical dashed lines; the solid line indicates a forward shock generated by the motion of the ICME) is compressed by the following high-speed solar wind, enhancing the southward fields inside the trailing edge of the ICME and generating a storm that reached $Dst=-237$ nT. Zhang et al. (2007) identify this (see also Dal Lago et al. 2006) and two other cases in their study where intense storms were generated by an interaction between an ICME and a corotating high-speed stream in their sample of 88 intense storms. Note these are not included in the “interaction region associated” storms discussed here.

**Fig. 63:**
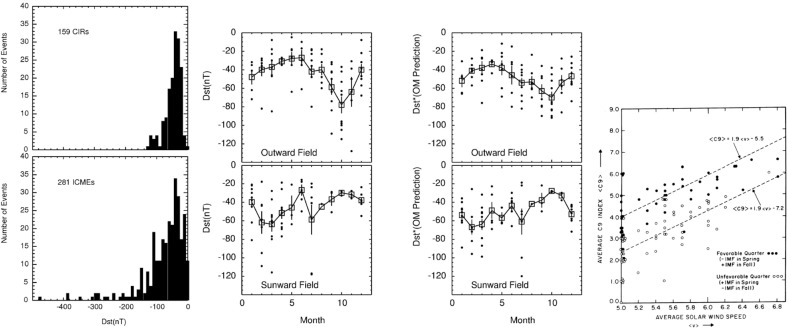
Left: Histograms of minimum *Dst* values associated with 159 interaction regions and 281 ICMEs in 1996–2005. Both peak at $Dst\sim -40$ nT but the interaction region distribution lacks the high intensity tail evident for ICMEs. The center panels show the seasonal variation in interaction region-associated geomagnetic activity observed (left center) and predicted by the O’Brien and McPherron (2000) formula (right center) for cases where the magnetic fields in the activity driver are directed away (top) or toward (bottom) the Sun. Monthly averages are also indicated. Right: Average C9 geomagnetic index and solar wind speed in a group of high-speed streams observed in spring or fall, showing higher activity levels in similar speed streams with favorably-directed magnetic fields. Images reproduced with permission from [left, center] Richardson et al. (2006), copyright by AGU; and from [right] Sheeley et al. (1977), copyright by D. Reidel

The center-left panels of Fig. 63 from Richardson et al. (2006) show the sizes of storms associated with interaction regions discussed above in 1996–2005 plotted versus month of observation, with the events divided according to the sunward (inward) or outward direction of the solar wind magnetic field in the region driving the storm. There is a clear seasonal effect (squares show averages for each month), with, for outward fields, larger storms tending to occur around the spring equinox and weaker around the autumn equinox, and the reverse pattern for sunward (inward) fields. The center-right panels show that this same pattern is evident in *Dst* for each event predicted by the O’Brien and McPherron (2000) formula, indicating that the seasonal variation is driven by the solar wind–magnetosphere coupling. The pattern is consistent with the Russell and McPherron (1973) effect, but other factors may also contribute (e.g., Cliver et al. 2000; O’Brien and McPherron 2000). The right-hand panel of Fig. 63 (Sheeley et al. 1977) shows the seasonal variation in another way, by plotting the average C9 index versus solar wind speed for two groups of streams separated by whether the magnetic field direction was favorable for storm production in a given season (solid circles) or unfavorable (open circles) based on the Russell and McPherron (1973) effect.

**Fig. 64:**
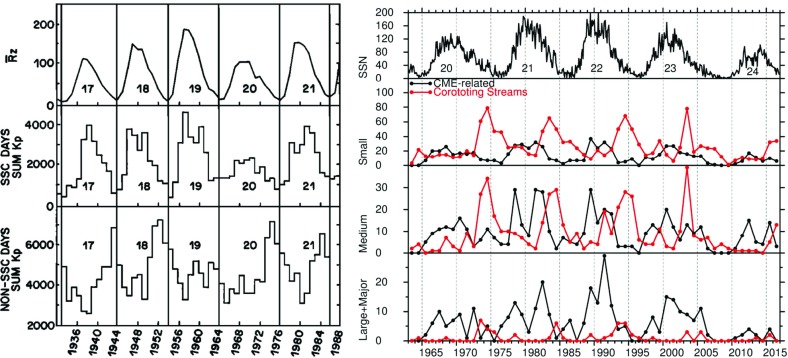
Left: The sunspot number for 1932–1988 together with the annual sums of *Kp* for “SSC days”, defined as up to two days following a geomagnetic storm sudden commencement, and days not related to an SSC. Image reproduced with permission from Venkatesan et al. (1991), copyright by AGU. Right: Annual numbers of storms of various sizes (see text for details) associated with transients (black) and corotating streams (red) in 1964–2016, updated from Richardson et al. (2001), Richardson (2006), Richardson and Cane (2012), showing the tendency for stream-associated storms to peak in the declining phase of the cycle, in contrast with the solar cycle dependence of transient-associated storms. The distributions of *Kp* for non-SSC and SSC days, respectively, in the left-hand panel follow similar time variations

Figure 64 shows examples of studies that aim to distinguish between activity associated with transient structures (i.e., shocks and ICMEs) and interaction regions/high-speed streams, and show their different variations through the solar cycle. Considering first the right-hand panel, this shows the numbers of geomagnetic storms of various sizes during 1964–2016 associated with these structures, updated from Richardson et al. (2001), Richardson (2006), and Richardson and Cane (2012). The storms are identified using the *Kp* index (Menvielle and Berthelier 1991) with the storm strengths defined following Gosling et al. (1991): “Major”: $Kp_\mathrm{max} \ge 8$ and $Kp \ge 6$ for at least three 3-h intervals in a 24-h period; “Large”: $7 \le Kp_\mathrm{max} \le 7+$, and $Kp \ge 6$ for at least three 3-h intervals in a 24-h period; “Medium”: all other cases with $Kp_\mathrm{max} \ge 6-$; “Small”: $5- \le Kp_\mathrm{max} \le 5+$. Note that these criteria identify “storm days”, so a storm extending over several days may contribute to more than one day of storm conditions. See Richardson and Cane (2012) for more information on the identification of these storms and the related solar wind structures, which is based on examining OMNI solar wind data and additional data. Stream-related storms (red graphs) occur predominantly in three–four year intervals during the decay of the solar cycle, while in contrast, CME associated storms (black graphs) follow the solar cycle, though often with a decrease in the rate near solar maximum (especially evident in cycle 21) related to the “Gnevyshev gap” (e.g., Gnevyshev 1967, 1977; Feminella and Storini 1997; Norton and Gallagher 2009), a temporary decrease in the occurrence of energetic solar activity near solar maximum associated with the reversal of the solar magnetic field. Weaker storms are increasingly more likely to be associated with interaction regions/high-speed streams.

Considering the left-hand panel in Fig. 64 from Venkatesan et al. (1991), *Kp* is summed for two groups, depending on whether or not a geomagnetic storm sudden commencement (typically associated with the arrival of an interplanetary shock, and assumed here to be indicative of a transient) occurs within the previous 2 days, during each year between 1932 and 1988. The SSC-associated and non-SSC associated summed *Kp* clearly follow similar temporal patterns relative to the solar cycle as the “CME-associated” and “corotating stream associated storms” in the right figure. Thus, taken together, the observations in Fig. 64 show the tendency for stream associated geomagnetic activity to occur in the descending phases of eight solar cycles. Note also the similarity of the results in Fig. 64 with those of Newton and Milsom (1954) in the right-hand panel of Fig. 6, where the decrease in storm occurrence near solar maximum associated with the Gnevyshev gap is also evident,

Interaction regions are also associated with other magnetospheric phenomena such as the acceleration of radiation belt electrons and irregularities in global positioning systems, but a detailed discussion is beyond the scope of this paper. An overview of this topic is given by Tsurutani et al. (2006b) (see also Tsurutani et al. 2006a) and other papers in the same AGU *Geophysical Monograph* (number 167).

## Observations of interaction regions by the STEREO spacecraft

The twin STEREO A and B spacecraft (Kaiser et al. 2008), launched on October 26, 2006 into heliocentric orbits at $\sim 1$ AU moving Ahead of (STEREO A) or Behind (STEREO B) the Earth at $\sim 22^\circ$/year, have provided a new multi-point perspective of stream interaction regions, including their temporal and spatial variations at different locations. Even when the spacecraft were still close together, significant differences were found. For example, Fig. 65 from Jian et al. (2009) shows an interaction region observed in May 2007 when STEREO A was leading STEREO B by only $7^\circ$ and was $1^\circ$ further north and 0.09 AU closer to the Sun. The solar wind speed profiles at both spacecraft are fairly similar, but other parameters show remarkable differences, including the more rapid increases in the temperature, entropy ($S=ln(T_p^{3/2} /N_p)$), and magnetic field at STEREO B following the vertical black line, which indicates the heliospheric current sheet crossing, than at STEREO A, where the vertical black line also indicates the HCS. In addition, the density shows a strong enhancement inside the interaction region leading edge at STEREO A that is not evident at STEREO B, suggesting that it is unrelated to the heliospheric plasma sheet since the HCS is observed at both spacecraft. The bottom panel shows the total (plasma and magnetic field) pressure perpendicular to the magnetic field ($P_t$), which also has different profiles at the two spacecraft, with a larger abrupt increase at the forward shock forming the leading edge of the interaction region at STEREO A than at STEREO B (vertical red dotted lines labeled ‘f.s.’ indicate the forward shocks). Note that Jian et al. (2009) define the stream interface at STEREO B (vertical purple line) using the peak in $P_t$ (Jian et al. 2006), and do not define one at STEREO A where there is no well-defined peak in $P_t$. The observations clearly indicate significant spatial variations may exist, even on relatively small scales, within interaction regions at $\sim 1$ AU.

**Fig. 65:**
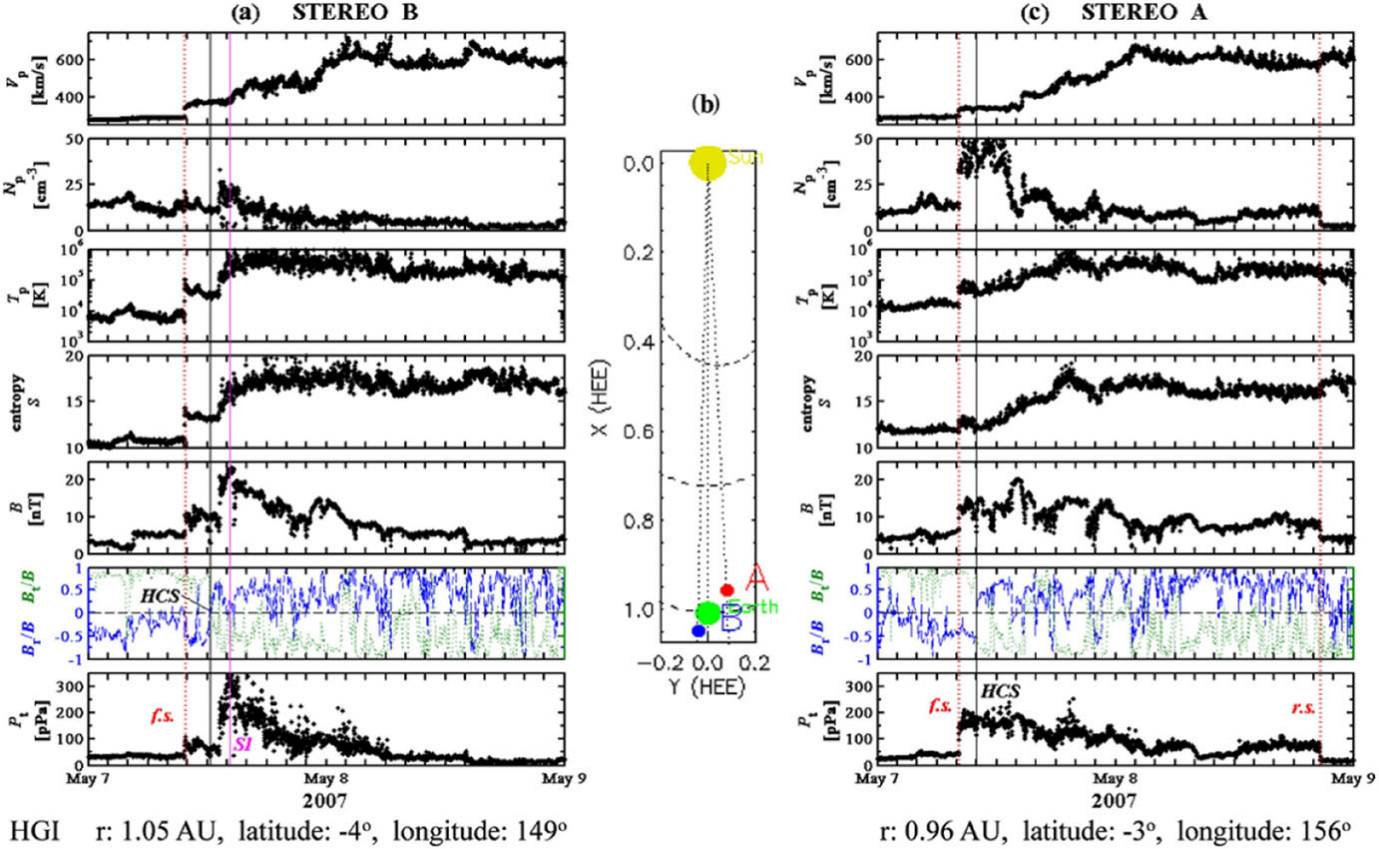
A stream interaction region observed at the STEREO B (left) and STEREO A spacecraft (right) on May 7–8, 2007 when the spacecraft were separated by only $7^\circ$ in longitude. The panels (with the same scale at each spacecraft) show the solar wind speed ($V_p$), proton number density ($N_p$), proton temperature ($T_p$), entropy ($S =ln(T_p^{3/2}/N_p$), magnetic field intensity (B), ratios of $B_r$ (blue dashed line) and $B_t$ (green dotted line) to B, and total perpendicular pressure ($P_t$). The vertical solid black and purple lines indicate the HCS and stream interface (SI, defined by the peak of $P_t$), respectively. Red dotted lines mark forward (f.s.) and reverse (r.s.) shocks. Image reproduced with permission from Jian et al. (2009), copyright by Springer

**Fig. 66:**
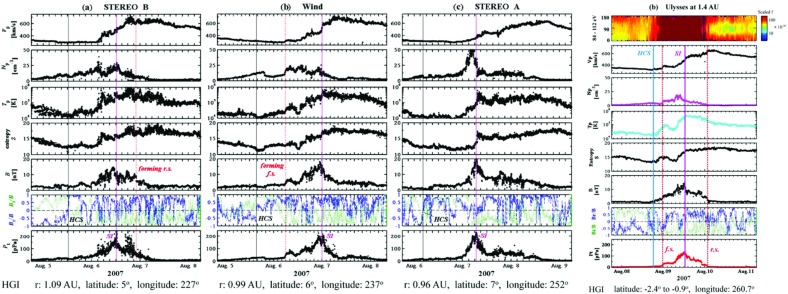
An interaction region in August 2007 observed by (left to right) STEREO B, WIND, STEREO A, and Ulysses. STEREO B was $10^\circ$ east of WIND, while STEREO A was $15^\circ$ to the west. Ulysses was at 1.4 AU and $9^\circ$ west of STEREO A near the ecliptic during a fast latitude scan. Again, significant differences can be seen in the solar wind parameters at each spacecraft. Image reproduced with permission from Jian et al. (2009), copyright by Springer

Figure 66 shows another interaction region that swept past STEREO B, $10^\circ$ east of WIND (near Earth) and then STEREO A, $15^\circ$ west of WIND, on August 6–7, 2007. The interaction region was also observed by Ulysses when $9^\circ$ west of STEREO A and at 1.4 AU near the ecliptic during a fast latitude scan. The Ulysses data in the right-hand panel also show suprathermal electron pitch angles relative to the magnetic field direction used by Jian et al. (2009) to help establish the true location of the heliospheric current sheet. In this case, the heliospheric current sheet was well ahead of the interaction region at all locations so the variations in the solar wind parameters are due to the interaction between the slow and fast solar wind. Again, there are significant differences in the profiles and the presence or absence of shocks at all the spacecraft near 1 AU, even though only separated by $25^\circ$ in longitude. At Ulysses, the interaction region boundaries have steepened into a forward and reverse shock pair. Such observations illustrate that although stream interaction regions can endure for many solar rotations, they are evidently not static structures but show considerable variation and evolution even when observed by spacecraft a day or two apart.

**Fig. 67:**
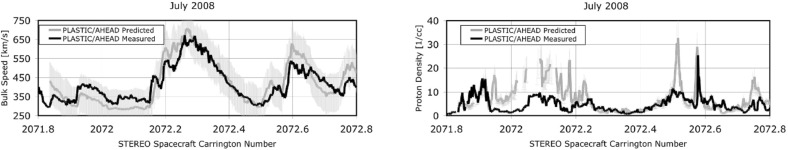
A test of the concept of using a spacecraft at L5 to predict the solar wind conditions at Earth, made using observations from STEREO B to predict the solar wind speed (left) and density (right) at STEREO A when the spacecraft were separated by $\sim 60^\circ$ in July 2008. Predicted and observed parameters at STEREO A are shown by grey and black curves, respectively. An uncertainty of $\pm 100$ km s$^{-1}$ on the predicted speed is shown with grey shading. Image reproduced with permission from Simunac et al. (2009b), copyright by the authors

The idea of positioning a spacecraft at the L5 libration point, $60^\circ$ east of Earth, which might monitor corotating streams $\sim 3$–5 days before they arrive at Earth, has been proposed (e.g., Akioka et al. 2005; Vourlidas 2015). In a test of such a scenario, Simunac et al. (2009b) discussed a period in July 2008, when the STEREO spacecraft were at a similar separation. The grey traces in Fig. 67 show the solar wind speed (left) or density (right) measured at STEREO B and then mapped assuming corotation to give a “predicted” profile at STEREO A. The black traces give the profiles actually observed at STEREO A (note that they are plotted vs. Carrington longitude to remove corotation). The solar wind speed is predicted reasonably well, but the density prediction is less successful. Nevertheless, Simunac et al. (2009b) conclude that an L5 monitor “would augment our space weather forecasting capabilities for the Earth”.

**Fig. 68:**
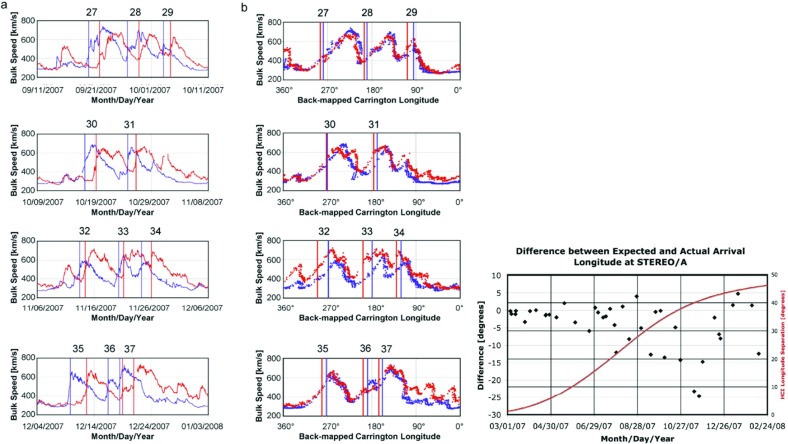
Left: Solar wind speeds at STEREO A (red) and B (blue) during four solar rotations in 2007. Stream interfaces are indicated by vertical lines and numbered as in Simunac et al. (2009a). These observations are mapped back to the Carrington longitude of their source location at the Sun in the center panels, where the usual convention for Carrington longitude scale has been reversed to be consistent with the sense of the time scale. Note that generally, the mapped speed profiles are fairly similar, but the stream interface arrival times are not always in agreement. The differences are larger on the third rotation where the source coronal hole configuration was evolving. The right plot shows the difference in interface arrival times and the spacecraft longitude separation (red graph) as functions of time, indicating the larger arrival time differences when the separation exceeds $\sim 20^\circ$. Images reproduced with permission from Simunac et al. (2009a), copyright by the authors

In another study, Simunac et al. (2009a) identified 41 stream interfaces observed at both STEREO spacecraft in March 2007 to February 2008, when the spacecraft were separated by $1^\circ$ to $46^\circ$ in longitude. The left-hand panels of Fig. 68 show the observed solar wind speeds at STEREO B (blue) and STEREO A (red) during four solar rotations in September–December, 2007 with the stream interfaces (numbered) indicated by vertical lines. In the center panels, the speeds are mapped to Carrington longitude at the Sun, to remove the corotation. Note that the longitude scale has been reversed from normal Carrington maps so that runs in the same sense as time in the left-hand panels. Simunac et al. (2009a) point out that the interface arrival times when plotted in this way do not usually agree precisely and in particular, the mapped STEREO B interface times tend to lag those for STEREO A. The right-hand panel shows how the lag (expressed in degrees longitude) tends to increase with time as the spacecraft separation also increases, in particular when the separation is $>20^\circ$. The third rotation shown in the center plots also shows large interface separations and more significant differences in the speed profiles at each spacecraft due to rapid evolution of the source coronal holes during this rotation.

**Fig. 69:**
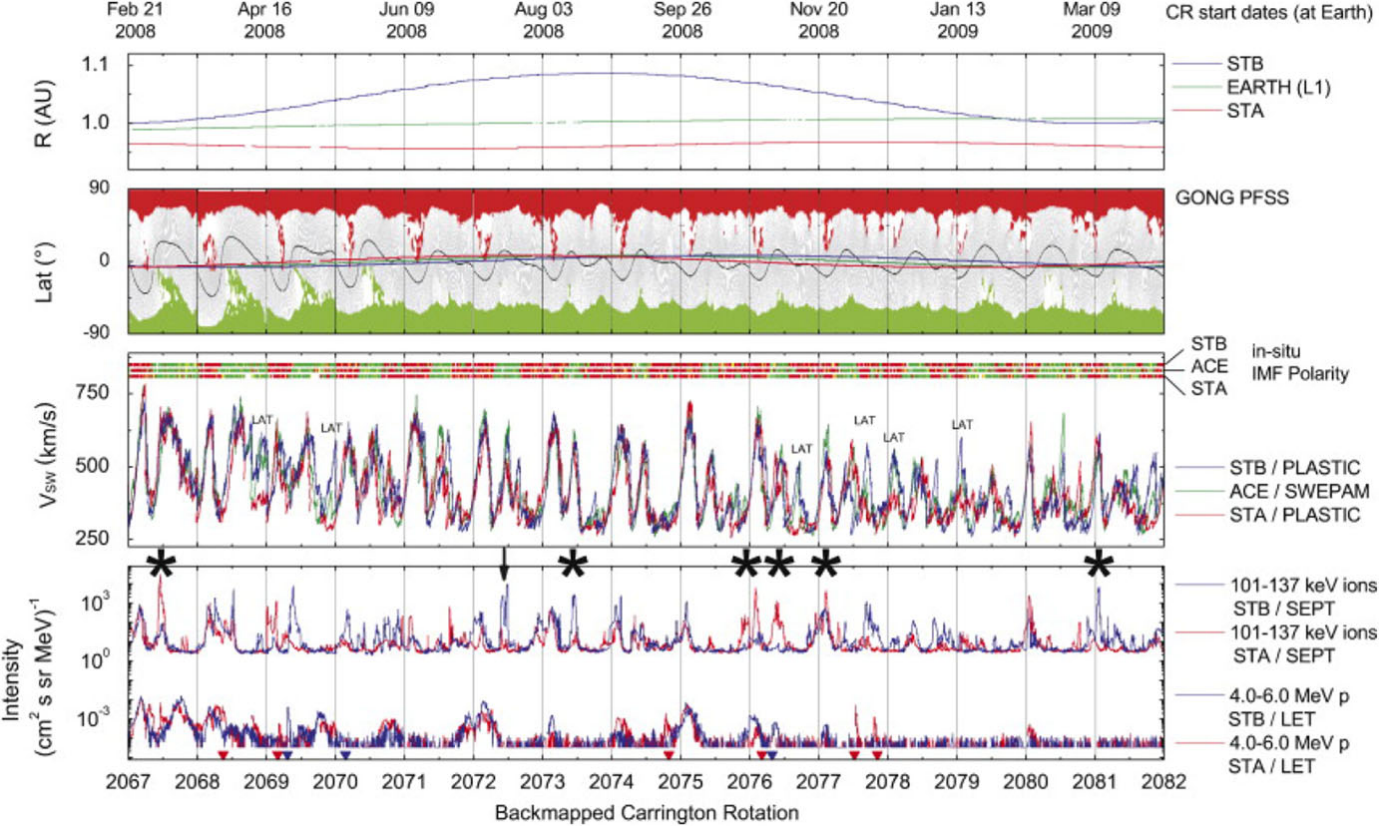
An interval from February 2008 to March 2009 showing the heliocentric distances of the STEREO spacecraft and ACE (at Earth; green), coronal holes and the location of the heliospheric plasma sheet obtained from GONG data using a potential field source surface model with the spacecraft latitudes superposed, the solar wind speed at all three spacecraft, and energetic particle intensities in two energy ranges at the STEREO spacecraft, backmapped to Carrington rotation. See text for further details. Image reproduced with permission from Gómez-Herrero et al. (2011), copyright by Elsevier

Figure 69 from Gómez-Herrero et al. (2011) summarizes observations for a longer interval (February 2008 to March 2009) that include, in the third panel from the top, the solar wind speed at the two STEREO spacecraft and at ACE (green) mapped against Carrington longitude as in Fig. 68. The other panels show the spacecraft heliocentric distances, negative (red) and positive (green) polarity coronal holes and the location of the heliospheric current sheet with the spacecraft latitudes superposed, the in situ magnetic field polarity at each spacecraft (in the solar wind speed panel), and the 101–137 keV ion and 4–6 MeV proton intensities at the STEREO spacecraft (events involving ICMEs are indicated by asterisks, and inverted diamonds indicate solar energetic particle events). The mapped back solar wind speed profiles are generally similar at the three spacecraft, but there are also differences, some of which may be related to differences in latitude between the spacecraft (indicated by “LAT”). Similarly, the particle intensities show enhancements that are similar at both spacecraft, and others that are not. Figure 70 from Gómez-Herrero et al. (2011) shows three sample interaction regions in more detail, where observations from the STEREO spacecraft have been time shifted to ACE to remove corotation. Again, there are clear variations in the solar wind profiles, including the presence of shocks and ICMEs (shaded intervals, colored according to spacecraft), and energetic particle profiles. Gómez-Herrero et al. (2011) note that the lower energy ion intensity tends to be enhanced in the vicinity of the interaction region, consistent with the occurrence of local particle acceleration as discussed in Sect. 7.1.

**Fig. 70:**
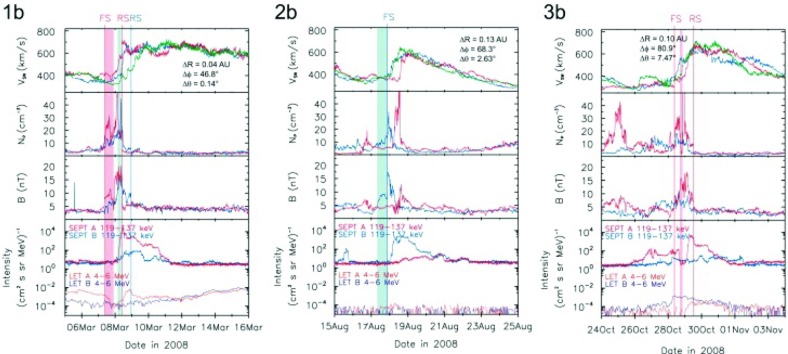
Three interaction regions showing solar wind and energetic particle observations at the STEREO spacecraft time shifted to the ACE spacecraft (Gómez-Herrero et al. 2011). Shaded intervals indicate the presence of ICMEs at the spacecraft indicated by the color. Image reproduced with permission from Gómez-Herrero et al. (2011), copyright by Elsevier

**Fig. 71:**
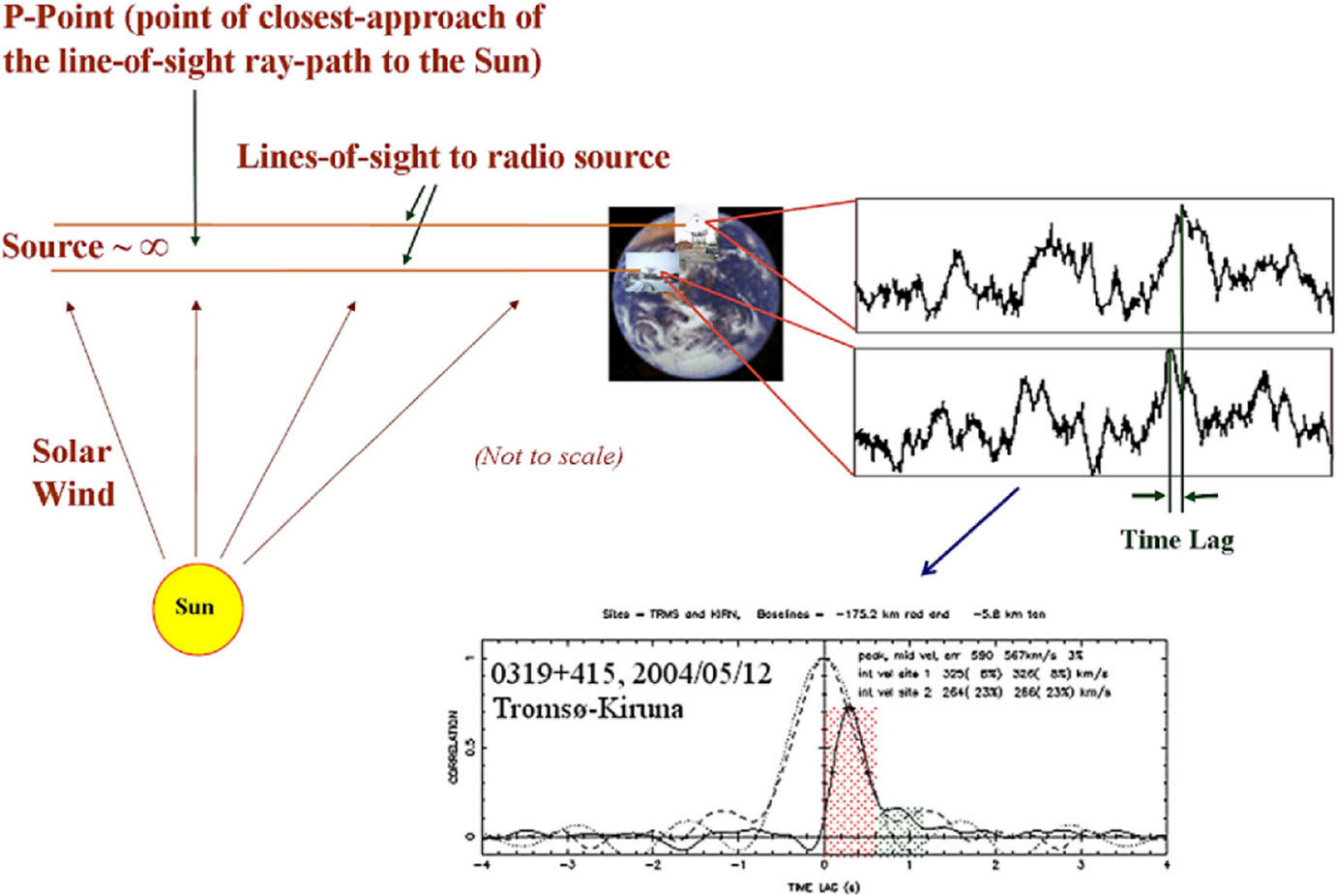
Schematic of IPS observations by two EISCAT stations viewing the same astronomical radio source. Cross-correlation of the signals from the two stations gives two peaks (bottom) associated with solar wind of different speeds. Image reproduced with permission from Bisi et al. (2010), copyright by the authors

## Remote sensing observations of stream interaction regions

### Using interplanetary scintillation observations

**Fig. 72:**
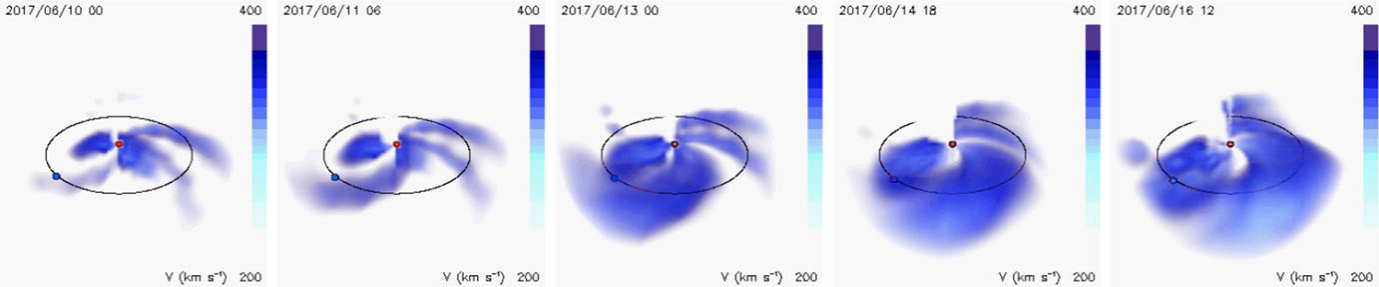
A sequence of tomographic reconstructions of the solar wind speed using IPS observations, from a viewpoint at 3 AU, $30^\circ$ above the ecliptic and $45^\circ$ ahead of the Earth, for June 10–16, 2017, from the UCSD CASS IPS website (http://ips.ucsd.edu/)

Interplanetary scintillation (IPS) occurs during observations of a distant astronomical radio source when solar wind plasma crosses the line of sight, a phenomenon first identified by Hewish et al. (1964). The scintillations in turn can provide information on solar wind conditions (density and speed) along the line of site between the source and Earth (Hewish 1989). As noted in Sect. 6, IPS observations provided evidence of high-speed solar wind over the poles of the Sun at solar minimum before the Ulysses spacecraft confirmed this with in situ observations. Observations of stream interaction regions using IPS have been discussed, for example, by Breen et al. (1998) and Bisi et al. (2010), and the reader is referred to these papers for more details. Figure 71 from Bisi et al. (2010) illustrates how IPS observations from two EISCAT stations (Tromsø and Kiruna) viewing the same distant radio source simultaneously can be combined to infer solar wind speeds. Since the amplitude of scintillation signal from a plasma element falls off with distance from the Sun as $1/R^4$, the line-of-sight scintillation is dominated by plasma closest to the Sun. Plasma elements moving away from the Sun will cross the lines of sight, and modulate the radio signal received at each station in succession. Cross-correlation of the signals from the two stations gives two peaks with different time lags, which indicate the presence of two plasma flows with different speeds. By monitoring IPS along lines of sight to a large number of radio sources, the spatial and temporal variation of slow, intermediate and fast solar wind can be inferred. Such observations may be combined using computer-aided tomography (e.g., Kojima et al. 1998; Asai et al. 1998; Jackson et al. 1998). Figure 72 shows a sequence of views of the solar wind speed distribution in the heliosphere based on the tomography of IPS observations from STELab in Nagoya, Japan, available on the University of California San Diego Center for Astrophysics and Space Science website (http://ips.ucsd.edu/) that show several corotating structures. Animations extending from 6 days to 1 day prior to the most recently received IPS data are available on this website. In addition, the Community Coordinated Modeling Center at NASA’s Goddard Space Flight Center can run this model “on demand” (https://ccmc.gsfc.nasa.gov/requests/requests.php).

### Using white-light observations

**Fig. 73:**
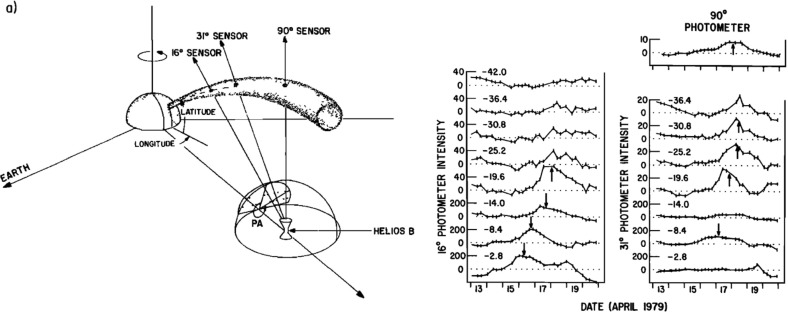
Left: Schematic of the zodiacal light instrument on Helios B, which consisted of three photometers viewing at $16^\circ$, $31^\circ$ and $90^\circ$ from the ecliptic on the spinning spacecraft. Right: Light curves in different viewing directions for the three photometers interpreted as a corotating density enhancement sweeping across the spacecraft. Image reproduced with permission from Jackson (1991), copyright by AGU

Thomson scattering of white light by electrons in density structures in the corona is routinely used to view the corona and coronal mass ejections, but density variations in the solar wind can also be viewed in this way with sufficiently sensitive instruments. The zodiacal light instruments on the Helios spacecraft, designed to study interplanetary dust, were also found to detect interplanetary plasma clouds (Richter et al. 1982). Jackson (1991) used these observations to infer the presence of corotating density structures in the solar wind. A schematic of the operation of the instruments is illustrated in the left-hand panel of Fig. 73. The zodiacal light instruments consisted of three photometers viewing at $16^\circ$, $31^\circ$ and $90^\circ$ from the ecliptic. The observations for the $16^\circ$ and $31^\circ$-photometers were also divided into 32 sectors as the spacecraft spun about an axis perpendicular to the ecliptic. The figure shows the configuration for Helios B, with the photometers viewing north; on Helios A they were pointing south. As a density structure (a corotating one is shown) sweeps across the sky, it enhances the brightness in certain look directions for particular photometers, such as shown in the right-hand panel of Fig. 73. The time and direction variations in the observations can then be used to reconstruct the configuration and motion of this structure. Jackson (1991) identified over 40 such structures in 1976–1979 ranging from low latitudes to as high as $50^\circ$, though he concluded that they were associated with streamers rather than with interaction regions.

**Fig. 74:**
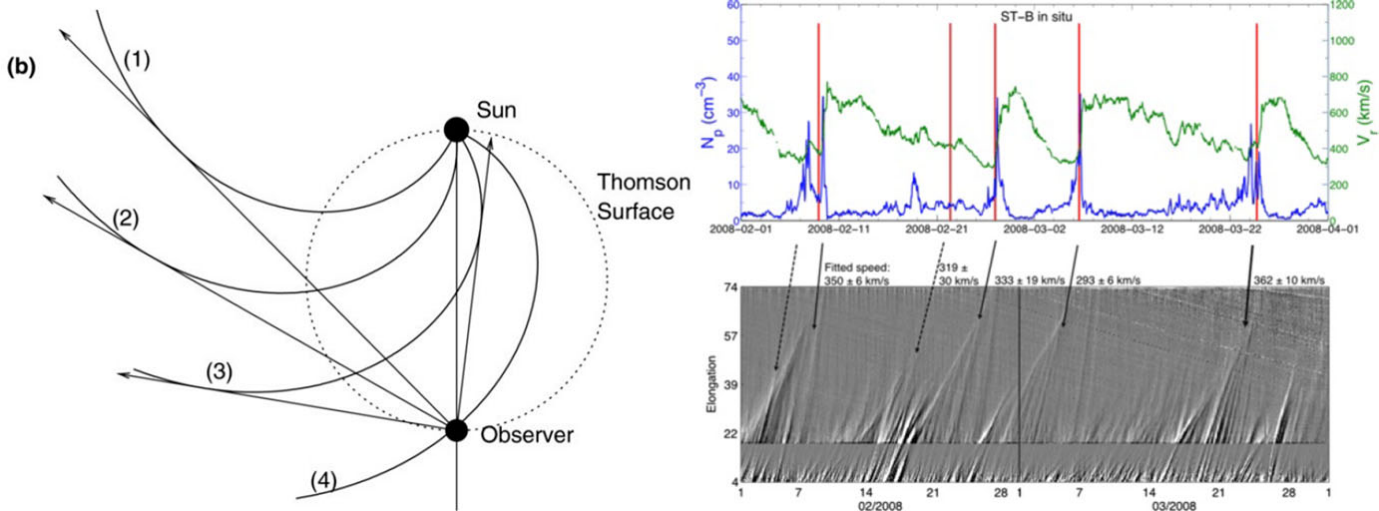
Left: A corotating interaction region approaching an observer, showing the directions to the leading edge at four times and the Thomson surface on which white-light scattering to the observer is optimal. Right: Examples of interaction regions seen in situ at STEREO B and the corresponding density enhancements in a white-light “J-plot”. Images reproduced with permission from [left] Tappin and Howard (2009), copyright by AAS; and [right] from Plotnikov et al. (2016), copyright by the authors

The Solar Mass Ejection Imager (SMEI) on the Coriolis spacecraft, launched on January 26, 2003, and deactivated on September 28, 2011, viewed nearly the whole sky in visible light from a polar orbit and was able to track interplanetary disturbances out as far as 3 AU; see Howard et al. (2013) for a retrospective review of SMEI. Also, the SECHII instrument on each STEREO spacecraft (Howard et al. 2008) includes a heliospheric imager (HI), which observes white light over a large field of view extending out to the orbit of Earth. The left-hand panel of Fig. 74 shows a schematic of how the leading edge of an approaching interaction region might be viewed at four times (1–4). The motion of an interaction region is very different from that of a CME moving out from the Sun, the leading edge appearing first in the east, and then moving only slowly over several days. By time 3, the leading edge also appears in the west close to the Sun. Rouillard et al. (2008) first observed a corotating interaction region in white light using the HIs, while Tappin and Howard (2009) were able to identify an interaction region in the STEREO A and B HI and SMEI data. They also note several factors that cause interaction regions to be more difficult to discern in white light compared to IPS observations: (1) Interaction regions move slowly across the sky in white-light observations and may be suppressed in the usual short baseline background subtractions, which favor fast moving structures; (2) As shown in Fig. 74, the leading edge of the interaction region lies away from the Thomson surface, where scattering of white light towards the observer is geometrically optimal. Also, white-light emission has a $1/r^2$ fall off due to plasma density and another due to incident illumination; and (3) white-light imagers measure integrated line-of-sight density, whereas IPS measures fluctuations in the density. Since there is enhanced turbulence in interaction regions, as discussed above, they may be easier to detect in IPS.

Observations of interaction regions in white light are also discussed by Plotnikov et al. (2016) who have compiled a catalog of 190 “corotating density structures” seen by HI. The right-hand panel of Fig. 74 relates in situ observations of several interaction regions at STEREO B with the corresponding signatures of density enhancements in a white-light “J-plot”; see Plotnikov et al. (2016) for details on how this plot is constructed. In addition, Conlon et al. (2015) discuss observations of 40 interaction regions identified in H1 data. Finally, Jackson et al. (2011) review the methods used to infer the three-dimensional structure of the solar wind from IPS and white-light remote observations.

## MHD modeling of interaction regions

We finally return to the MHD modeling of the solar wind, including interaction regions, an example of which was shown in Fig. 1. Currently, the NOAA Space Weather Prediction Center use the ENLIL global time-dependent 3-D MHD model (Odstrčil 2003) to provide a forecast of interplanetary conditions (http://www.swpc.noaa.gov/products/wsa-enlil-solar-wind-prediction) and also use as a background into which to launch CMEs. ENLIL is driven by photospheric synoptic magnetograms, which are used in conjunction with a suitable model (e.g., the potential field source surface model, Schatten et al. 1969, or “Magnetohydrodynamics outside A Sphere” (MAS) model, Lionello et al. 2009) to generate a coronal magnetic field configuration. Regions of open field (coronal holes) are identified and a relationship between the field expansion and the speed of the solar wind emitted by the coronal hole (“Wang–Sheeley–Arge” model, Arge and Pizzo 2000; Arge et al. 2004) is used to generate a solar wind speed and magnetic field distribution, which is input into ENLIL, usually at $21.5\,R_s$.

**Fig. 75:**
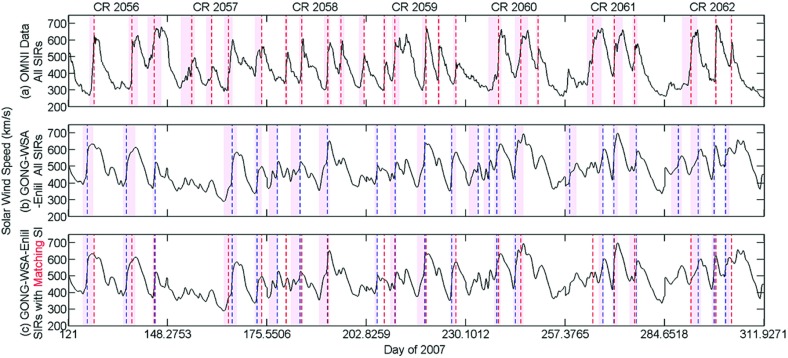
Comparison of the solar wind speed observed (top) and predicted by ENLIL (middle and bottom; see Jian et al. (2015) for details of this model run) during a seven rotation period in 2007. Magenta shading indicates interaction regions and red (blue) dashed lines indicate interfaces. Where there is a reasonable agreement, these are repeated in the bottom panel. Image reproduced with permission from Jian et al. (2015), copyright by AGU

**Fig. 76:**
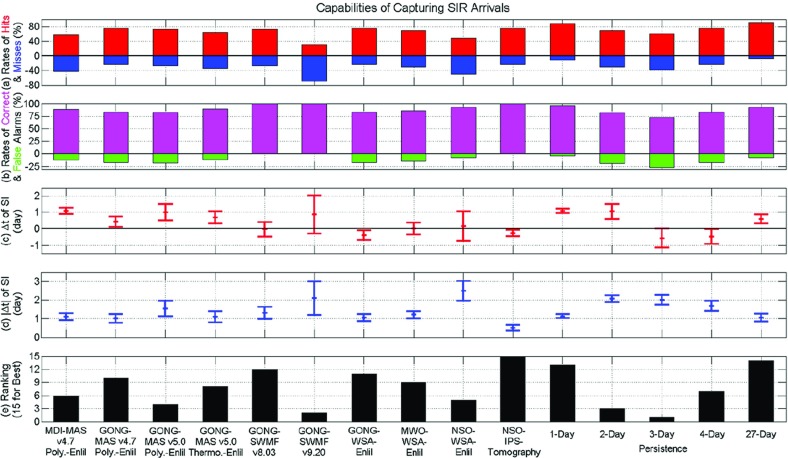
Summary of the validity of stream interaction region predictions from several solar wind models and from assuming conditions observed 1–4 and 27 days earlier. See Jian et al. (2015) for specific details of the models. The parameters shown are **a** rates of hits and misses, **b** rates of correct and false alarms, **c** the average offset between the predicted and actual stream interface arrival times and **d** the absolute value of this offset, and **e** the “ranking” between the 15 cases considered. Image reproduced with permission from Jian et al. (2015), copyright by AGU

As an example of validating model results with observations, for seven Carrington rotations in 2007, Jian et al. (2015) studied the validity of the solar wind parameters predicted by ENLIL using synoptic magnetograms from various sources as input and different models to obtain the coronal magnetic field configuration. As they note, these choices can significantly impact the predicted solar wind parameters. They also assess other methods, such as using IPS, or simply assuming that observed conditions 1–4 days or 27 days earlier persist, to predict these parameters. Considering interaction regions, Fig. 75 compares the observed solar wind speed (top graph) with an example of an ENLIL prediction of the speed at Earth during these seven rotations. Magenta shaded regions are identified interaction regions, and dashed red (blue) lines are stream interfaces in the observed (predicted) data. In many cases, these are in reasonable agreement, and are indicated again on the predicted speed in the bottom panel, but there are also other features that are present in one time series but not the other. Jian et al. (2015) summarize the success of the different models at predicting the arrival of stream interfaces in Fig. 76. There is not sufficient space to describe all these models in detail, but the panels show for each model (a) the rate of “hits” and “misses”, (b) the rate of correct and false alarms, (c) the average offset between the predicted and actual interface arrival times, (d) the absolute value of the offset, and (e) the “ranking” between the 15 cases considered. IPS is the highest ranked, though Jian et al. (2015) note that because it relies on observations of the solar wind conditions in the heliosphere beyond $\sim 40\,R_s$, it has less predictive capability than models that are driven by solar magnetograms. The next ranked prediction simply assumes that the solar wind conditions are the same as those observed 27 days earlier (“27-day persistence”). Jian et al. (2015) conclude that each model has its own strengths and weaknesses and all make simplifying assumptions that treat the physics in very approximate fashion. There is clearly room for improvement, but even adding new physics to a model does not necessarily improve its performance.

**Fig. 77:**
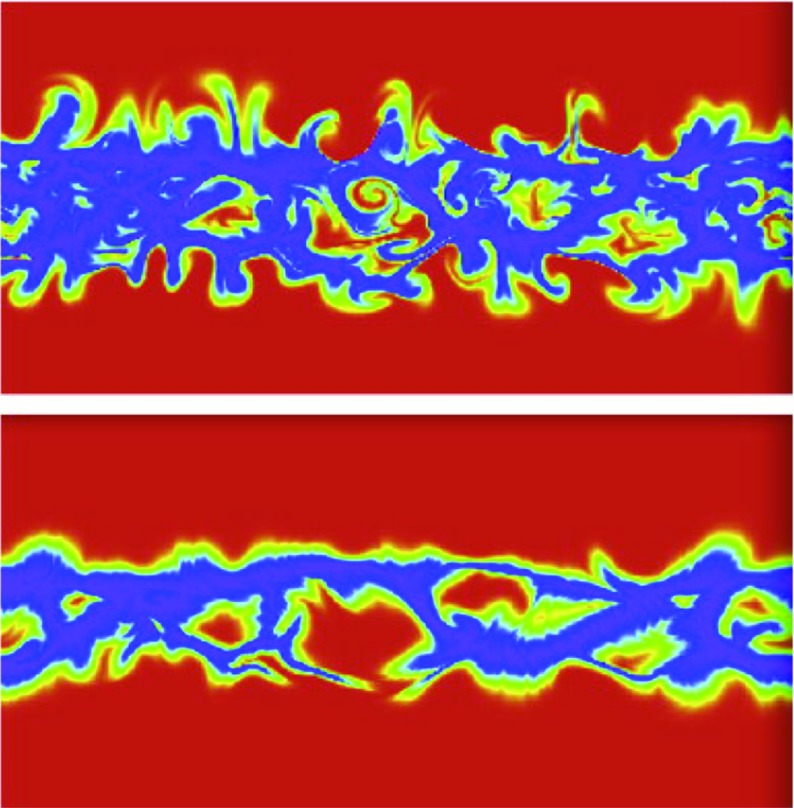
Comparison of radial speed (red $=$ fast, blue $=$ slow) at $30\,R_s$ for (top) high resolution and (bottom) low-resolution simulations of Carrington rotation 2060. See Riley et al. (2012b) for more details

## Outstanding questions concerning stream interaction regions

Notwithstanding the many decades of studying stream interaction regions from the inner to distant heliosphere, there are still a number of outstanding questions (as is obligatory in a paper at this time, it is necessary to mention how the upcoming Solar Orbiter (http://sci.esa.int/solar-orbiter/ and Parker Solar Probe (http://parkersolarprobe.jhuapl.edu/) missions may answer some of them):

- What is the latitudinal structure of interaction regions in the inner heliosphere? Ulysses has observed interaction regions out of the ecliptic beyond 1 AU, but observations within 1 AU have been confined to within a few degrees of the ecliptic though these have provided tantalizing evidence of considerable latitudinal structure. Solar orbiter will probe the region from 0.28 to 0.9 AU at latitudes up to $\sim 30^\circ$.
- Why are interaction regions so variable on small spatial and time scales but also so long-lived?
- How does the structure of interaction regions evolve from the closest approach of the Parker Solar Probe to the Sun ($\sim 10\,R_s$) out to 1 AU?
- How close to the Sun are well-formed interaction regions observed?
- Is a well-identifiable interface present close to the Sun, and how does it develop with distance?
- Figure 77 shows a Carrington longitude versus latitude map of the complex (and model resolution-dependent) structures in the radial solar wind speed at $30\,R_s$ obtained using the model described in Riley et al. (2012b). Will such complex structures be observed by the Parker Solar Probe and Solar Orbiter, and will it be possible to interpret such complexity?
- What are the important acceleration mechanisms for particle acceleration at interaction regions and how can the modeling of particle acceleration in, and transport from, interaction regions progress beyond the incomplete Fisk and Lee (1980) model? Are particles accelerated in interaction regions close to the Sun that may be detected by Parker Solar Probe?
- How do we resolve the problem of identifying the important processes causing cosmic-ray modulation and model this process more realistically? How does GCR modulation vary with latitude in the inner heliosphere?
- Is it possible to predict the strength of geomagnetic activity associated with interaction regions (e.g., using observations at L5), or is this difficult because (1) different Alfvénic fluctuations pass a remote observer and the Earth and such fluctuations cannot be modeled in detail, and (2) interaction regions can show substantially different structures at different locations,
